# The dynamics of fishing villages along the South Atlantic Coast of North America (ca. 5000–3000 years BP)

**DOI:** 10.1038/s41598-024-55047-z

**Published:** 2024-02-26

**Authors:** Victor D. Thompson, Karen Y. Smith, Matthew Sanger, Carey J. Garland, Thomas J. Pluckhahn, Katharine Napora, Jennifer Dodd Bedell, Carla Hadden, Alex Cherkinsky, Rachel Cajigas, Elliot H. Blair, Anna M. Semon, David Hurst Thomas

**Affiliations:** 1 Department of Anthropology, University of Georgia, Athens, USA; 2 Heritage Trust Program, South Carolina Department of Natural Resources, Columbia, USA; 3 Smithsonian Institution, National Museum of Natural History, Washington D.C., USA; 4 Laboratory of Archaeology, University of Georgia, Athens, USA; 5 Department of Anthropology, University of South Florida, Tampa, USA; 6 Department of Anthropology, Florida Atlantic University, Boca Raton, USA; 7 Georgia Department of Community Affairs, GASHPO, Atlanta, USA; 8 Center for Applied Isotope Studies, University of Georgia, Athens, USA; 9 Department of Anthropology, University of Alabama, Tuscaloosa, USA; 10 American Museum of Natural History, New York, USA

**Keywords:** Environmental social sciences, Environmental impact, Psychology and behaviour

## Abstract

We present new chronologies that inform the timing and tempo of shell ring and shell mound construction on the South Atlantic Bight. Our project combines recently acquired dates with legacy radiocarbon dates from over 25 rings and mounds to provide a higher-resolution chronology regarding the occupation and formation of this larger landscape of the earliest fishing villages along the East Coast of the United States. We resolve the ordering and timing of occupation of these rings and mounds through Bayesian statistical modeling. These new models historicize and contextualize these shell rings in ways previously impossible. Specifically, our new chronologies of these villages indicate that the earliest villages were established prior to the invention of pottery. The early period of village establishment evidences isolated village rings, whereas later periods seem to have more villages, but these appear to have been relocated to other areas and/or islands over time. Shell mounds are fewer in number, are spread throughout the time period, and may represent special purpose sites compared to shell-rings. Once villages spread, they quickly adopted new technologies (i.e., pottery) and created new institutions and practiced village relocation, which allowed this way of life to persist for more than a thousand years.

## Introduction

The shift toward long-term, group-oriented cohabitation tied to a specific place on the landscape, or what archaeologists call “villages,” is a foundational shift in human history^1–3^. In the literature, there are two archaeological areas of study regarding village life that capture the attention of researchers. The first area is the initial shift toward sedentism in a region and its attendant effects^4,5^. Literature on this aspect of sedentism often focuses on archaeological indicators (e.g., archaeobotanical or faunal remains) for year-round settlement and internal dynamics^6,7^. The second common area of study examines settlement patterns, site histories, settlement, and abandonment, as well as external dynamics and the environment^8–10^. This work looks at village dynamics once villages become an established pattern on the landscape. Although there is often overlap in individual studies, this separation tends to also sort out by the economic resources that these villages pursue, with nondomesticated resources (or the transition to crop agriculture) being associated with the former and crop reliant villages with the latter^11^. Few studies examine the nature of early village societies where nondomesticated resources provide the economic base beyond looking at evidence for sedentism at the site level. Consequently, case studies of early villages tend to sort out not just by economies but also in terms of methodologies. To correct this, we need additional studies of early villages that take a more regional perspective of dynamic processes across landscapes—not just evidence for sedentism. In short, we need to understand not only how long people occupied early villages before they were depopulated but also what this process looked like in terms of its tempo at the site level as well as at a regional scale.

As Feinman and Neitzel^2^ note, the act of settling down is fundamentally a social process and not one solely driven by environmental conditions or the development of domestic crop agriculture. In brief, the shift to sedentism had multiple pathways, could vacillate, had unintended consequences, and developed in concert with people’s ability to still move around the landscape^2^. Following their model, to understand the variability in the histories of village formation, we must consider not just village formation but also how the institutions of village life endured and were adjusted over time.

The American Southeast provides an excellent place to consider the social dynamics and institutions associated with early villages. One reason is that, based on our current understanding, villages here persisted where groups either never adopted domesticated crops or added them much later as a supplement to an already established economy rooted in estuarine resources^12^. Furthermore, village life both along the coasts and in the interior accompanied a host of other traditions and institutions, some of which lasted thousands of years and operated under different kinds of economies^13–15^. These early villages in the Southeast speak to broader global histories where the emergence of village life and sedentism was situated in coastal and island environments^16,17^.

**Figure 1:**
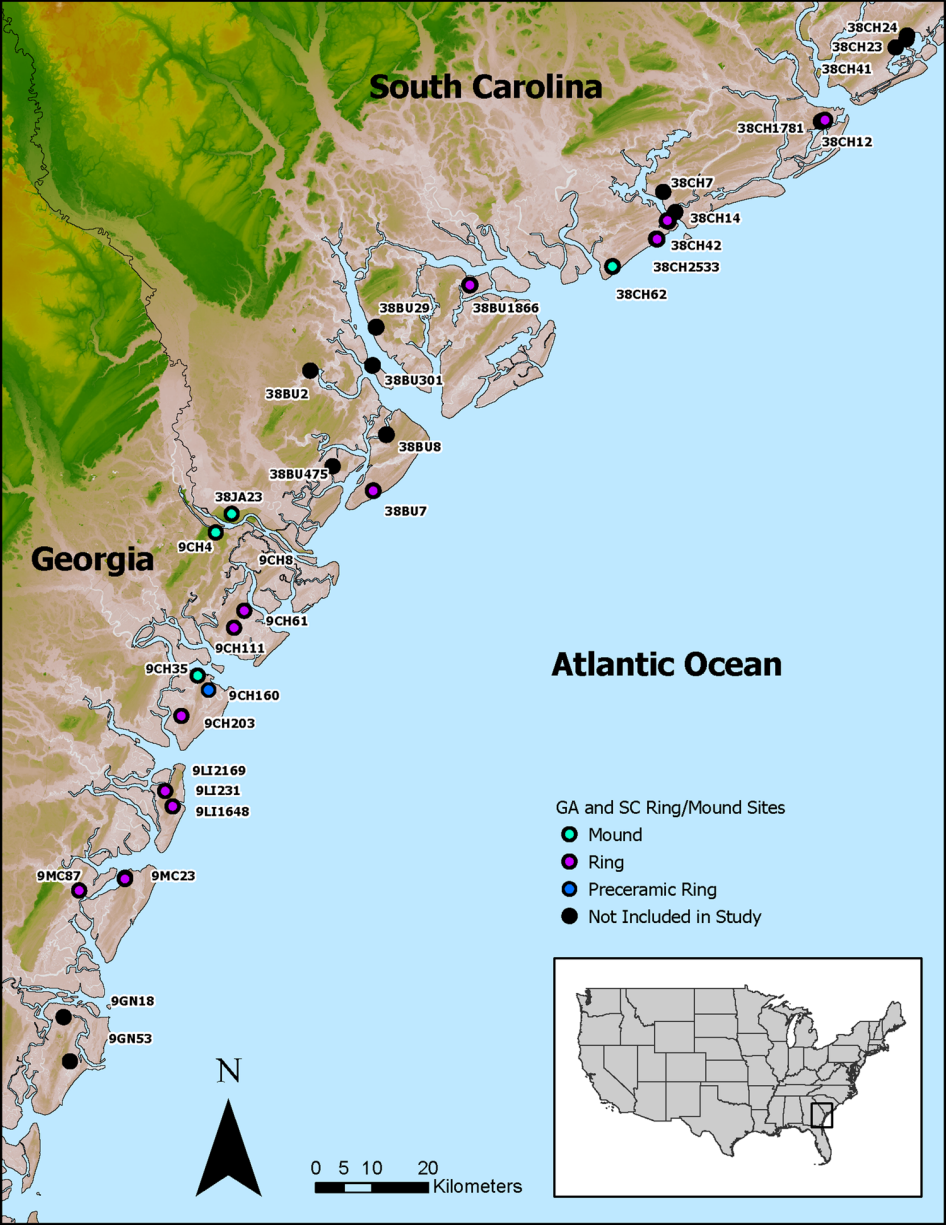
Map showing location of shell-mound and shell-ring sites that are included in the study, as well as sites not included in the study.

**Figure 2:**
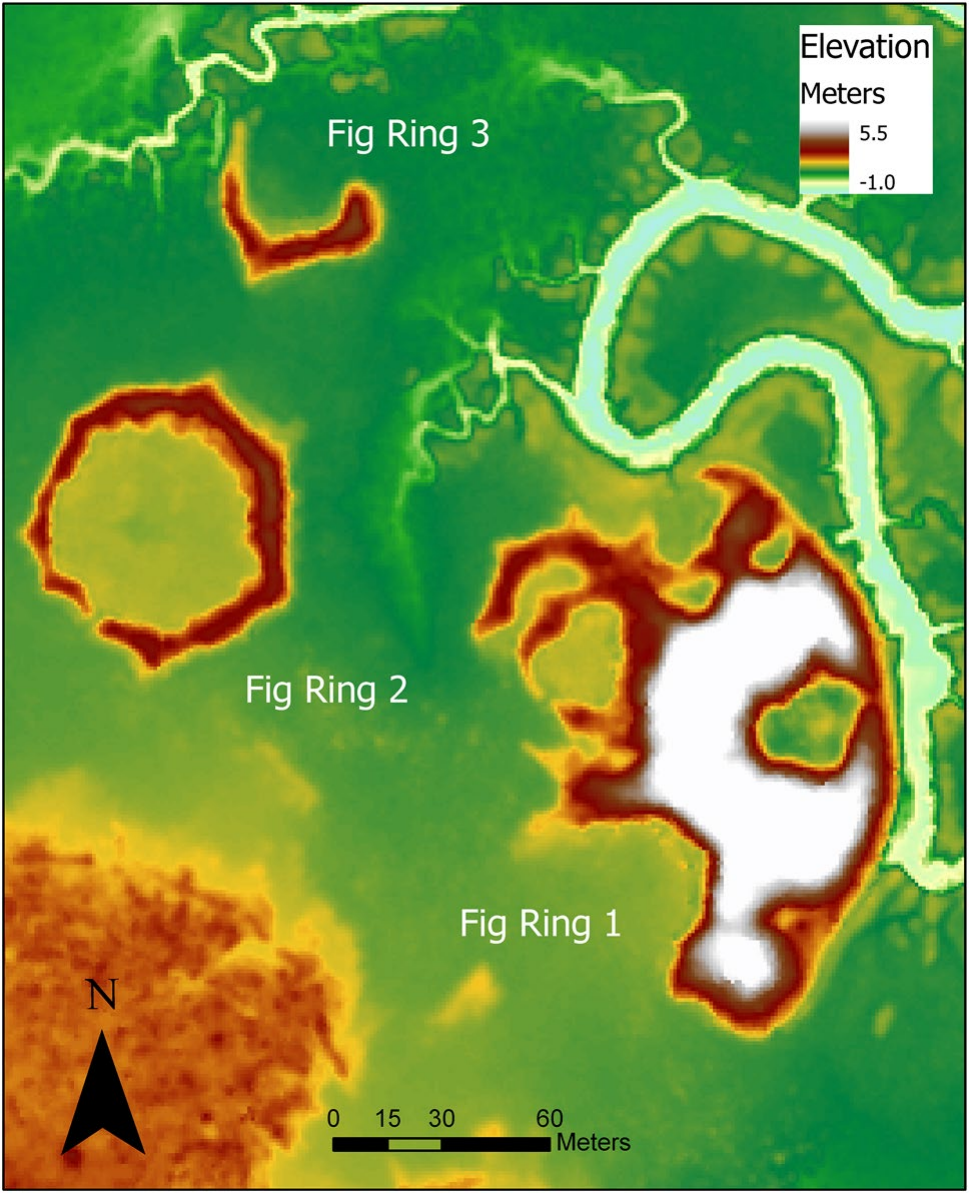
LiDAR image of Fig Island Shell Ring Complex (38CH42). The map was created by Carey Garland using ArcGIS Pro and publicly available LiDAR data from NOAA (https://coast.noaa.gov/dataviewer/#/lidar/search/).

To consider the nature of early villages in the region, we examine the generational histories of shell-ring villages and several associated shell mounds along the South Atlantic Bight (Fig. 1). Shell rings are arcuate to circular deposits of shell, animal bone, and other Native American belongings (e.g., pottery, bone tools, etc.) (Fig. 2). They range in spatial complexity and size from isolated rings and rings with little topographic relief (ca. 50 cm) to multiring complexes, conjoined rings, and rings several meters tall. Although rings are found on both the South Atlantic and Gulf Coasts, we focus on those concentrated on the South Atlantic Bight. Specifically, we present new chronologies on the timing and tempo of the region’s shell rings through Bayesian statistical modeling of 209 dates from 25 rings and shell midden mounds.

These new models of the chronologies of shell rings provide both the foundational historical frameworks that allow us to consider the histories of village life in the region and a frame of reference to examine the emergence of village life, traditions, and institutions in the American Southeast. Based on these new models, we argue that shell-ring villages were a long-lived institution along the coast. Our work further shows that the earliest villages predate the adoption of ceramic vessel technology and the emplacement of specialized shell mounds on the landscape, suggesting residential stability may have played a role in the regional development of these features.

The process of “settling down”^2^ on the coast also was accompanied by the abandonment of certain aspects of mobility and its attendant earlier institutions. That said, isotope data from a number of shell rings^18^ indicates that people continued to practice tethered mobility to harvest dispersed resources from the surrounding estuaries. Furthermore, shell-ring villages seem to have been depopulated, likely for a variety of reasons, every 250 years or less. Consequently, shell-ring inhabitants practiced a type of village relocation/immigration mobility, not unlike what archaeologists observe in other parts of the Eastern Woodlands^8^. In the concluding section of this article, we return to these points and examine the nature of village institutions. We outline a model for the process of village persistence in the region. We then compare these concepts to how we more broadly consider village emergence and persistence in other parts of the world from the perspective of shell-ring villages, that represent some of the earliest villages in Eastern North America.

### Shell rings in the American Southeast

Archaeologists have studied shell rings for over a century and have variously interpreted them as representing a host of different human behaviors^19–25^. The current research at these cultural sites overwhelmingly supports the idea that Native people inhabited shell rings throughout the year, making shell rings the remnants of permanent settlements^26–29^. Further, the distribution of pottery, nonhuman faunal remains, and other artifacts (such as bone pins and fishhooks) suggests that the spatial layouts of these settlements were in fact circular villages. These earliest settlements established a pattern of circular villages, in one form or another, that archaeologists observe across the American Southeast from the time of the shell rings to the coming of European colonizers^30–32^.

Shell rings come in a wide variety of sizes and shapes and are found over much of the southern coasts of the United States (SI Table S1). For the South Atlantic Bight, rings are for the most part arcuate to circular with a cleared interior plaza. The average size of Georgia and South Carolina rings is ca. 60 m in maximum diameter with an average plaza diameter of around 35 m and a shell height and thickness of both two meters (see SI Table S1). Directly along the plaza edge of rings, and sometimes in gaps or breaks of the rings themselves, are feature-rich areas of pits and posts suggesting a habitation zone area, which also contains abundant artifacts, such as early fiber-tempered ceramics^19,21,33,34^.

Shell rings of the American Southeast are most appropriately thought of as fishing villages, as the vast amount of the provisioning for these settlements were harvested from the surrounding estuaries^28,35^. By far the most archaeologically visible component of the rings are the oyster shells (*Crassostrea virginica*), which inhabitants sustainably collected from reefs up to 20 km away^36,37^. The ring deposits also have abundant finfish, including herring and shads, sea catfishes, mullets, and drums being the most ubiquitous. Due to the small size of most of these fishes some sort of mass capture technology was used to harvest them (e.g., finely woven nets, fish weirs)^38^ along with some form of collective labor^12^. Inhabitants also harvested terrestrial resources include nut mast from the uplands as well as sea island mammals^34,35^. Furthermore, zooarchaeological and isotope studies of mollusks provide evidence of year-round occupation of shell rings that is consistent with settled villages. Studies specifically of clams and oysters from multiple rings on both Sapelo and St. Catherines islands provide evidence that inhabitants of these settlements harvested these resources year-round, although for some species (i.e., oysters) there was a preference for collection during the cooler months of the year^18,26^.

While everyday village activities are predominately reflected in shell-ring deposits, this is not to say that different kinds of behaviors did not occur at rings as well. There is evidence to suggest that some deposits may have been piled or mounded along the ring^23,24,39^ from periodic feasts that villages held during ceremonies and larger village gatherings^25,34^. Such feasts were likely a key part of village life^22^ and were likely solidarity feasts, as there is no evidence for status differences in the vertebrate faunal assemblages at sites, such as the St. Catherines Shell Ring^29^. There may have been some degree of situational hierarchy expressed at ring villages as different persons or families hosted feasts and garnered prestige^40^, however, secondary evidence for lasting status differences are not evident in shell-ring assemblages.

In addition to shell rings, Native people of this time frame also created large shell mounds. These kinds of sites are far fewer in number. And, while these sites evidence habitation, they may have also been aggregation sites for villagers in the region. In some instances, these sites have a high frequency and diversity of decorated pottery suggesting use by multiple groups or large-scale gatherings of different communities^20^.

As described above, research has largely focused on shell rings from a site-based or single-site-study perspective that tends to focus on stratigraphy, formal artifact assemblage-level observations, and other types of analyses. Radiocarbon dating of shell rings has been modest except for a few examples^41^, and still fewer examples have employed detailed Bayesian modeling to refine site chronologies^42^. Although site-based analyses provide considerable insight (e.g., indicators of year-round occupation), it is difficult to assess the nature of intersite variability solely from such studies. Accurately placing these rings and mounds in time is important to understanding their purpose. We argue that one of the key pieces of information missing from studies of shell rings from the US Southeast is a detailed understanding of shell-ring site histories from a regional perspective. Consequently, we initiated our current project to create a high-resolution chronology of the occupation of the shell rings of the South Atlantic Coast. The results of this study, as we note above, not only provide a way to assess which settlements were contemporaneous but also lend insight into village movement that can only be observed at a regional scale.

## Results

**Figure 3:**
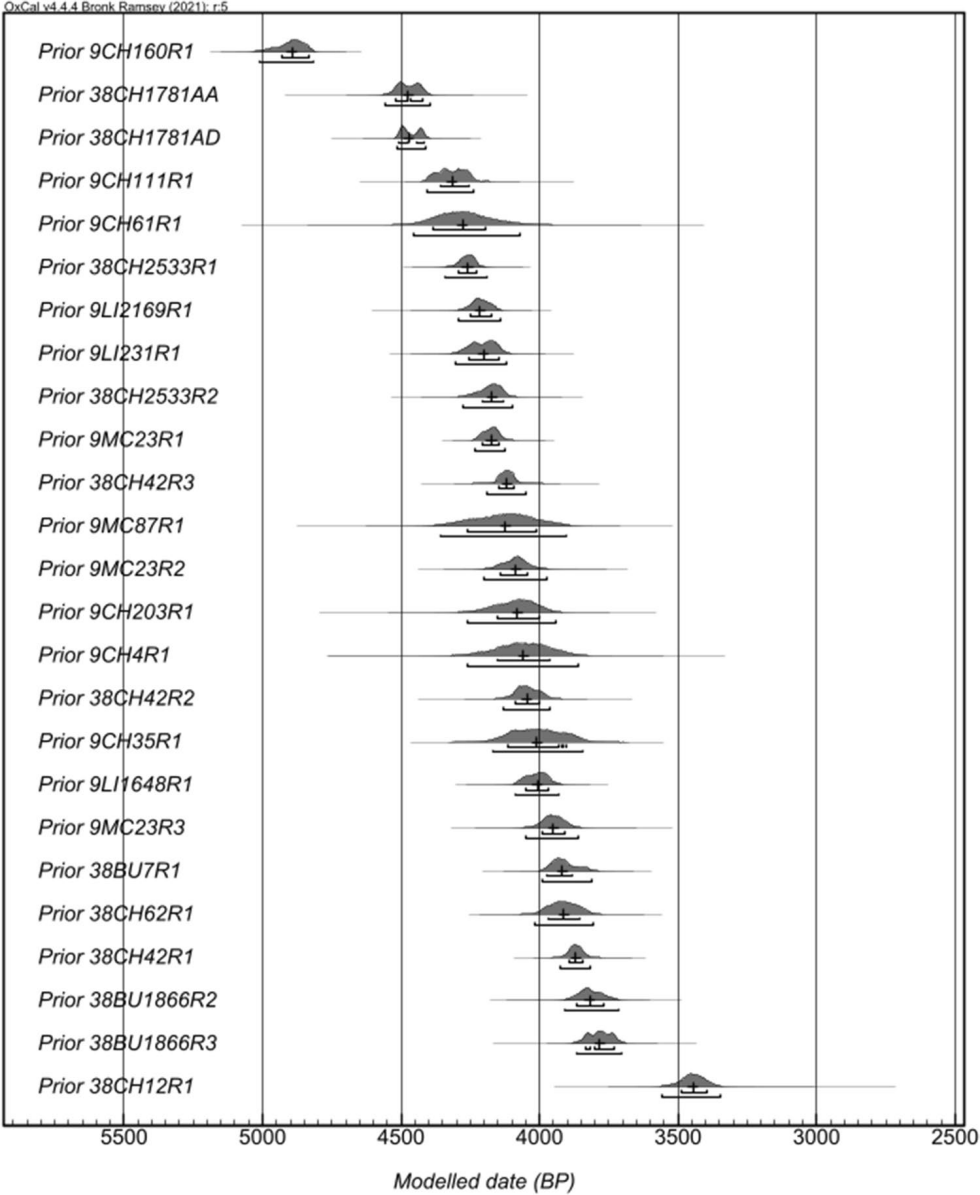
Modeled KDE Plots for each of the ring/mound model for the South Carolina and Georgia coasts, with brackets for the 68% and 95% hpd and crosshairs indicating the median (created by Victor Thompson using OxCAL 4.4).

**Figure 4:**
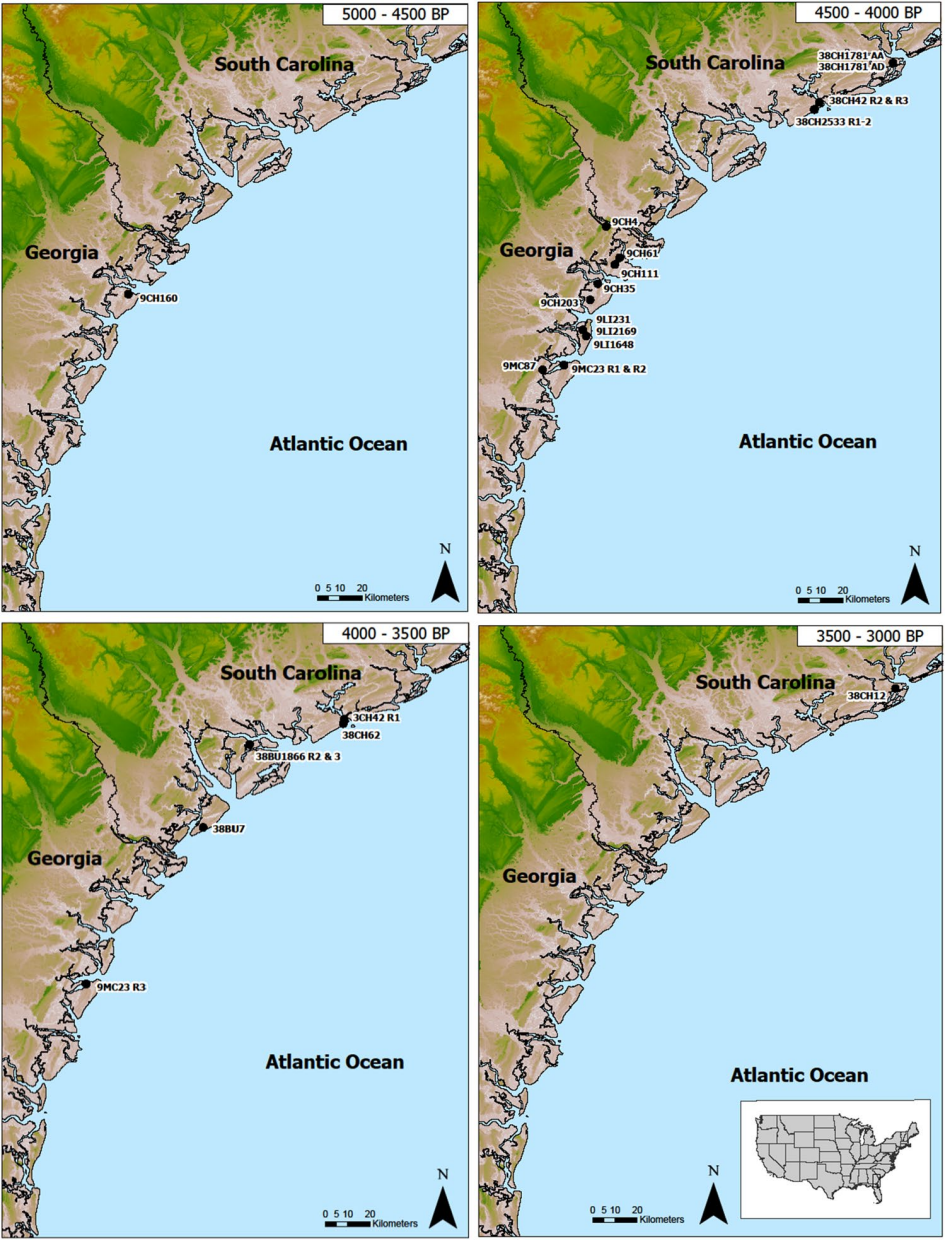
Map of sites included in the study divided into overlapping time frames. Sites were put into these groups based on the modeled medium probability. The map was created by Carey Garland using ArcGIS Pro and publicly available shapefile data from Georgia GIS Clearinghouse (https://data.georgiaspatial.org/index.asp).

An overview of study samples and contexts are presented in SI Table S2, and detailed results of all the models and the estimated age ranges of all modeled shell rings are presented in SI Tables S3–S104. Both the start and end boundaries as well as the KDE Plot for the Phase of each ring’s occupation clearly demonstrate that not all groups inhabited rings contemporaneously (Fig. 3). The results of the “Order” command on the KDE Plot intervals provide probability estimates at the 68% hpd and 98% hpd for the occupation of each ring against one another (Table 1). Here, we show the order of the rings in four overlapping time periods, from earliest to latest, for the purpose of visual presentation (Fig. 4a–d). This does not mean, however, that the occupation of a given ring is tied to a specific phase. The earliest and latest of these time periods contain rings that are either much earlier or later than the others, and they are marked as such on these maps with their corresponding date estimates. Furthermore, both the earliest and latest time frames indicate that people occupied these settlements at different time frames, sometimes separated by hundreds of years. The middle time frames, however, have multiple overlapping and contemporaneous villages (SI Tables S105–S106). These visualizations of the modeling and quantitative analysis of the order of rings show that ring locations shift over time, and frequently, villagers establish shell rings on islands with former villages, sometimes in the same general location as those previously inhabited places.

**Table 1:** Modeled KDE plot distributions used in the order analysis for shell rings and mounds.

Name	Modelled (BP)				
	Median	From 68%	To 68%	From 95%	To 95%
Prior 9CH160R1	4890	4940	4830	5020	4810
Prior 38CH1781AA	4470	4520	4420	4560	4390
Prior 38CH1781AD	4470	4510	4410	4520	4400
Prior 9CH111R1	4320	4360	4250	4410	4230
Prior 9CH61R1	4280	4390	4190	4460	4070
Prior 38CH2533R1	4260	4290	4220	4350	4190
Prior 9LI2169R1	4210	4250	4170	4290	4130
Prior 9LI231R1	4200	4260	4140	4310	4110
Prior 38CH2533R2	4170	4210	4120	4280	4100
Prior 9MC23R1	4170	4210	4140	4240	4120
Prior 38CH42R3	4120	4150	4090	4190	4040
Prior 9MC87R1	4120	4260	4010	4360	3900
Prior 9MC23R2	4090	4140	4040	4200	3970
Prior 9CH203R1	4080	4160	3990	4260	3940
Prior 9CH4R1	4060	4160	3960	4260	3860
Prior 38CH42R2	4050	4090	4000	4130	3960
Prior 9CH35R1	4010	4120	3900	4170	3840
Prior 9LI1648R1	4010	4060	3960	4090	3930
Prior 9MC23R3	3950	4000	3900	4060	3850
Prior 38BU7R1	3920	3980	3880	3990	3800
Prior 38CH62R1	3910	3970	3850	4020	3800
Prior 38CH42R1	3870	3900	3840	3930	3810
Prior 38BU1866R2	3820	3870	3760	3910	3710
Prior 38BU1866R3	3780	3840	3720	3870	3700
Prior 38CH12R1	3450	3490	3390	3560	3340

## Discussion and conclusion

Wilson^43^ argues that one of the consequences of settled life is the visible demarcation of space with permanent architecture. In shell-ring villages, shell deposits served for demarcating village boundaries in a visible and long-lasting way. Although evidence for permanent domestic architecture (i.e., houses) inside shell rings is generally lacking, we view the circular arrayed shell piles that make up the rings as the reflecting community structure. To the extent shell ring deposits are “a materialization of structure”^43^, these earliest villages likely denote a fundamental shift in the structure of daily life.

We now have high-resolution chronologies for a larger proportion of the shell rings in the region than we have ever had before. There is still more dating to be done: at least 16 additional rings require high-precision dating, and expanded sampling efforts would help us to refine chronologies for rings with relatively fewer dates than most in our sample. That said, our new work provides direct insight into intersettlement dynamics and the nature of early villages and their continuance on the landscape for over a millennium in a very specific form (i.e., shell-ring communities).

Our shell-ring dating project indicates three key findings:

1. Shell rings began before the advent of pottery in the region. The earliest known ring in the sequence is *Hokfv-Mocvse* (9CH160), the only aceramic ring in our sample, with quartz lithic bifaces being the primary artifact category.

Settling down into coresident villages required a host of new institutions and technologies to make this new way of life viable in the shifting ecological conditions of the South Atlantic Coast. First, we need to acknowledge that Native people had been coming to the coast likely for millennia prior to the establishment of shell-ring villages, as is indicated by early dates on carbonized hickory nuts at some of these settlements^18^. These forays to the coast and the evolving barrier island ecosystem likely aided in an increasing acquisition of knowledge regarding the environment, especially as it related to fisheries’ resources. We see that the shell-ring villages likely emerged as soon as such resources were available at around 5000 cal BP. Consequently, people rapidly shifted to sedentary communities, suggesting that semisedentary settlements in the interior Atlantic Slope likely predated shell rings on the coast^44^. Indeed, given the artifact assemblage of quartz projectile points at the earliest of the shell rings (i.e., *Hokfv-Mocvse* Shell Ring), the middle Savannah River, where we see similar assemblages and emerging house architecture, may have been the source of some of these earliest populations^45^.

2. The early period seems to indicate isolated village rings, whereas later periods seem to have more villages, but these appear to have been relocated to other areas and/or islands over time.

After 4500 cal BP, shell-ring villages were more numerous, and by this time, they had adopted/invented ceramic container technologies, with pottery being the most abundant artifact recovered from these settlements. Given the size of some of these rings in terms of height and diameter, as well as the fact that some sites have contemporaneous rings^42^, it seems clear that larger coresident populations were becoming the norm.

Our dating and sequencing of shell-ring villages shows that communities, while settling down, did practice some degree of village mobility, which resulted in shifting the location of villages over time—even if the villages were long lived (ca. 250 years). Whether paired rings were occupied sequentially with overlap or were contemporaneous paired villages cannot, in most cases, be determined on the basis of radiocarbon dating alone. In cases where we find that rings are sequential^42^, it remains to be demonstrated that the subsequent occupation is of the same or different groups. We assume that they were related groups, based on geographic and temporal proximity, but other lines of evidence need to be evaluated (e.g., ceramic technology). Despite some uncertainty, we do know that, at least in some cases, either related or different communities returned to inhabit the same island, often locating new shell-ring villages close to older villages. The reinhabiting of old places is a pattern we see all over the world^46,47^. Part of the pattern of island reinhabitation involves place making through daily practice. The fact that shell rings were visible markers of not only past villages but also the resources needed to sustain people in the general vicinity would be a guide for returning groups^48^.

Maintaining social ties and relocating villages likely presented both challenges and benefits to these communities. Some of the challenges we detail above. In addition, with these shifting villages, maintaining connections would have been difficult. Forging new ways of kinship and identity was one avenue of dealing with such stress. We note that more recently, the concept of “tribal town” and “clan” are ways that Ancestral Muskogeans and modern Muscogee people identify to help maintain relationships with geographically distant relatives and community members^49^. We are not saying that we have identified such institutions in the archaeological record here. We merely point to this parallel in the modern descendants of the people who lived in the region as an example of how some have navigated a similar challenge. Finally, the benefits of these kinds of interdependencies among different groups of people spread out across the landscape are the fostering of cooperation and effective management of resources and risk against shortfalls^50^. Ultimately, the socio-ecological relationships formed in villages were flexible enough that even when we see the collapse of the fisheries (ca. 3800 cal BP), people are still able to navigate these environments^51,52^. Although after this point in time, there are few observable shell-ring villages and community structure seems to shift to alternate forms^52^.

3. Shell mounds are fewer in number, are spread throughout the general time period, and may represent special purpose sites (contrary to earlier ideas) compared to shell-ring sites.

As Pearson and colleagues^53^ point out, the transition of village life presented considerable challenges. Specifically, they identify finding appropriate marriage partners as one of the key challenges. In Southwest Asia, this problem was solved with the emergence of megasites that could sustain endogamous marriage partnerships due to their large populations. Although shell-ring settlements were quite large, there are no sites along the coast that would be analogous to the megasites of Southwest Asia.

Given that there are no megasites along the South Atlantic Coast, how then did coastal villagers deal with the challenge of finding exogamous marriage partners? We suggest that there are two different ways that this problem may have been mitigated along the coast. The first is the development of large midden-mound sites such as Cane Patch, where shell was piled up or mounded as opposed to being deposited in a ring form. We suggest that such places may have been aggregation spots for shell-ring communities, allowing for information exchange and social interactions beyond the confines of the village. The other possible way was through the hosting of feasts and ceremonies at shell-ring villages. There is evidence at these settlements for the large-scale consumption of foods that are interpreted as communal feasts^22,26^. As Wallis and Pluckhahn^54^ demonstrate for later villages in the American Southeast, people maintained connections among a broader community landscape. This too fits well with the evidence that indicates that shell-ring communities were matrilineal and that intermarriage among villages linked disparate communities^27^. If this holds, then such a shift in recognizing kinship may have been an institution preferred by early villagers. Consequently, this would create strong interdependencies among shell-ring villagers as a whole^12^.

These three key findings provide important information regarding how the village as an institution was first instigated in the region and how it became embedded within the greater culture landscape in keeping with earlier research^48,52,55^. Each of these findings above provides greater context for understanding the process of settling down in this region and how the institution of villages at both a local and regional level was sustained for such a long time. It would also seem that the way archaeologists envisioned interaction (e.g., rings as primarily ceremonial sites) at shell rings was in part limited by the fact that we did not understand the precise histories of these places. Our new chronologies provide a much-needed platform to assess the social histories of shell rings and allow for a more detailed look at how Native people between 5000 and 3000 years ago constructed community and maintained tradition. This, in turn, allows us to demonstrate the unique ways that Native American groups of the American Southeast (e.g., the Ancestral Muskogeans) met the challenges of living in village communities.

## Methods

Critical to our understanding of shell-ring chronologies is a careful and considered dating program. We outline here our general methods for site selection, the types of materials that we selected for radiocarbon dating, and the Bayesian modeling protocols and reporting guidelines we followed in our study. We provide additional AMS laboratory methods for newly run samples, and site-level detailed methods for modeling and sample context in the supplemental materials (SI Tables S2–106).

### Site and sample selections

A subsection of the South Atlantic Bight comprises our sampling region—roughly the South Carolina and Georgia coasts. There are over 48 recorded shell rings and “large” shell mounds in this study region. We first examined the extant radiocarbon dates reported in various databases^56^ and compiled all the known dates from this region. From this selection, we then focused on sites with known collections in repositories or that project participants—especially those that Thompson, Smith, and Sanger had either worked on or had detailed knowledge about and at which they had conducted recent excavations as part of ongoing research programs. From the total number of shell rings and large shell-midden mounds in this region, we intensively sampled and modeled 25 rings and mounds, which represent over 50% (n = 15) of the sites (n = 29) with known shell rings and large midden mounds in the study area.

Despite the opportunistic targeting of shell rings and shell midden mounds in the study area, our dating program has collated and run a total of 209 AMS radiocarbon dates for our study sites. To facilitate Bayesian modeling (see below), we prioritize sample sequences from stratigraphic contexts to form the “a priori beliefs” that work to constrain the dates in these models.

### Chronometric hygiene and sample materials

After compiling all the extant dates for shell rings and midden mounds in the region, we then performed stringent chronometric hygiene on the legacy dates. Our main criteria for excluding dates from our analysis were (1) if they had large standard deviations greater than 100 years or (2) if they were run on marine shell. This excluded many previously run radiocarbon dates. For example, of the 24 previously available dates from 15 rings and mounds in South Carolina, only four dates from one ring are from carbonized wood, with standard deviations of more than 100 years^33^.

We based our decision to exclude marine-shell radiocarbon dates for three primary reasons. First, as Hadden et al.^57^ state, “Calibrated shell dates are inherently less precise than most terrestrial dates due to uncertainty in ΔR,” which “limits the types and time scales of observable human behaviors”. Because our goal was to resolve the chronologies of shell rings at a level that would allow us to evaluate contemporaneity of these settlements, we rejected using shell on the grounds that it would not provide the necessary accuracy to achieve our research goals. Second, the Georgia and South Carolina coast ΔR from northeast Florida to South Carolina is variable due to the dynamic hydrologic environments of the region. Third, the issue of charcoal, carbonized plant materials, and wood being problematic due to extended preservation does not apply generally to the region. In short, the “old wood” effect does not have the same impact on subtropical regions as it does on arid ones. In the former, organic materials degrade quickly and usually do not survive for extended periods of time to be used as fuel wood^58^. Consequently, it is unlikely that fuel wood used by inhabitants of shell rings sat around on the surface for years before being collected. However, wood and wood charcoal from an older part of a long-lived tree will have an “inbuilt” age that predates its use by people. The Bayesian framework is well suited to accounting for the possibility of both the “old wood” effect and the “inbuilt age” of wood and wood charcoal^59,60^. Last, some archaeologists are under the general assumption that carbonized materials are somewhat scarce in shell middens^57^. This is not the case for the region under study, which has sufficient carbonized materials present in most levels of site excavations.

After excluding both extant dates with large errors and all marine shell dates, we began selecting additional samples to date from our ongoing and collections-based research. We primarily choose samples that represent short-lived carbonized plant remains, focusing particularly on hickory nuts (*Carya* spp.). These nut fragments are ubiquitous at sites throughout the region, and we commonly recover these carbonized remains throughout the middens. In situations where hickory nut was unavailable, or in the case of older collections, when ethnobotanical remains were not saved as part of the excavation process, we relied upon white tailed deer (*Odocoileus virginianus*) teeth or long bones, or carbonized wood remains.

### Bayesian modeling

Multiple model variants were run for each site to ensure robust results that were not overly sensitive to the a priori beliefs. At a minimum, for each ring or mound, we ran (1) a “standard” model that accounted for stratigraphic information, including the relative ordering of dated samples within a stratigraphic sequence; (2) a variant that included a general outlier model, which identifies and downweights temporal outliers; and (3) a variant that included both general and charcoal outlier models, the latter of which specifically accounts for the possibility of “old wood” and the “inbuilt age” of wood charcoal. More than 104 models in total were run.

The starting and ending boundaries for each ring and mound were then used as priors to build a model to quantitatively evaluate the sequence of these sites by using the Order command in OxCal 4.4.4 and the IntCal20 curve^61^. We constructed a final model using a log-normal Interval command and used the KDE Plot command to provide a date range for an occupied ring or mound, which was also subject to the values from the KDE Plot command to return probability estimates of the order of cultural sites. The Log-normal Interval command is based on a 125-year value which effectively constrains the site duration to 250 years. This distribution focuses most of the probability on the earlier part of the distribution, but also allows for exceptions that span longer times with a long tail^62^. This value is based on our survey of the literature of village duration in Eastern North America that places the upper limit of most circular villages (without monumental earthen architecture) as lasting no more than this value based on artifact and architectural evidence^30–32^. Furthermore, the amount of pottery fragments and density of faunal remains at these sites suggests a shorter occupation on the order of 50–100 years^22,28^, therefore, we feel that our estimate of 250 is a conservative upper limit estimation of the length of these villages.

### Supplementary Information

Supplementary Information.

## Data Availability

All data generated or analyzed during this study are included in this published article and its supplementary information files.
