# Supplementary material for: The dynamics of fishing villages along the South Atlantic Coast of North America (ca. 5000–3000 years BP)
Source: Sci Rep. 2024 Feb 26;14:4691. doi: 10.1038/s41598-024-55047-z (PMC10897379; doi:10.1038/s41598-024-55047-z)
Supplement: Supplementary file 1 — Supplementary Information. [file 41598_2024_55047_MOESM1_ESM.docx]

**Supplemental Information for:**

**The Dynamics of Fishing Villages along the South Atlantic Coast (ca. 5000 to 3000 years BP)**

Authors

Victor D. Thompson*, Department of Anthropology, University of Georgia, [vdthom@uga.edu](mailto:vdthom@uga.edu)

Karen Y. Smith, South Carolina Department of Natural Resources, [SmithKY@dnr.sc.gov](mailto:SmithKY@dnr.sc.gov)

Matthew Sanger, Smithsonian Institution, Museum of the American Indian, [SangerM@si.edu](mailto:SangerM@si.edu)

Carey J. Garland, Laboratory of Archaeology, University of Georgia, [crg79217@uga.edu](mailto:crg79217@uga.edu)

Thomas J. Pluckhahn, Department of Anthropology, University of South Florida, [tpluckhahn@usf.edu](mailto:tpluckhahn@usf.edu)

Katharine Napora, Department of Anthropology, Florida Atlantic University, [knapora@fau.edu](mailto:knapora@fau.edu)

Jennifer Dodd Bedell, Georgia Department of Community Affairs, [bedelljenn1@gmail.com](mailto:bedelljenn1@gmail.com)

Carla Hadden, Center for Applied Isotope Studies, University of Georgia, [hadden@uga.edu](mailto:hadden@uga.edu)

Alex Cherkinsky, Center for Applied Isotope Studies, University of Georgia, [acherkin@uga.edu](mailto:acherkin@uga.edu)

Rachel Cajigas, Department of Anthropology, University of Alabama, [rmcajigas@ua.edu](mailto:rmcajigas@ua.edu)

Elliot Blair, Department of Anthropology, University of Alabama, [ehblair@ua.edu](mailto:ehblair@ua.edu)

Anna Semon, American Museum of Natural History, [asemon@amnh.org](mailto:asemon@amnh.org)

David Hurst Thomas, American Museum of Natural History, [thomasd@amnh.org](mailto:thomasd@amnh.org)

**SHELL RING METRICS**

Shell rings come varity of forms and are found all over the coasts of the American Southeast, on both the Gulf of Mexico and Atlantic Ocean sides. Here we have focused on a subregion, the South Atlantic Bight, which includes the whole Georgia Coast, South Caroliana, and the northeastern portion of Florida, and portions of North Carolina. It is noteworthy, that shell rings as we know them do not occur futher north than northern South Carolina. Russo (2006) provides a good summary of shell rings in the Southeast; however, several newly rediscovered shell rings can now be added to his work. Table S1. provides metrics for each of the known rings in the study area from Russo’s compliation and our own refinments using better maps (i.e., newly avilible lidar maps) to make more accurate measuments of ring dimestions, and addtions of rings that did not exist in the known database at the time Russo complied his list.

**Table S1:** Dimsitons and core measurements of shell rings in the study region (adapted and added to from Russo 2006: table 2).

| **Site Name** | **Site Type** | **Site No.** | **Max Diameter** | **Min Diameter** | **Plaza Diameter** | **Height** | **References** |
| --- | --- | --- | --- | --- | --- | --- | --- |
| Sea Pines | Ring | 38BU7 | 60 | 55 | 42 | 1.0 | Calmes 1967; Trinkley 1980:39 |
| Skull Creek, Large | Ring | 38BU8 | 55 | UNK | 24 | 2.1 | Calmes 1967 |
| Skull Creek, Small | Ring | 38BU8 | 43 | UNK | 27 | 2.1 | Calmes 1967 |
| Guerard Point | Ring | 38BU21 | 40 | UNK | 20 | 0.7 | Moore 1898:147 |
| Chester Field | Ring | 38BU29 | 54 | 27 | 30 | 1.5 | Flannery 1943; Ritter 1933 |
| Barrow's | Ring | 38BU300 | 60 | 40 | 22 | 2.0 | Saunders et al. 2006 |
| Patent Point | Ring | 38BU301 | 60 | 54 | 40 | 1.0 | Saunders et al. 2006 |
| Bull Island | Ring | 38CH23 | 62 | UNK | UNK | 0.9 | Bragg 1925; Hemmings 1970a |
| Coosaw 1 | Ring | 38BU1866 | 60 | 55 | 30 | 1.4 | Heide and Russo 2003 |
| Coosaw 2 | Ring | 38BU1866 | 60 | 55 | 30 | 1.4 | Heide and Russo 2003 |
| Coosaw 3 | Ring | 38BU1866 | 60 | 55 | 38 | 0.3 | Heide and Russo 2003 |
| Coosaw 4 | Ring | 38BU1866 | 57 | 53 | 25 | 0.4 | Heide and Russo 2003 |
| Coosaw 5 | Ring | 38BU1866 | 59 | 54 | 25 | 0.3 | This paper |
| Hanckel Mound | Ring | 38CH7 | 62 | UNK | UNK | 2.4 | Russo 2006 |
| Lighthouse Point | Ring | 38CH12 | 76 | 76 | 37 | 3.0 | Drayton 1802; Trinkley 1980, 1985 |
| Horse Island | Ring | 38CH14 | 61 | UNK | UNK | 3.0 | Russo 2006 |
| Buzzards Island | Ring | 38CH23 | 62 | UNK | UNK | 0.9 | Judge and Smith 1991:36 |
| Stratton Place | Ring | 38CH24 | 50 | 40 | 21 | 0.6 | Trinkley 1980; 1985 |
| Auld | Ring | 38CH41 | 56 | 50 | UNK | 1.8 | Dorroh 1971; Judge and Smith 1991:36 |
| Fig 1 | Ring | 38CH42 | 157 | 111 | 30 | 4.7 | Heide 2002; Russo 2002 |
| Fig 2 | Ring | 38CH42 | 82 | 77 | 58 | 1.4 | Heide 2002; Russo 2002 |
| Fig 3 | Ring | 38CH42 | 49 | 44 | 30 | 1.4 | Heide 2002; Russo 2002 |
| Sewee | Ring | 38CH45 | 75 | 61 | 31 | 3.0 | Russo and Heide 2003:31 |
| Oemler | Ring | 9CH14A | 23 | UNK | UNK | 1.2 | DePratter 1991; Waring 1968:182 |
| Cane Patch | Midden Mound | 9CH35 | UNK | UNK | UNK | 3.0 | DePratter 1976:72, 107–109; 1974 |
| Skidaway 9, Large | Ring | 9CH63 | 61 | UNK | UNK | 1.5 | Beasley 1970:118-119 |
| Skidaway 9, Small | Ring | 9CH63 | 30 | UNK | UNK | 1.5 | Beasley 1970:118-119 |
| Skidaway 21 | Ring | 9CH75 | UNK | UNK | UNK | na | Beasley 1970:119, 122 |
| Skidaway | Ring | 9CH77 | 77 | 58 | 35 | 2.3 | DePratter 1975:17; Howard et al. 1980:251 |
| Odingsell | Ring | 9CH111 | 47 | 37 | UNK | 1.5 | DePratter 1975:23-28 |
| Ossabaw Shell Ring | Ring | 9CH203 | 45 | 31 | 27 | 0.9 | DePratter 1974:281 |
| Bony Hammock | Ring | 9GN53 | 30 | UNK | UNK | 2.1 | DePratter 1976:130 |
| Cannon's Point | Ring | 9GN57 | 79 | 46 | 35 | 1.5 | DePratter 1976:131-132; Marrinan 1975:129 |
| West | Ring | 9GN76 | 58 | 43 | 43 | UNK | Marrinan 1975:31, 129 |
| McQueen Ring | Ring | 9LI1648 | 69 | 66 | 36 | 0.4 | Sanger 2015 |
| Musgrove Ring | Ring | 9LI2169 | 60 | 54 | 30 | 0.3 | Cajigas et al. 2024 |
| St. Catherines Shell Ring | Ring | 9LI231 | 70 | 65 | 39 | 0.8 | Sanger 2015 |
| Sapelo I | Ring | 9MC65 | 90 | 75 | 50 | 2.7 | McKinley 1873; Thompson 2006; Waring and Larson 1968 |
| Sapelo 2 | Ring | 9MC65 | 90 | 75 | 55 | 0.9 | McKinley 1873; Thompson 2006; Waring and Larson 1968 |
| Sapelo 3 | Ring | 9MC65 | 52 | 47 | 35 | 0.9 | McKinley 1873; Thompson 2006; Waring and Larson 1968 |
| A. Busch Krick | Ring | 9MC87 | 40 | 18 | 18 | 1.6 | Crusoe and DePratter 1976 |
| Hokfv-Mocvse Shell Ring | Ring | 9CH160 | 61 | 41 | 42 | 1.0 | Garland et al. in review |
| Barbour Island | Ring | 9MC320 | 65 | 25 | UNK | 4.0 | Georgia Site File |
| Pockoy 1 | Ring | 38CH2533 | 60 | 55 | 22 | 0.3 | This paper |
| Pockoy 2 | Ring | 38CH2533 | 73 | 68 | 30 | 0.3 | This paper |
| Parrot Point Area A | Ringed midden | 38CH1781 | UNK | UNK | UNK | UNK | Baluha and Poplin 2005 |
| Parrot Point Area D | Ringed midden | 38CH1781 | UNK | UNK | UNK | UNK | Baluha and Poplin 2005 |
| Spanish Mount | Midden Mound | 38CH62 | 30 | 10 | N/A | 3 | Sutherland 1974 |

**RADIOCARBON AMS ANALYSIS METHODS**

The charcoal and wood samples were treated following the acid/alkali/acid (AAA) protocol, which involves three steps: (1) an acid treatment (1N HCl at 80°C for 1 hour) to remove secondary carbonates and acid-soluble compounds; (2) an alkali (NaOH) treatment; and (3) a second acid treatment (HCl) to remove atmospheric CO_2_. The samples were then thoroughly rinsed with deionized water between each step, and the pretreated sample was dried at 105ºC. For accelerator mass spectrometry analysis, the cleaned samples were combusted at 900ºC in evacuated/sealed quartz ampoules in the presence of CuO.

The recovered CO_2_ was cryogenically purified and converted to graphite on iron catalyst at 580 ºC.  The measurement was performed using National Electrostatics Corporation (NEC) model 1.5SDH-1 Pelletron accelerator capable of accelerating the +1 charged ions to about 1 MeV. This AMS system is designed to measure ^14^C in modern graphite samples with a precision of 0.3 % or better for 1 mg size targets. The high energy ^13^C+ current in this study ranges from 400 nA for 1 mg of carbon down to about 250 nA for the targets of about 300 μg. The sample ratios were compared to the ratio measured from the Oxalic Acid I (NBS SRM 4990). The sample δ^13^C values were measured separately from the small aliquot of carbon dioxide using a stable isotope ratio mass spectrometer Delta V with respect to VPDB.

The quoted uncalibrated dates have been given in radiocarbon years before 1950 (years BP), using the ^14^C half-life of 5568 years. The error is quoted as one standard deviation and reflects both statistical and experimental errors. The date has been corrected for isotope fractionation.

**INDIVIDUAL RADIOCARBON MODEL RESULTS**

To securely date the rings, we ran a total of 209 AMS radiocarbon dates on 25 shell rings on the South Carolina and Georgia coasts (see Table 1). A total of 104 individual models were run for all sites. For each of these shell rings, we constructed a series of Bayesian models in OxCal 4.4.4 (Bronk Ramsey 2009; Buck et al. 1991, 1996) using the IntCal20 14C calibration curve (Reimer et al. 2020). These models are based on our knowledge of the types of samples, their overall contexts, and stratigraphic ordering. Briefly, the construction of models based on *a priori* information allows for further constraints upon the date ranges than simple calibration alone (Hamilton and Krus 2018). Following Manning et al. (2022), commands and functions in OxCal are presented here as capitalized words for clarity (e.g., Date, Phase, etc., see Manning et al. 2022). Models are considered to have good agreement between dates and the stipulated parameters when the A model agreement index is 60 or greater (Bronk Ramsey 1995). Although the Oxcal-generated agreement index is not a statistical measure of a model’s significance, nevertheless it serves to alert the analyst as to the performance of the models (Bronk Ramsey 2009:356). Each of these is discussed separately in this report. The dated occupational intervals are then compared and evaluated statistically regarding their order. All dates are reported in cal. BP and rounded to the nearest 10.

**Table S2:** Radiocarbon samples, contexts, and unmodeled dates. Some of dates listed here are duplicate runs (as indicated by letter at the end of the Lab Number)

| Site Name | Site Number | Lab Number | Additional Context Description | Material | δ13C ‰ | 14C years BP | ± | References |
| --- | --- | --- | --- | --- | --- | --- | --- | --- |
| Sea Pine | 38BU007 | UGAMS_43766 | 38BU007N209E5125.3-5.2 | deer bone | -22.03 | 3650 | 20 | This paper |
| Sea Pine | 38BU007 | UGAMS_43767 | 38BU007N209E5125.1-5.0 | deer bone | -22.31 | 3660 | 20 | This paper |
| Sea Pine | 38BU007 | UGAMS_43768 | 38BU007N209E5125.0-4.9 | nut shell | -23.25 | 3640 | 20 | This paper |
| Sea Pine | 38BU007 | UGAMS_43769 | 38BU007N212E4855.33-5.28 | deer bone | -22.65 | 3490 | 20 | This paper |
| Sea Pine | 38BU007 | UGAMS_43770 | 38BU007N212E4855.0-4.9 | cf. nut shell | -26.94 | 3540 | 20 | This paper |
| Sea Pine | 38BU007 | UGAMS_43771 | 38BU007N184E4795.3-5.2 | nut shell | -24.98 | 3650 | 20 | This paper |
| Sea Pine | 38BU007 | UGAMS_43772 | 38BU007N184E4795.2-5.1 | nut shell | -26.42 | 3620 | 25 | This paper |
| Sea Pine | 38BU007 | UGAMS_43775 | 38BU007N178E5135.2-5.1 | nut shell | -23.54 | 3650 | 20 | This paper |
| Sea Pine | 38BU007 | UGAMS_43776 | 38BU007N178E5135.03-4.9 | nut shell | -25.28 | 3570 | 20 | This paper |
| Coosaw 2 | 38BU1866 | UGAMS_59259 | STP # 5, 50cm sq, N941.5E2063.5, 15-30cmbs | nut shell (Carya) | -24.75 | 3370 | 25 | This paper |
| Coosaw 2 | 38BU1866 | UGAMS_59260 | STP # 6, 50cm sq, N944E2064, 15-30cmbs | nut shell (Carya) | -26.1 | 3530 | 25 | This paper |
| Coosaw 2 | 38BU1866 | UGAMS_63209 | Unit 2, Level 5, 40-50cmbs | wood charcoal (hardwood) | -26.72 | 3490 | 20 | This paper |
| Coosaw 2 | 38BU1866 | UGAMS_63210 | Unit 2, Level 8, 70-80cmbs | wood charcoal (pine) | -26.58 | 3600 | 20 | This paper |
| Coosaw 2 | 38BU1866 | UGAMS_63211 | Unit 2, Level 10, 90-100cmbs | wood charcoal (pine) | -25.03 | 3500 | 20 | This paper |
| Coosaw 3 | 38BU1866 | UGAMS_63212 | Unit 3, Level 3, 20-33cmbs | wood charcoal (pine) | -24.85 | 3540 | 30 | This paper |
| Coosaw 3 | 38BU1866 | UGAMS_63213 | Unit 3, Level 2, 10-20cm s | deer long bone | -21.48 | 3480 | 20 | This paper |
| Lighthouse Point | 38CH12 | UGa_2901 | Sample 1; 230R60, Level 2 | charcoal | -25.00 | 3190 | 70 | Trinkley 1980:191-192 |
| Lighthouse Point | 38CH12 | UGa_2902 | Sample 2; 230R70, Level 2 | charcoal | -25.00 | 3275 | 55 | Trinkley 1980:191-192 |
| Lighthouse Point | 38CH12 | UGa_2903 | Sample 3; Feature 33, south half, Level 2 | charcoal | -25.00 | 3180 | 65 | Trinkley 1980:191-192 |
| Lighthouse Point | 38CH12 | UGa_2904 | Sample 4; Feature 34, north half, Level 2 | charcoal | -25.00 | 2885 | 175 | Trinkley 1980:191-192 |
| Lighthouse Point | 38CH12 | UGa_2905 | Sample 5; Feature 37, north half, ash zone, base of Level 2 | charcoal | -25.00 | 3345 | 70 | Trinkley 1980:191-192 |
| Parrot Point A | 38CH1781 | Beta-178544 | Unit 411 Level 11 Cat 3 | wood charcoal | -25.8 | 5190 | 40 | Balula and Poplin 2005:C-1 |
| Parrot Point A | 38CH1781 | UGAMS_61229 | Unit 411 Level 12 (110-120 cmbs); Bag 621 | nut shell (Carya) | -25.03 | 3950 | 25 | This paper |
| Parrot Point A | 38CH1781 | Beta-178546 | Unit 407 Level 5 Cat 20 | wood charcoal | -25.9 | 4090 | 40 | Balula and Poplin 2005:C-1 |
| Parrot Point A | 38CH1781 | UGAMS_61230 | Unit 412 Level 6 (50-60 cmbs); Bag 593 | nut shell (Carya) | -24.02 | 4000 | 25 | This paper |
| Parrot Point A | 38CH1781 | UGAMS_61231 | Unit 412 Level 7 (60-70 cmbs) | nut shell (Carya) | -27.65 | 3920 | 25 | This paper |
| Parrot Point A | 38CH1781 | UGAMS_61232 | Unit 412 Level 8 (70-80 cmbs); Bag 630 | nut shell (Carya) | -27.44 | 4040 | 25 | This paper |
| Parrot Point D | 38CH1781 | UGAMS61233 | Midden 2, Unit 452 Level 3 (20-30 cmbs) | deer tibia | -22.7 | 4020 | 25 | This paper |
| Parrot Point D | 38CH1781 | Beta-291351 | Midden 2, Unit 452 Level 4 (30-40 cmbs) | bone collagen | -22.4 | 4060 | 40 | Eric Poplin (personal communication, 2022) |
| Parrot Point D | 38CH1781 | UGAMS61234 | Midden 2, Unit 452 Level 4 (30-40 cmbs) | deer molars | -22.14 | 3970 | 25 | This paper |
| Parrot Point D | 38CH1781 | UGAMS61235 | Midden 2, Unit 452 Level 5 (40-50 cmbs) | deer | -22.26 | 3940 | 25 | This paper |
| Parrot Point D | 38CH1781 | UGAMS61236 | Midden 3, Unit 462 Level 3 (20-30 cmbs) | deer long bone | -22.71 | 3950 | 25 | This paper |
| Parrot Point D | 38CH1781 | UGAMS61237 | Midden 3, Unit 462 Level 4 (30-40 cmbs) | deer long bone | -22.48 | 3950 | 25 | This paper |
| Pockoy 2 | 38CH2533 | OXA-41061 | 110F-S-456; Bag 2046 | nut shell (Carya) | -25.44 | 3854 | 19 | This paper |
| Pockoy 2 | 38CH2533 | OXA-41062 | N2140E1020; Bag 2063 | nut shell (Carya) | -26.76 | 3871 | 19 | This paper |
| Pockoy 2 | 38CH2533 | UGAMS_53612a | 110C-S-438; Bag 2042 | nut shell (Juglans) | -27.7 | 3760 | 25 | This paper |
| Pockoy 2 | 38CH2533 | UGAMS_53612b | 110C-S-438; Bag 2042 | nut shell (Juglans) | -27.7 | 3700 | 25 | This paper |
| Pockoy 2 | 38CH2533 | UGAMS_53613 | 110D-S-445; Bag 2043 | nut shell (Carya) | -27.4 | 3730 | 25 | This paper |
| Pockoy 2 | 38CH2533 | UGAMS_53614 | 110E-S-450; Bag 2045 | nut shell (Carya) | -23.4 | 3840 | 25 | This paper |
| Pockoy 2 | 38CH2533 | UGAMS_53615 | 110H-S-459; Bag 2048 | nut shell (Carya) | -23.2 | 3830 | 25 | This paper |
| Pockoy 2 | 38CH2533 | UGAMS_53616 | 110G-S-457; Bag 4047 | nut shell (Juglans) | -25.7 | 3780 | 25 | This paper |
| Pockoy 1 | 38CH2533 | AA-113823 | 4D; Bag 103 | nut shell (Carya) | -25.4 | 3813 | 45 | This paper |
| Pockoy 1 | 38CH2533 | OxA-40302 | 4D; Bag 103 | nut shell (Carya) | -25.6 | 3851 | 20 | This paper |
| Pockoy 1 | 38CH2533 | UGAMS_51560a | 4D; Bag 103 | nut shell (Carya) | Not reported | 3840 | 24 | This paper |
| Pockoy 1 | 38CH2533 | UGAMS_51560b | 4D; Bag 103 | nut shell (Carya) | Not reported | 3810 | 23 | This paper |
| Pockoy 1 | 38CH2533 | UGAMS_51561a | 23F; Bag 293 | nut shell (Carya) | Not reported | 3830 | 23 | This paper |
| Pockoy 1 | 38CH2533 | UGAMS_51561b | 23F; Bag 293 | nut shell (Carya) | Not reported | 3890 | 23 | This paper |
| Pockoy 1 | 38CH2533 | AA-113824 | 23F; Bag 293 | nut shell (Carya) | -26.9 | 3880 | 45 | This paper |
| Pockoy 1 | 38CH2533 | UGAMS_51562a | 23E; Bag 267 | nut shell (Carya) | Not reported | 3850 | 23 | This paper |
| Pockoy 1 | 38CH2533 | UGAMS_51562b | 23E; Bag 267 | nut shell (Carya) | Not reported | 3830 | 23 | This paper |
| Pockoy 1 | 38CH2533 | AA-113825 | 23E; Bag 267 | nut shell (Carya) | -25.4 | 3812 | 45 | This paper |
| Pockoy 1 | 38CH2533 | OxA-40301 | 23E; Bag 267 | nut shell (Carya) | -25.82 | 3872 | 20 | This paper |
| Pockoy 1 | 38CH2533 | UGAMS_51563a | 24H; Bag 332 | nut shell (Carya) | Not reported | 3880 | 23 | This paper |
| Pockoy 1 | 38CH2533 | UGAMS_51563b | 24H; Bag 332 | nut shell (Carya) | Not reported | 3880 | 23 | This paper |
| Pockoy 1 | 38CH2533 | AA-113826 | 24H; Bag 332 | nut shell (Carya) | -26.7 | 3858 | 45 | This paper |
| Pockoy 1 | 38CH2533 | UGAMS_52720 | 6D; Bag 100 | nut shell (Carya) | -26.2 | 3850 | 23 | This paper |
| Pockoy 1 | 38CH2533 | UGAMS_53610 | 25H-S-18 (LF); Bag 308 | nut shell (Juglans) | -24.5 | 3790 | 25 | This paper |
| Pockoy 1 | 38CH2533 | UGAMS_53611 | 25I-S-21 (HF); Bag 322 | nut shell (Carya) | -25.3 | 3820 | 25 | This paper |
| Pockoy 1 | 38CH2533 | UGAMS-41243r | 3D; Bag 95 | nut shell (Carya) | -25.3 | 3810 | 25 | This paper |
| Pockoy 1 | 38CH2533 | UGAMS_59253 | 29G-S-50 (HF) | nut shell (Carya) | -26.44 | 3800 | 25 | This paper |
| Pockoy 1 | 38CH2533 | UGAMS_41244r | 38CH2533-8E; Bag 163 | nut shell (Carya) | -24.6 | 3850 | 25 | This paper |
| Pockoy 1 | 38CH2533 | UGAMS_41245r | 38CH2533-9H; Bag 359 | nut shell (Carya) | -27.8 | 3880 | 25 | This paper |
| Pockoy 1 | 38CH2533 | AA-110472 | N2040E1150; Bag 26 | deer bone | -22.2 | 3936 | 33 | This paper |
| Pockoy 1 | 38CH2533 | AA-110476 | N2050E1170; Bag 25 | deer bone | -22.8 | 3896 | 33 | This paper |
| Pockoy 1 | 38CH2533 | AA-110471 | N2060E1140; Bag 40 | deer bone | -23.3 | 3881 | 30 | This paper |
| Pockoy 1 | 38CH2533 | AA-110475 | Unit N2059E1160, Lvl 4; Bag 4 | deer bone | -23.7 | 3863 | 33 | This paper |
| Pockoy 1 | 38CH2533 | AA-110474 | Unit N2059E1160, Lvl 7; Bag 7 | deer bone | -24.2 | 3912 | 33 | This paper |
| Pockoy 1 | 38CH2533 | AA-110473 | N2060E1170; Bag 8 | deer bone | -24.4 | 3892 | 33 | This paper |
| Fig 3 | 38CH42 | UGAMS_52711 | Unit 2, Level 9, Fea. 2 | nut shell (Carya) | -26.5 | 3740 | 23 | This paper |
| Fig 3 | 38CH42 | UGAMS_52712 | Unit 2, Level 13 | nut shell (Carya) | -23 | 3750 | 17 | This paper |
| Fig 3 | 38CH42 | UGAMS_52713 | Unit 5, Level 3 | nut shell (Carya) | -27.5 | 3780 | 17 | This paper |
| Fig 2 | 38CH42 | UGAMS_53617a | 201D-S-4 | nut shell (Carya) | -27 | 3660 | 25 | This paper |
| Fig 2 | 38CH42 | UGAMS_53617b | 201D-S-4 | nut shell (Carya) | -27 | 3690 | 30 | This paper |
| Fig 2 | 38CH42 | UGAMS_53618a | 201E-S-7 | nut shell (Carya) | -26 | 3660 | 25 | This paper |
| Fig 2 | 38CH42 | UGAMS_53618b | 201E-S-7 | nut shell (Carya) | -26 | 3690 | 25 | This paper |
| Fig 2 | 38CH42 | UGAMS_52719 | ST 4, Level 12 | nut shell (Carya) | -27.3 | 3750 | 23 | This paper |
| Fig 1 | 38CH42 | UGAMS_53619a | Unit 1, Level 4, Area 1, Strat 1 | nut shell (Carya) | -26.6 | 3510 | 25 | This paper |
| Fig 1 | 38CH42 | UGAMS_53619b | Unit 1, Level 4, Area 1, Strat 1 | nut shell (Carya) | -26.6 | 3520 | 25 | This paper |
| Fig 1 | 38CH42 | UGAMS_52718 | Unit 1, Level 6 | nut shell (Carya) | -26.5 | 825 | 15 | This paper |
| Fig 1 | 38CH42 | UGAMS_52714 | Unit 2, Level 7 | nut shell (Carya) | -28.4 | 3605 | 17 | This paper |
| Fig 1 | 38CH42 | UGAMS_52715 | Unit 2, Level 8 | nut shell (Carya) | -27.6 | 3575 | 17 | This paper |
| Fig 1 | 38CH42 | UGAMS_52716 | Unit 2, Level 10, Strat 3 | nut shell (Carya) | -24.1 | 3589 | 17 | This paper |
| Fig 1 | 38CH42 | UGAMS_52717 | Unit 2, Level 10, Strat 4 | nut shell (Carya) | -27.5 | 3590 | 17 | This paper |
| Spanish Mount | 38CH62 | OxA - 41058 | 16I-S-67; Bag 1073 | nut shell (Carya) | -25.8 | 3679 | 18 | This paper |
| Spanish Mount | 38CH62 | OxA - 41059 | 16I-S-67; Bag 1073 | nut shell (Carya) | -25.9 | 3657 | 18 | This paper |
| Spanish Mount | 38CH62 | UGAMS-59261 | 6X; Bag 294 | wood charcoal | -24.77 | 5040 | 25 | This paper |
| Spanish Mount | 38CH62 | UGAMS-59262 | 7O; Bag 334 | nut shell (Carya) | -25.42 | 3620 | 25 | This paper |
| Spanish Mount | 38CH62 | UGAMS-59263 | 7Q; Bag 338 | wood charcoal | -25.12 | 3670 | 25 | This paper |
| Spanish Mount | 38CH62 | OxA - 41060 | 9G-S-40; Bag 637 | nut shell (Carya) | -25.9 | 3645 | 18 | This paper |
| Spanish Mount | 38CH62 | Beta - 459730 | 6S | pottery residue | -23.8 | 3790 | 30 | This paper |
| Spanish Mount | 38CH62 | Beta - 459728 | 6N-S-14 | wood charcoal | -25.2 | 3500 | 30 | This paper |
| Spanish Mount | 38CH62 | Beta - 489680 | U6O/Q | nut shell (Carya) | -24.5 | 3570 | 30 | This paper |
| Spanish Mount | 38CH62 | Beta - 517656 | 9G-S-40 | nut shell (Carya) | -23.8 | 3620 | 30 | This paper |
| Spanish Mount | 38CH62 | Beta - 517655 | 16I-S-67 | nut shell (Carya) | -24.8 | 3620 | 30 | This paper |
| Spanish Mount | 38CH62 | Beta - 517654 | 12H-S-57 | nut shell (Carya) | -24.1 | 3640 | 30 | This paper |
| Spanish Mount | 38CH62 | Beta - 517653 | 10H-S-46 | nut shell (Carya) | -21.8 | 3580 | 30 | This paper |
| Spanish Mount | 38CH62 | Beta - 489681 | U7-S-26-60cmbs | wood charcoal | -27.4 | 3580 | 30 | This paper |
| Spanish Mount | 38CH62 | Beta - 465411 | 7KS2-128 | wood charcoal | -24.4 | 3590 | 30 | This paper |
| Spanish Mount | 38CH62 | Beta - 465413 | 7KS5-129 | wood charcoal | -26.7 | 3600 | 30 | This paper |
| Spanish Mount | 38CH62 | Beta - 465417 | 8JS15-394 | wood charcoal | -23.9 | 3640 | 30 | This paper |
| Odingsell | 9CH111 | UGAMS_39523 | 9CH111_6-12BS | collagen | -22.2 | 3510 | 30 | This paper |
| Odingsell | 9CH111 | UGAMS_39524 | 9CH111_24-30BS | charcoal | -26.29 | 3890 | 20 | This paper |
| Odingsell | 9CH111 | UGAMS_39525 | 9CH111_34BS | charcoal | -25.77 | 3860 | 20 | This paper |
| Bluff Field | 9CH160 | UGAMS_59905 | 9CH160_F1_LVL2_S1 | collagen | -21.91 | 4290 | 25 | Garland et al. (under review) |
| Bluff Field | 9CH160 | UGAMS_59906 | 9CH160_F1_LVL4_S1 | collagen | -22.79 | 4300 | 25 | Garland et al. (under review) |
| Bluff Field | 9CH160 | UGAMS_59907 | 9CH160_F1_LVL4_S2 | nutshell | -27.65 | 4430 | 25 | Garland et al. (under review) |
| Bluff Field | 9CH160 | UGAMS_59908 | 9CH160_F1_LVL5_S2 | nutshell | -26.11 | 4410 | 25 | Garland et al. (under review) |
| Bluff Field | 9CH160 | UGAMS_59909 | 9CH160_C1_LVL3_S1 | nutshell | -26.73 | 4370 | 25 | Garland et al. (under review) |
| Bluff Field | 9CH160 | UGAMS_59910 | 9CH160_C1_LVL3_S2 | collagen | -22.51 | 4390 | 25 | Garland et al. (under review) |
| Bluff Field | 9CH160 | UGAMS_59911 | 9CH160_C1_LVL4_S1 | nutshell | -25.24 | 4420 | 25 | Garland et al. (under review) |
| Bluff Field | 9CH160 | UGAMS_59916 | 9CH160_D1_LVL3_S1 | collagen | -22.32 | 4330 | 25 | Garland et al. (under review) |
| Bluff Field | 9CH160 | UGAMS_59917 | 9CH160_D1_LVL4_S1 | collagen | -22.35 | 4390 | 25 | Garland et al. (under review) |
| Bluff Field | 9CH160 | UGAMS_59920 | 9CH160_E1_LVL2_S2 | collagen | -22.13 | 4190 | 25 | Garland et al. (under review) |
| Bluff Field | 9CH160 | UGAMS_59921 | 9CH160_E1_LVL3_S1 | nutshell | -25.40 | 4390 | 25 | Garland et al. (under review) |
| Bluff Field | 9CH160 | UGAMS_59922 | 9CH160_E1_LVL4_S1 | nutshell | -27.39 | 4410 | 20 | Garland et al. (under review) |
| Ossabaw Shell Ring | 9CH203 | UGAMS_26042 | 9CH203A1-3 | charcoal | -25.8 | 3730 | 30 | Lulewicz et al. 2017 |
| Ossabaw Shell Ring | 9CH203 | UGAMS_26043 | 9CH203A1-4 | collagen | -22.8 | 3660 | 30 | Lulewicz et al. 2017 |
| Ossabaw Shell Ring | 9CH203 | UGAMS_26044 | 9CH203A1-5 | collagen | -21.8 | 3720 | 30 | Lulewicz et al. 2017 |
| Ossabaw Shell Ring | 9CH203 | UGAMS_26045 | 9CH203A1-6 | charcoal | -25.9 | 3830 | 30 | Lulewicz et al. 2017 |
| Cane Patch | 9CH35 | RC2A | 80 CMBD – 41 cm East of West Wall in North Profile | Charcoal | -24.8 | 3710 | 25 | This paper |
| Cane Patch | 9CH35 | RC2C | 76 CMBD – 125 West of East Wall | Charcoal | -24.7 | 3720 | 25 | This paper |
| Cane Patch | 9CH35 | RC6A | 43 CMBD – 15 cm from South Wall in West Profile | Charcoal | -26.1 | 3570 | 25 | This paper |
| Cane Patch | 9CH35 | RC6B | 70 CMBD – 12 cm from South Wall in West Profile | Charcoal | -25.4 | 3650 | 25 | This paper |
| Cane Patch | 9CH35 | UGAMS-23617 | Unit 4, Level 4, Feature 69 | Charcoal | -25.7 | 3560 | 25 | This paper |
| Cane Patch | 9CH35 | UGAMS-23618 | Unit 4, Level 9, Feature 117 | Charcoal | -27.4 | 3670 | 25 | This paper |
| Cane Patch | 9CH35 | UGAMS-23619 | Unit 5, Level 5, Feature 145 | Charcoal | -27 | 3760 | 25 | This paper |
| Cane Patch | 9CH35 | UGAMS-23620 | Unit 5, Level 6, Feature 152 | Charcoal | -26.5 | 3660 | 25 | This paper |
| Cane Patch | 9CH35 | UGAMS-23621 | Unit 5, Level 8, Feature 164 | Charcoal | -26.6 | 3800 | 25 | This paper |
| Bilbo | 9CH4 | M-1109 | Bilbo: 3.0-3.5 ft. bs (Zone 3, Waring) | Wood | -25 | 3700 | 125 | Sassaman 1996 (Sassaman 1993a) |
| Bilbo | 9CH4 | M-1111 | Bilbo: 5.5-6.0 ft. bs (Zone 1, Waring) | Wood | -25 | 3820 | 125 | Sassaman 1996 (Sassaman 1993a) |
| Bilbo | 9CH4 | O-1046 | Complex lenses of shell midden and alluvial sand 2.0 to 2.5 feet bs, Haag Zones 7-8/ Waring Zone 3 (3-3.5 feet above msl) | Charcoal | -25 | 5550 | 115 | Sassaman 1996 (Sassaman 1993a) |
| Bilbo | 9CH4 | O-1047 | Nearly solid oyster midden, 5.5 to 6.0 feet bs, Haag Zone 4 (0-0.5 ft below msl) | Charcoal | -25 | 4125 | 115 | Sassaman 1996 (Sassaman 1993a) |
| Bilbo | 9CH4 | UGA-10676 | Test Pit #14- Upper limit of undisturbed shell midden, 70-80cm below datum | Charcoal | -25 | 3650 | 50 | Crook 2009 |
| Bilbo | 9CH4 | UGA-10677 | Test Pit #14- Base of dense shell deposit, 170-180cm below datum | Charcoal | -25 | 3770 | 140 | Crook 2009 |
| Bilbo | 9CH4 | UGA-10678 | Test Pit #14- Underneath the shell deposit, 190-200cm below datum | Charcoal | -25 | 3720 | 90 | Crook 2009 |
| Pagan Plum | 9CH61 | UGAMS_39510 | 9CH61_P1LVL1_0-0.5 | collagen | -22.62 | 3400 | 20 | This paper |
| Pagan Plum | 9CH61 | UGAMS_39511 | 9CH61_P1LVL4_2.1 | charcoal | -26.25 | 3790 | 20 | This paper |
| Pagan Plum | 9CH61 | UGAMS_39512 | 9CH61_P1LVL5_1.5-2.0 | collagen | -22.67 | 3870 | 20 | This paper |
| Pagan Plum | 9CH61 | UGAMS_39513 | 9CH61_P1LVL5_2.5 | charcoal | -25.97 | 3890 | 20 | This paper |
| Pagan Plum | 9CH61 | UGAMS_39514 | 9CH61_P1LVL6_2.5-3.0 | collagen | -22.51 | 3870 | 20 | This paper |
| McQueen Shell Ring | 9LI1648 | Beta-244620 | Feature 21, 4.0-3.9 m | Charcoal | -25.3 | 3800 | 40 | This paper |
| McQueen Shell Ring | 9LI1648 | Beta-251761 | N243 E233, 4.5-4.4 m | Charcoal | -23.9 | 3700 | 40 | This paper |
| McQueen Shell Ring | 9LI1648 | Beta-251764 | N272 E200, 5.3-5.2 m | Charcoal | -25 | 3710 | 40 | This paper |
| McQueen Shell Ring | 9LI1648 | Beta-251766 | N272 E200, 5.1-5.0 m | Charcoal | -27.5 | 3840 | 40 | This paper |
| McQueen Shell Ring | 9LI1648 | Beta-251767 | N243 E233, 4.4-4.3 m | carbonized wood | -24.8 | 3680 | 40 | This paper |
| McQueen Shell Ring | 9LI1648 | UCIAMS-84269 | N243 E234, 4.46-4.35m | Charcoal |  | 3620 | 20 | This paper |
| McQueen Shell Ring | 9LI1648 | UCIAMS-84270 | N243 E234 4.4-4.35m | Charcoal |  | 3640 | 20 | This paper |
| McQueen Shell Ring | 9LI1648 | UCIAMS-87903 | N243 E234, 4.46-4.35m | deer bone | -22 | 3685 | 20 | This paper |
| McQueen Shell Ring | 9LI1648 | UCIAMS-87904 | N243 E233 4.4-4.3m | deer bone | -21.1 | 3730 | 30 | This paper |
| McQueen Shell Ring | 9LI1648 | UCIAMS-87905 | N243 E235 4.4-4.36m | deer bone | -21.3 | 3690 | 20 | This paper |
| Musgrove Shell Ring | 9LI2169 | UGAMS 65147 | near top of shell deposit, N2093_E4176 | carbonized wood | -26.61 | 3820 | 25 | This paper |
| Musgrove Shell Ring | 9LI2169 | UGAMS 65148 | near bottom of shell deposit, N2093_E4176 | hickory nut (Carya spp) | -24.96 | 3800 | 25 | This paper |
| Musgrove Shell Ring | 9LI2169 | UGAMS 65149 | hickory nut pulled from ABOT sample; dark soil under shell, N2093_E4176 | hickory nut (Carya spp) | -25.88 | 3840 | 25 | This paper |
| Musgrove Shell Ring | 9LI2169 | UGAMS 65150 | Feature 5 - shell filled pit at bottom shell deposit, N2093_E4176 | carbonized wood | -25.31 | 3860 | 25 | This paper |
| Musgrove Shell Ring | 9LI2169 | UGAMS 65151 | top of shell deposit, N2121_E4209 | carbonized wood | -25.46 | 3900 | 25 | This paper |
| Musgrove Shell Ring | 9LI2169 | UGAMS 65152 | bottom of shell deposit, N2121_E4209 | carbonized wood | -28.19 | 3910 | 25 | This paper |
| Musgrove Shell Ring | 9LI2169 | UGAMS 65153 | dark soil under shell deposit, N2121_E4209 | hickory nut (Carya spp) | -26.51 | 3810 | 25 | This paper |
| Musgrove Shell Ring | 9LI2169 | UGAMS 65155 | Feature 9 - in situ burning event under shell, N2121_E4209 | carbonized wood | -26.82 | 3910 | 25 | This paper |
| Musgrove Shell Ring | 9LI2169 | UGAMS 65156 | near top of shell deposit, N2131_E4186 | carbonized wood | -26.84 | 3820 | 25 | This paper |
| Musgrove Shell Ring | 9LI2169 | UGAMS 65157 | bottom of shell deposit, N2131_E4186 | carbonized wood | -26.10 | 3840 | 25 | This paper |
| Musgrove Shell Ring | 9LI2169 | UGAMS 65158 | dark soil under shell, N2131_E4186 | carbonized wood | -26.96 | 3950 | 25 | This paper |
| Musgrove Shell Ring | 9LI2169 | UGAMS 65159 | Feature 1 - in situ burning event within shell deposit, N2131_E4186 | carbonized wood | -26.41 | 3860 | 25 | This paper |
| Musgrove Shell Ring | 9LI2169 | UGAMS 65160 | Feature 2 - dark organic soil under Feature 1 within shell  deposit, N2131_E4186 | carbonized wood | -26.60 | 3860 | 25 | This paper |
| Musgrove Shell Ring | 9LI2169 | UGAMS 65161 | Feature 7 - dark soil with some shell under Feature 2, within shell deposit, N2131_E4186 | carbonized wood | -25.44 | 4060 | 25 | This paper |
| St Catherines Shell Ring | 9LI231 | Beta-238322 | Feature 60, 2.0-1.9 m | hickory nut (Carya spp) | -25.7 | 3880 | 40 | This paper |
| St Catherines Shell Ring | 9LI231 | Beta-238327 | W92 S2, 2.3-2.2 m | hickory nut (Carya spp) | -24.2 | 3810 | 40 | This paper |
| St Catherines Shell Ring | 9LI231 | Beta-238328 | Feature 76, 1.9-1.8 m | carbonized wood | -24.5 | 4110 | 40 | This paper |
| St Catherines Shell Ring | 9LI231 | Beta-238331 | Feature 88, 1.8-1.7 m | carbonized wood | -25.4 | 3830 | 40 | This paper |
| St Catherines Shell Ring | 9LI231 | Beta-238332 | Feature 73, 1.8-1.7 m | carbonized wood | -26 | 3900 | 40 | This paper |
| St Catherines Shell Ring | 9LI231 | Beta-238337 | N771 E819, 2.39-2.3 | carbonized wood | -26.8 | 3890 | 40 | This paper |
| St Catherines Shell Ring | 9LI231 | UGAMS 60773 | Level 10-20 cm, N789_E801 | deer bone | -21.99 | 3730 | 25 | This paper |
| St Catherines Shell Ring | 9LI231 | UGAMS 60774 | Level 30-40 cm, N789_E801 | deer bone | -22.50 | 3820 | 20 | This paper |
| St Catherines Shell Ring | 9LI231 | UGAMS 60775 | Level 40-50 cm, N789_E801 | deer bone | -23.00 | 3820 | 20 | This paper |
| St Catherines Shell Ring | 9LI231 | UGAMS 60776 | Level 50-60 cm, N789_E801 | deer bone | -22.46 | 3850 | 20 | This paper |
| Sapelo Shell Ring II | 9MC23 | UGAMS_1424 | Ring II: Unit 1 2006, NW Quad, Level 5 | Charcoal | -26.16 | 3750 | 70 | Garland et al. 2022 |
| Sapelo Shell Ring II | 9MC23 | UGAMS_1425 | Ring II: Unit 1 2006, NE Quad, Level 5 | Pottery Residue | -25.87 | 3890 | 60 | Garland et al. 2022 |
| Sapelo Shell Ring III | 9MC23 | UGAMS_15082 | Unit 9, Level 4 | charcoal | -27.5 | 3560 | 50 | Garland et al. 2022 |
| Sapelo Shell Ring III | 9MC23 | UGAMS_15083 | Unit 9, Level 7 | charcoal | -25.5 | 3730 | 60 | Garland et al. 2022 |
| Sapelo Shell Ring I | 9MC23 | UGAMS_15084 | Unit 1, Level 2, 10-20 cmbs | sooted sherd | -17.0 | 3610 | 50 | Garland et al. 2022 |
| Sapelo Shell Ring I | 9MC23 | UGAMS_15085 | Unit 1, Level 2, 10-20 cmbs | sooted sherd | -18.9 | 3730 | 60 | Garland et al. 2022 |
| Sapelo Shell Ring III | 9MC23 | UGAMS_15086 | Unit 11, Level 4 | charcoal | -25.6 | 3730 | 50 | Garland et al. 2022 |
| Sapelo Shell Ring II | 9MC23 | UGAMS_42750 | 9MC23 Ring II_Unit 1_37.5 | charred pine | -25.75 | 3800 | 20 | Garland et al. 2022 |
| Sapelo Shell Ring II | 9MC23 | UGAMS_42751 | 9MC23 Ring II_Unit 1_47 | charred pine | -26.97 | 3770 | 20 | Garland et al. 2022 |
| Sapelo Shell Ring II | 9MC23 | UGAMS_42752 | 9MC23 Ring II_Unit 1_70-75 | charred pine | -26.09 | 3810 | 20 | Garland et al. 2022 |
| Sapelo Shell Ring III | 9MC23 | UGAMS_52174 | 9MC23U9LVL5 | plant frag | -26.84 | 3770 | 20 | Garland et al. 2022 |
| Sapelo Shell Ring II | 9MC23 | UGAMS_52175 | 9MC23A1LVL3 | plant frag | -26.33 | 3680 | 20 | Garland et al. 2022 |
| Sapelo Shell Ring III | 9MC23 | UGAMS_52177 | 9MC23U9LVL2 | charcoal | -25.23 | 3590 | 20 | Garland et al. 2022 |
| Sapelo Shell Ring III | 9MC23 | UGAMS_52178 | 9MC23U9LVL3 | plant frag | -25.09 | 3620 | 20 | Garland et al. 2022 |
| Sapelo Shell Ring III | 9MC23 | UGAMS_52179 | 9MC23U9LVL6 | charcoal | -26.29 | 3660 | 20 | Garland et al. 2022 |
| Sapelo Shell Ring III | 9MC23 | UGAMS_52180 | 9MC23U9LVL8 | plant frag | -25.13 | 3690 | 20 | Garland et al. 2022 |
| Sapelo Shell Ring III | 9MC23 | UGAMS_52181 | 9MC23U9LVL9 | charcoal | -26.59 | 3570 | 20 | Garland et al. 2022 |
| Sapelo Shell Ring I | 9MC23 | UGAMS_52182 | 9MC23U4LVL7 | charcoal | -25.9 | 3750 | 20 | Garland et al. 2022 |
| Sapelo Shell Ring I | 9MC23 | UGAMS_52183 | 9MC23U4LVL9 | collagen | -22.89 | 3780 | 20 | Garland et al. 2022 |
| Sapelo Shell Ring I | 9MC23 | UGAMS_52184 | 9MC23U4LVL10S1 | collagen | -22.67 | 3670 | 20 | Garland et al. 2022 |
| Sapelo Shell Ring I | 9MC23 | UGAMS_52185 | 9MC23U4LVL10S2 | collagen | -21.29 | 3830 | 20 | Garland et al. 2022 |
| Sapelo Shell Ring I | 9MC23 | UGAMS_52186 | 9MC23U4LVL11S1 | plant frag | -26.73 | 3840 | 20 | Garland et al. 2022 |
| Sapelo Shell Ring I | 9MC23 | UGAMS_52187 | 9MC23U4LVL11S2 | collagen | -22.43 | 3810 | 20 | Garland et al. 2022 |
| Sapelo Shell Ring I | 9MC23 | UGAMS_52188 | 9MC23U4LVL13S1 | plant frag | -25.74 | 3820 | 20 | Garland et al. 2022 |
| Sapelo Shell Ring I | 9MC23 | UGAMS_52189 | 9MC23U4LVL13S2 | collagen | -22.62 | 3820 | 20 | Garland et al. 2022 |
| Sapelo Shell Ring I | 9MC23 | UGAMS_52190 | 9MC23U4LVL14 | plant frag | -25.44 | 3810 | 20 | Garland et al. 2022 |
| Sapelo Shell Ring I | 9MC23 | UGAMS_52191 | 9MC23U4LVL17 | collagen | -22.8 | 3790 | 20 | Garland et al. 2022 |
| Creighton Shell Ring | 9MC87 | UGAMS_39515 | 9MC87_P1LVL4_0.9-1.2 | collagen | -22.57 | 3650 | 20 | Garland et al. 2022 |
| Creighton Shell Ring | 9MC87 | UGAMS_39516 | 9MC87_P1LVL8_2.1-2.4 | charcoal | -26.02 | 3710 | 20 | Garland et al. 2022 |
| Creighton Shell Ring | 9MC87 | UGAMS_39517 | 9MC87_P1LVL12_3.3-3.6 | collagen | -22.65 | 3810 | 20 | Garland et al. 2022 |
| Creighton Shell Ring | 9MC87 | UGAMS_39518 | 9MC87_P1LVL14_4.1 | charcoal | -26.03 | 3770 | 20 | Garland et al. 2022 |
| Creighton Shell Ring | 9MC87 | UGAMS_39519 | 9MC87_P1LVL16_4.5-4.8 | collagen | -23.68 | 2760 | 35 | Garland et al. 2022 |
| Creighton Shell Ring | 9MC87 | UGAMS_39520 | 9MC87_P1LVL16_4.8 | charcoal | -26.27 | 3750 | 20 | Garland et al. 2022 |
| Creighton Shell Ring | 9MC87 | UGAMS_39521 | 9MC87_P1Z3_5.3 | charcoal | -25.11 | 3800 | 20 | Garland et al. 2022 |
| Creighton Shell Ring | 9MC87 | UGAMS_39522 | 9MC87_P1LVL20_5.9 | charcoal | -24.77 | 3900 | 20 | Garland et al. 2022 |

***The Musgrove Shell Ring (9LI2169).***

Four samples come from pre-ring deposits, two samples come from the uppermost levels of one excavation, and the remaining 12 are from levels demonstratively associated with shell deposition as part of the ring itself (Table S2). For the dates associated with shell deposition we constructed six different models as a sensitivity analysis for our dating project. We report these three models here for clarity and transparency of the Bayesian modeling process. All modeled dates are provided in italics and the structure of the models can be observed from the bracketed structure of the probability distribution plots in addition to the runfiles provided in the supplemental materials.

Model 1 places all the dates from the test units into one Phase that contains several ordered Sequences. The order of the dates within each Sequence is the stratigraphic order that the excavators recovered each sample that include both shell dense layers and non-shell layers under the ring. UGAMA 65154 which comes from the base of the excavation is an anomalous old date not associated with the shell ring formation and is not included in the model, nor any of the other subsequence models presented here. The results of Model 1 indicate poor agreement. The Amodel (1) indicates poor agreement between parameters and the dates, i.e., not exceeding the 60-threshold (Table S3). Therefore, it appears that there is mixing of the samples which is likely due to the inclusion of dates derived from features that cross-cut levels.

Model 2 places all the dates from the test units into one Phase that contains several ordered Sequences. However, unlike Model 1 while the order of the dates within each Sequence is the stratigraphic order, only those samples that come from dense layers within the ring are structured this way. The results of Model 2 indicate poor agreement. The Amodel (2.7) for the model do not indicate good agreement, i.e., exceeding the 60-threshold (see Table S4). Therefore, it appears that there is mixing of the samples which like Model 1 is likely due to the inclusion of dates derived from features that cross-cut levels.

Model 3 places all the dates from the test units into one Phase that contains two ordered Sequences, samples from Unit N2093 E4176 and those from Unit N2221 E4209. For Unit N2131 E4186, however, these samples were placed in an unordered Phase given that features crosscut much of the stratigraphy in this unit. The results of Model 3 indicate good agreement. The Amodel (82.5) for the model indicates good agreement, i.e., exceeding the 60-threshold (see Table S5). The model estimates a start date for the ring of *4500-4440 cal. BP* (68.3 hpd) and an end date of *4190-4130 cal. BP* (68.3 hpd) and a start date for the ring of *4570-4420 cal. BP* (95.4 hpd) and an end date of *4240-4080 cal. BP* (95.4 hpd).

Model 4 is essentially the same as Model 3; however, for this one we apply a General Outlier model to all the dates The results of Model 4 indicate good agreement. The Amodel (97.2) for the model indicates good agreement, exceeding the 60-threshold (see Table S6). The model estimates a start date for the ring of *4290-4190 cal. BP* (68.3 hpd) and an end date of *4230-4150 cal. BP* (68.3 hpd) and a start date for the ring of *4460-4150cal. BP* (95.4 hpd) and an end date of *4240-4100 cal. BP* (95.4 hpd).

Model 5 is similar to Model 4; however, for this one we apply both a General Outlier model to the hickory nut dates and Charcoal Outlier model to all the UID carbonized wood dates. The results of Model 5 indicate good agreement. The Amodel (81.9) for the model indicates good agreement, exceeding the 60-threshold (see Table S7). The model estimates a start date for the ring of *4500-4430 cal. BP* (68.3 hpd) and an end date of *4200-4120 cal. BP* (68.3 hpd) and a start date for the ring of *4580-… cal. BP* (95.4 hpd) and an end date of *4240-4080 cal. BP* (95.4 hpd).

Model 6 is identical to Model 5 in structure except that we use the KDE command in Oxcal to provide a summary of the probability distribution (see Manning et al. 2022:799). Following Manning et al. (2022) we employ a LnN(ln(125), ln (2) for the shape of the probability distribution, which places emphasis on the earlier part of the distribution rather than later (i.e., < 125 years). The prior of 125 years is based on the observation that the span of many of the ring villages tend to be no longer than 250 years based on high precision dating of shell deposition layers at these sites (Thompson et al. in prep). The results of Model 6 indicate good agreement. The Amodel (102.5) indicates good agreement, exceeding the 60-threshold (see Table S8). The model estimates a start date for the ring of *4290-4210 cal. BP* (68.3 hpd) and an end date of *4220-4140 cal. BP* (68.3 hpd) and a start date for the ring of *4350-4160 cal. BP* (95.4 hpd) and an end date of *4240-4110 cal. BP* (95.4 hpd). The Interval of occupation is estimated to be 0-110 years (68.3 hpd) and 0-190 (95.3 hpd).

Models 1 and 2 do not meet the good agreement for Bayesian analysis (i.e., Amodel is less than 60). Models 3, 4, and 5 all are significant and produce very similar but not identical results. Model 6 presents the most constrained date range. This model too likely represents the best estimate of site occupation based on the currently available data and the dates agree best with the assumptions and priors.

**Table S3**: 9LI2169R1M1


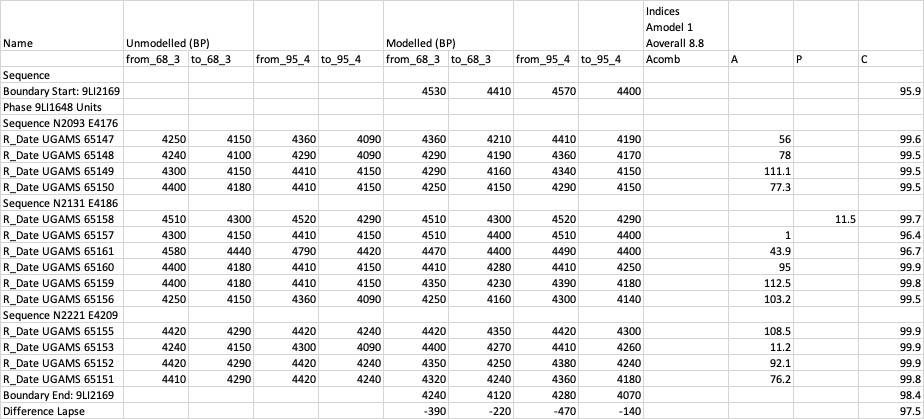


**Table S4**: 9LI2169R1M2


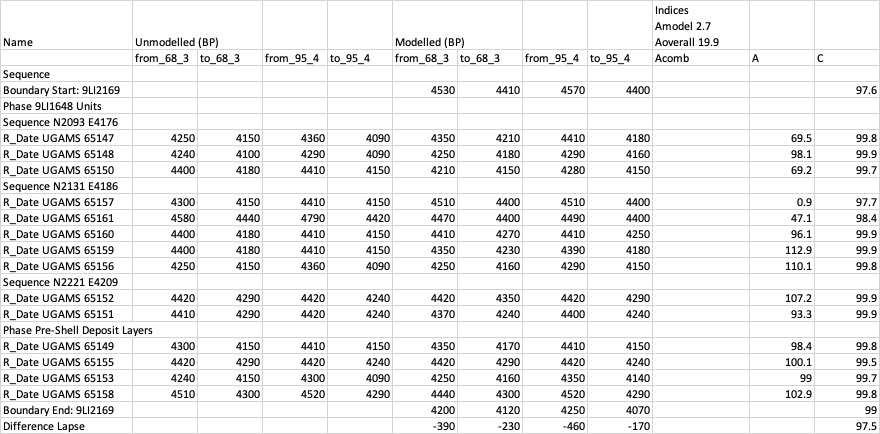


**Table S5**: 9LI2169R1M3


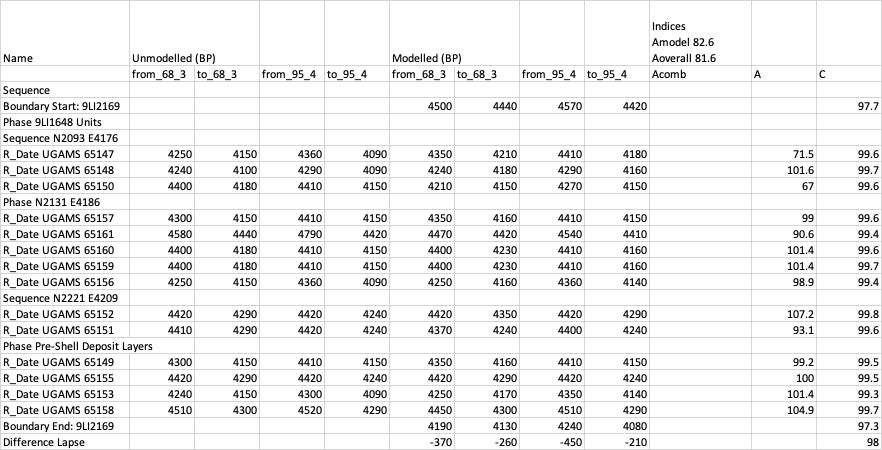


**Table S6**: 9LI2169R1M4


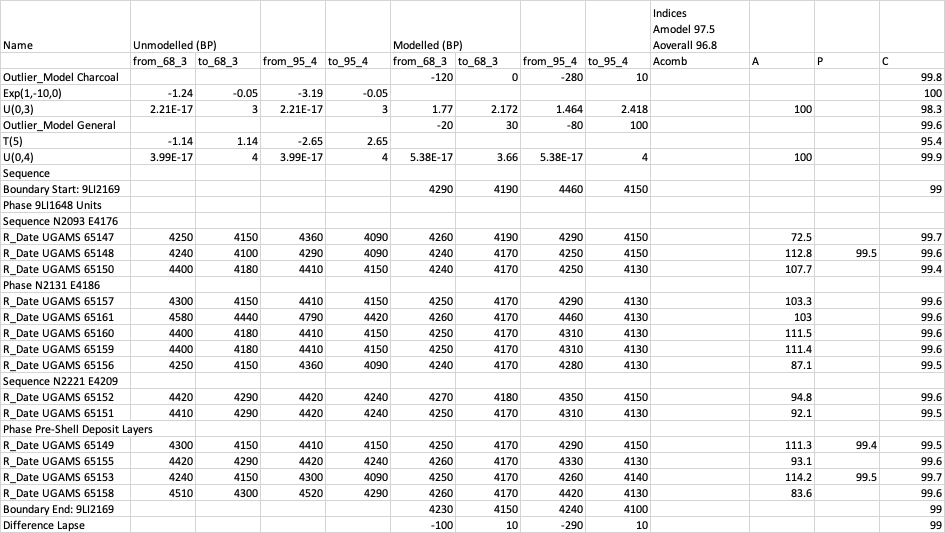


**Table S7**: 9LI2169R1M5


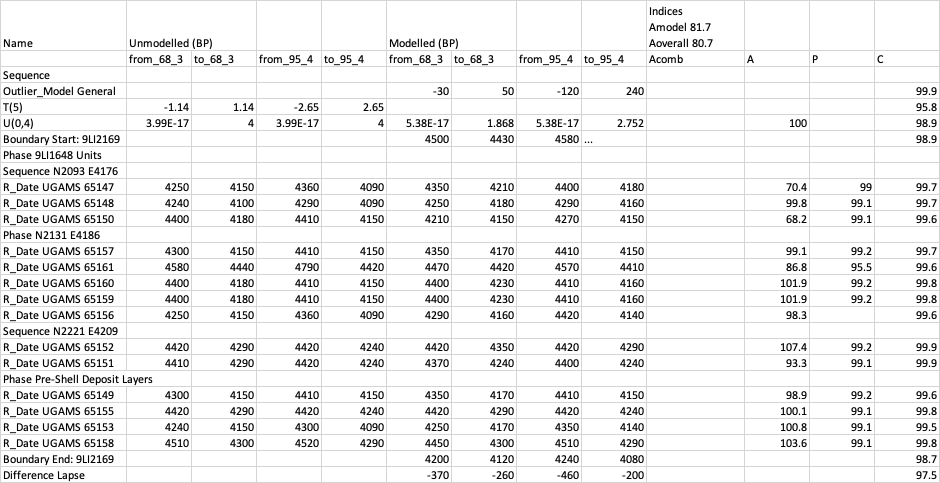


**Table S8**: 9LI2169R1M6


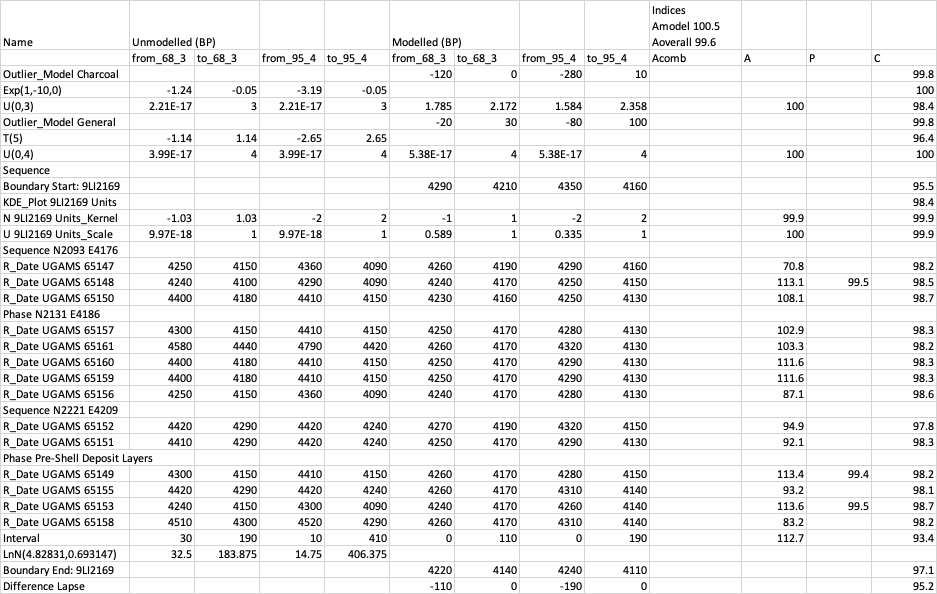


***9LI231 St. Catherines Ring***

Model 1 places all the dates from the test units into one Phase that contains several ordered Sequences. The order of the dates within each Sequence is the stratigraphic order that the excavators recovered each sample that include both shell dense layers and non-shell layers under the ring. The results of Model 1 indicate good agreement. The Amodel (92.4) for the model indicates good agreement, i.e., exceeding the 60-threshold (see Table S9). However, one date, Beta 238328 was identified as and outlier for the model with poor convergence. The model estimates a start date for the ring of *4630-4440 cal. BP* (68.3 hpd) and an end date of *4140-4040 cal. BP* (68.3 hpd) and a start date for the ring of *4770-4420 cal. BP* (95.4 hpd) and an end date of *4150-3900 cal. BP* (95.4 hpd).

Model 2 is identical to Model 1, however, Beta 238328 is manually labeled as an outlier and excluded from the modeled results, as this is an interior plaza date and does not assess ring development. The results of Model 2 indicate good agreement. The Amodel (111.9) for the model indicates good agreement, i.e., exceeding the 60-threshold (see Table S10). The model estimates a start date for the ring of *4370-4240 cal. BP* (68.3 hpd) and an end date of *4150-4080 cal. BP* (68.3 hpd) and a start date for the ring of *4440-4170 cal. BP* (95.4 hpd) and an end date of *4300-4000 cal. BP* (95.4 hpd).

Model 3 is essentially the same as Model 2; however, for this one we apply a General Outlier model to all the dates. Also, the date Beta 238328 was removed from the model completely. The results of Model 3 indicate good agreement. The Amodel (111.9) for the model indicates good agreement, exceeding the 60-threshold (see Table S11). The model estimates a start date for the ring of *4370-4190 cal. BP* (68.3 hpd) and an end date of *4150-4080 cal. BP* (68.3 hpd) and a start date for the ring of *4440-4170 cal. BP* (95.4 hpd) and an end date of *4230-4000 cal. BP* (95.4 hpd).

Model 4 is essentially the same as Model 3; however, for this one we apply a General Outlier model to all the dates. Also, the date Beta 238328 was removed from the model completely. The results of Model 3 indicate good agreement. The Amodel (113.4) for the model indicates good agreement, exceeding the 60-threshold (see Table S12). The model estimates a start date for the ring of *4360-4240 cal. BP* (68.3 hpd) and an end date of *4150-4080 cal. BP* (68.3 hpd) and a start date for the ring of *4440-4170 cal. BP* (95.4 hpd) and an end date of *4230-4000 cal. BP* (95.4 hpd).

Model 5 is essentially the same as Model 4; however, for this one we apply both a General Outlier and a Charcoal Outlier model to all the dates. The results of Model 5 indicate good agreement. The Amodel (94.7) for the model indicates good agreement, exceeding the 60-threshold (see Table S13). The model estimates a start date for the ring of *4370-42400 cal. BP* (68.3 hpd) and an end date of *4150-4070 cal. BP* (68.3 hpd) and a start date for the ring of *4440-4170 cal. BP* (95.4 hpd) and an end date of *4230-3990 cal. BP* (95.4 hpd). The model did identify one date as having poor agreement (UGAMS 60773).

Model 6 is essentially the same as Model 4; however, for this one we only apply the General model to all the dates and an apply a KDE command with a LnN(ln(125), ln (2) to summarize the probability distribution, which places emphasis on the earlier part of the distribution rather than later (i.e., < 125 years) (see above earlier discussion). The results of Model 6 indicate good agreement. The Amodel (101.5) for the model indicates good agreement, exceeding the 60-threshold (see Table S14). The model estimates a start date for the ring of *4320-4190 cal. BP* (68.3 hpd) and an end date of *4150-4090 cal. BP* (68.3 hpd) and a start date for the ring of *4380-4170 cal. BP* (95.4 hpd) and an end date of *4230-4060 cal. BP* (95.4 hpd). The Interval of occupation is estimated to be 40-200 years (68.3 hpd) and 10-280 (95.3 hpd).

All the models exceed the values for good agreement for Bayesian analysis (i.e., Amodel is less than 60). The start and end dates for all the models are similar. Model 6 presents the most constrained date range. This model too likely represents the best estimate of site occupation based on the currently available data and the dates agree best with the assumptions and priors.

**Table S9**: 9LI231R1M1


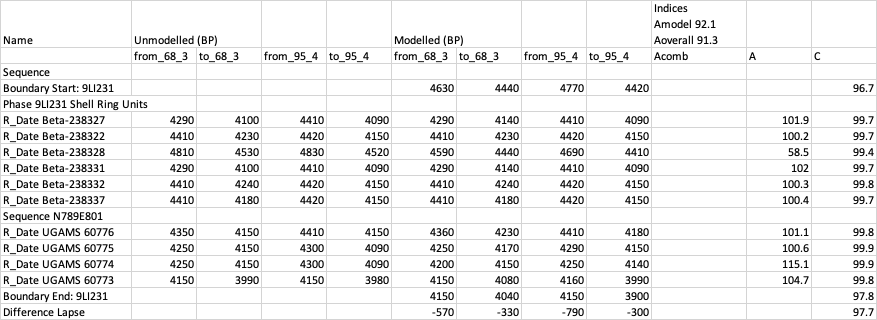


**Table S10**: 9LI231R1M2


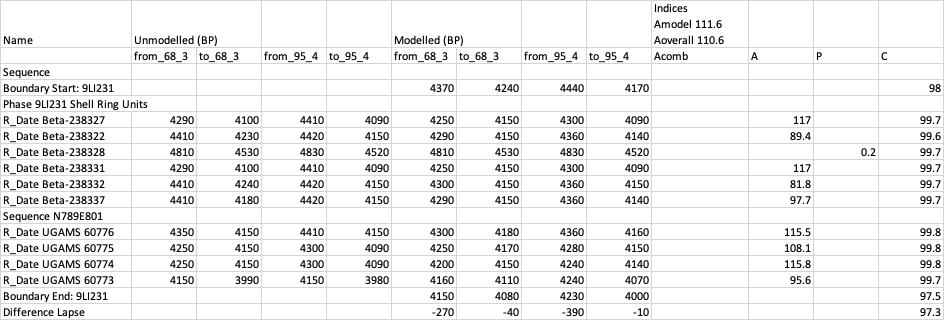


**Table S11**: 9LI231R1M3

**
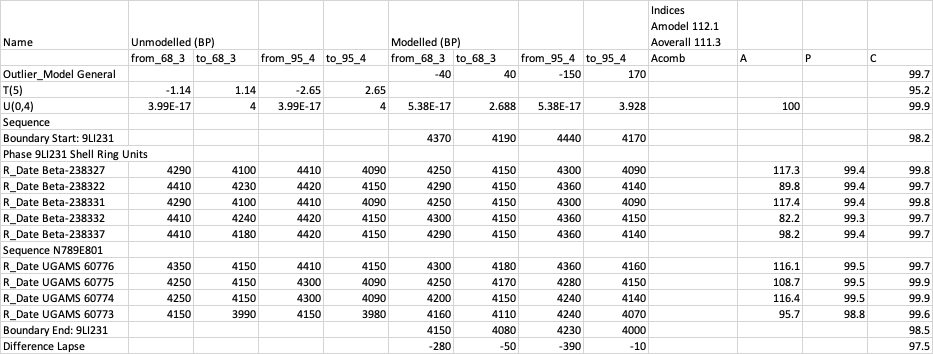
**

**Table S12:** 9LI231R1M4

***
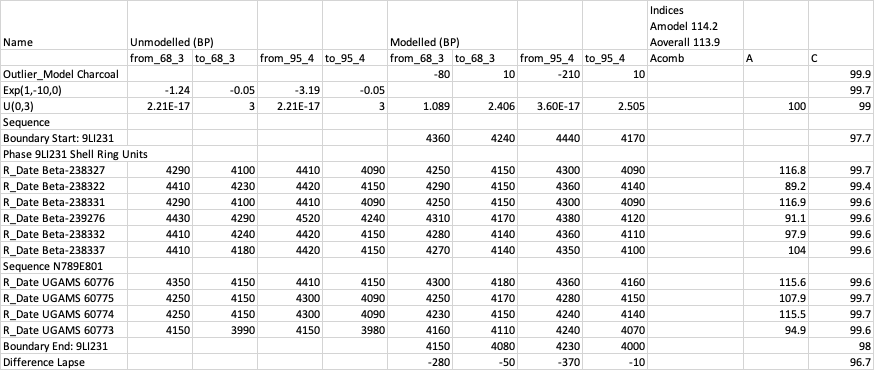
***

**Table S13:** 9LI231R1M5

***
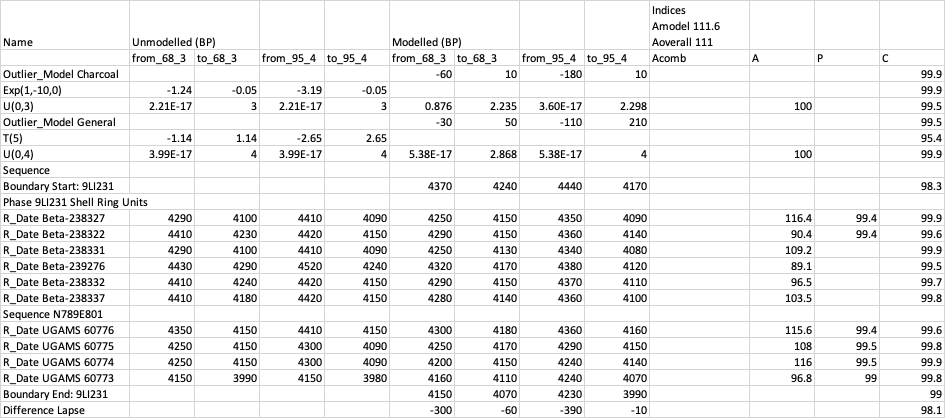
***

**Table S14:** 9LI231R1M6

***
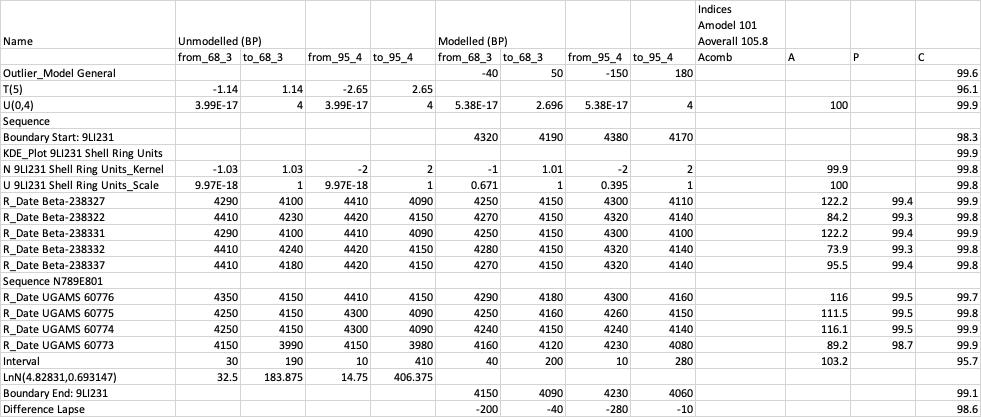
***

***9LI1648 McQueen Ring***

Model 1 places all the dates from the test units into one Phase that contains several ordered Sequences. The order of the dates within each Sequence is the stratigraphic order that the excavators recovered each sample that include both shell dense layers and non-shell layers under the ring. Dates UCIAMS 87905 and Beta 244620 were placed in a single unordered Phase within the larger phase. The results of Model 1 indicate poor agreement. The Amodel (60.9) for the model do indicates good agreement, i.e., exceeding the 60-threshold (see Table S15). At least two dates were identified as outliers for the model (Beta 44620 and Beta 251766). The model estimates a start date for the ring of *4170-4040 cal. BP* (68.3 hpd) and an end date of *3990-3890 cal. BP* (68.3 hpd) and a start date for the ring of *4250-4000 cal. BP* (95.4 hpd) and an end date of *4060-3850 cal. BP* (95.4 hpd).

Model 2 is the same as Model 1, however after discovering that Beta 244620 comes from a pit in the center of the ring and not the ring itself this was manually marked as an Outlier in the model. We also add an Interval command to estimate the duration of occupation. The results of Model 1 indicate good agreement. The Amodel (60.8) for the model indicates good agreement, i.e., exceeding the 60-threshold. The model identifies Beta 251766 as an outlier with poor agreement with the overall model (A=24.3) (see Table S16). The model estimates a start date for the ring of *4120-4020 cal. BP* (68.3 hpd) and an end date of *3990-3890 cal. BP* (68.3 hpd) and a start date for the ring of *4190-3990 cal. BP* (95.4 hpd) and an end date of *4060-3860 cal. BP* (95.4 hpd).

Model 3 is the same as Model 2, however for this one we apply a General Outlier model to all the dates. The results of Model 3 indicate good agreement. The Amodel (64.4) for the model indicates good agreement, i.e., exceeding the 60-threshold. The model identifies Beta 251766 as an outlier with poor agreement with the overall model (A=23.7) (see Table S17). The model estimates a start date for the ring of *4120-4020 cal. BP* (68.3 hpd) and an end date of *4030-3890 cal. BP* (68.3 hpd) and a start date for the ring of *4180-3990 cal. BP* (95.4 hpd) and an end date of *4070-3860 cal. BP* (95.4 hpd).

Model 4 is the same as Model 3, however for this one we apply a Charcoal Outlier model to all the UID charcoal dates. The results of Model 4 indicate good agreement. The Amodel (100.6) for the model indicates good agreement, i.e., exceeding the 60-threshold (see Table S18). Given that Model 3 identified Beta 251766 as an outlier with poor agreement with the overall model (A=23.7) and that this date comes from the very bottom of the unit and may not be associated with rind development we exclude it from the model. The model estimates a start date for the ring of *4100-4010 cal. BP* (68.3 hpd) and an end date of *3980-3880 cal. BP* (68.3 hpd) and a start date for the ring of *4170-3980 cal. BP* (95.4 hpd) and an end date of *4060-3830 cal. BP* (95.4 hpd).

Model 5 is the same as Model 3, however for this one we apply a Charcoal Outlier model to all the UID charcoal dates. The results of Model 5 indicate good agreement. The Amodel (99.9) indicates good agreement, exceeding the 60-threshold for good agreement (see Table S19). The model estimates a start date for the ring of *4110-4020 cal. BP* (68.3 hpd) and an end date of *4050-3880 cal. BP* (68.3 hpd) and a start date for the ring of *4170-3990 cal. BP* (95.4 hpd) and an end date of *4060-3830 cal. BP* (95.4 hpd). The model identified no outliers.

Model 6 is the same as Model 5, however, a for this one we only apply the General model to all the dates and an apply a KDE command with a LnN(ln(125), ln (2) to summarize the probability distribution, which places emphasis on the earlier part of the distribution rather than later (i.e., < 125 years) (see above earlier discussion). The results of Model 6 indicate good agreement. The Amodel (113.3) for the model indicates good agreement, i.e., exceeding the 60-threshold (see Table S20). The model estimates a start date for the ring of *4100-4020 cal. BP* (68.3 hpd) and an end date of *3980-3910 cal. BP* (68.3 hpd) and a start date for the ring of *4140-3990 cal. BP* (95.4 hpd) and an end date of *4050-3880 cal. BP* (95.4 hpd). The Interval of occupation is estimated to be 40-150 years (68.3 hpd) and 20-230 (95.3 hpd

All the models except Model 1 exceed the values for good agreement for Bayesian analysis (i.e., Amodel is less than 60). The start and end dates for all the models are similar. Model 6 presents the most constrained date range. This model too likely represents the best estimate of site occupation based on the currently available data and the dates agree best with the assumptions and priors.

**Table S15:** 9LI1648R1M1


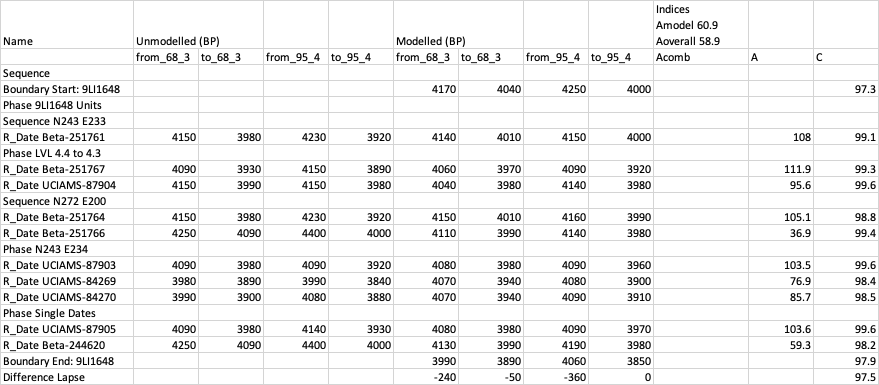


**Table S16:** 9LI1648R1M2

**
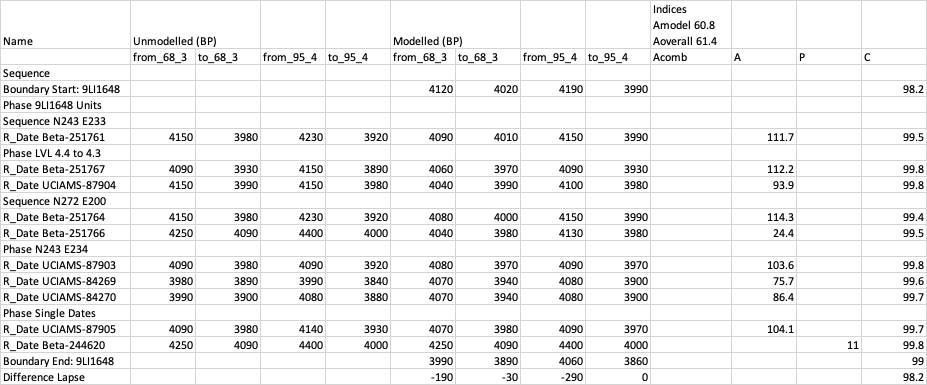
**

**Table S17:** 9LI1648R1M3

***
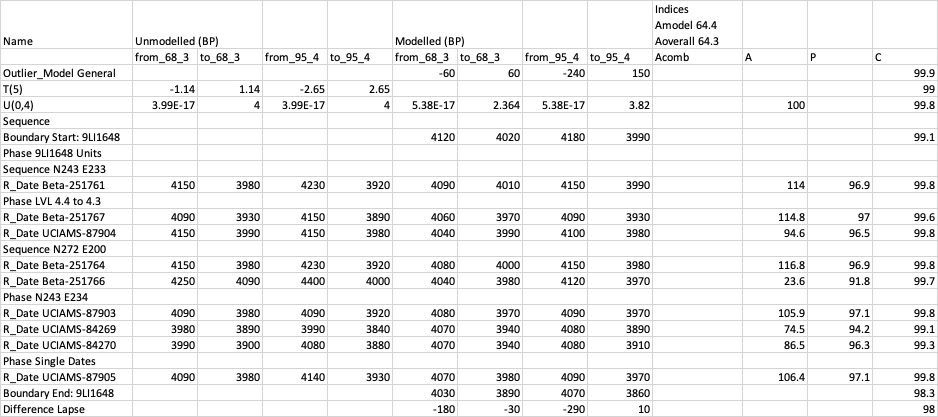
***

**Table S18:** 9LI1648R1M4

**
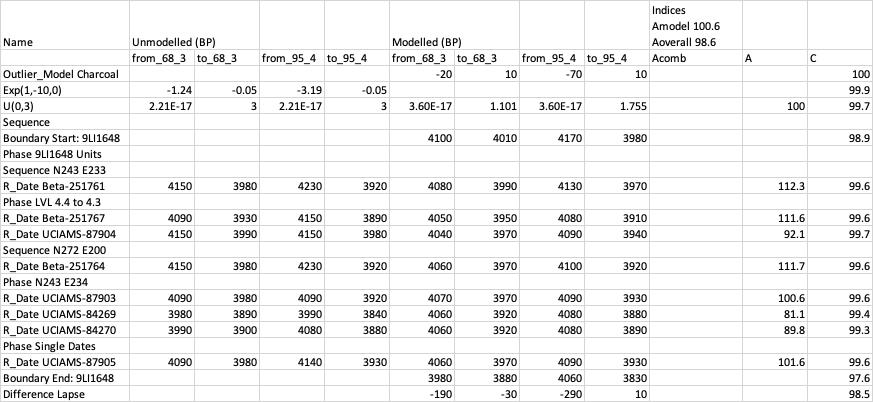
**

**Table S19:** 9LI1648M5


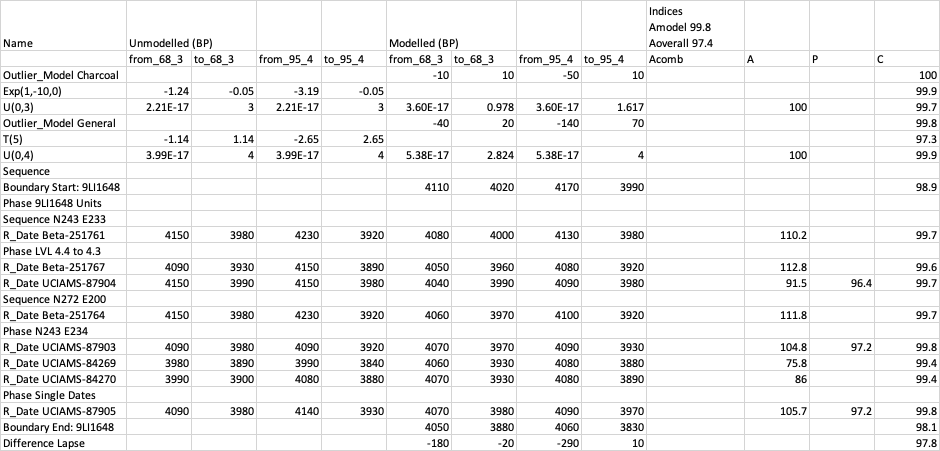


**Table S20:** 9LI1648M6


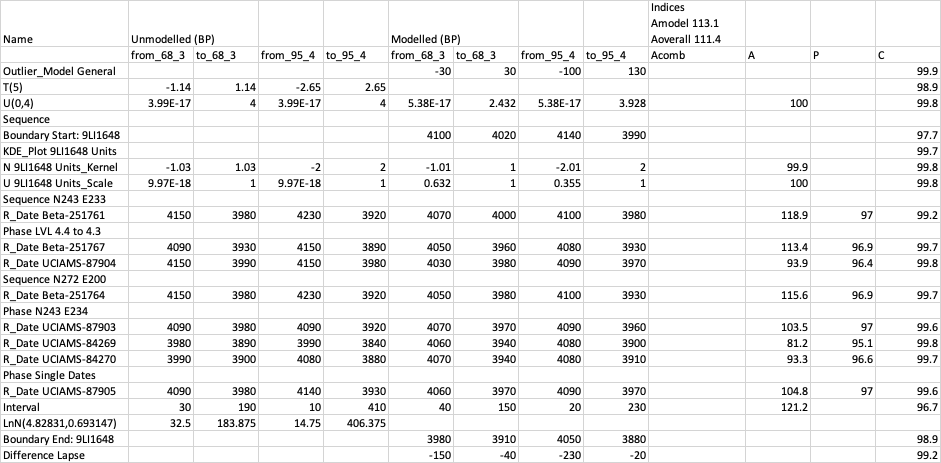


***38BU1866 Coosaw 2***

Coosaw Island Ring 2 samples come from test excavations described in Heide and Russo (2003) and shovel tests described in Smith (2023).

Model 1 places all the dates into one Phase. The order of the dates within each Sequence is the stratigraphic order that the excavators recovered each sample. The Amodel (97.9) ndicates good agreement between the parameters of the model and the order of dates (Table S21). The model estimates a start date for the ring of *4770-3720 cal. BP* (68.3 hpd) and an end date of *3680-2720 cal. BP* (68.3 hpd) and a start date for the ring of *4770-3710 cal. BP* (95.4 hpd) and an end date of *3690-2720 cal. BP* (95.4 hpd).

Model 2 is the same as Model 1, however, for this one we apply a General Outlier model to all the dates. The results of Model 2 indicate good agreement. The Amodel (62) for the model indicates good agreement, i.e., exceeding the 60-threshold (see Table S22). The model estimates a start date for the ring of *3840-3720 cal. BP* (68.3 hpd) and an end date of *3680-3560 cal. BP* (68.3 hpd) and a start date for the ring of *3980-3650 cal. BP* (95.4 hpd) and an end date of *3790-3420 cal. BP* (95.4 hpd).

Model 3 is identical to Model 2 in structure except that we apply a KDE command with a LnN(ln(125), ln (2) to summarize the probability distribution, which places emphasis on the earlier part of the distribution rather than later (i.e., < 125 years) (see above earlier discussion). The results of Model 3 indicate good agreement. The Amodel (75.2) for the model indicates good agreement, exceeding the 60-threshold (see Table S23). The model estimates a start date for the ring of *3860-3720 cal. BP* (68.3 hpd) and an end date of *3680-3550 cal. BP* (68.3 hpd) and a start date for the ring of *4010-3670 cal. BP* (95.4 hpd) and an end date of *3750-3380 cal. BP* (95.4 hpd). Interval of occupation is estimated to be 70-270 years (68.3 hpd) and 20-470 (95.3 hpd).

**Table S21:** 38BU1866R2M1

***
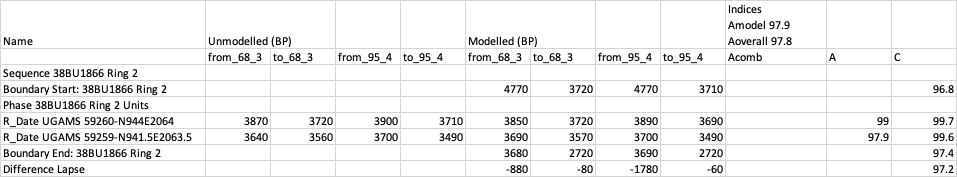
***

**Table S22:** 38BU1866R2M2

***
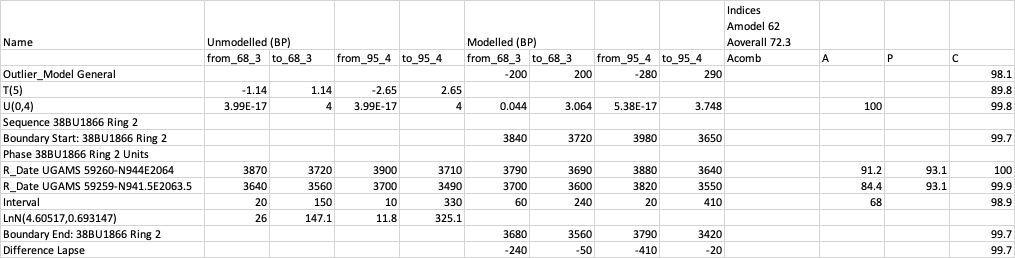
***

**Table S23:** 38BU1866R2M3

***
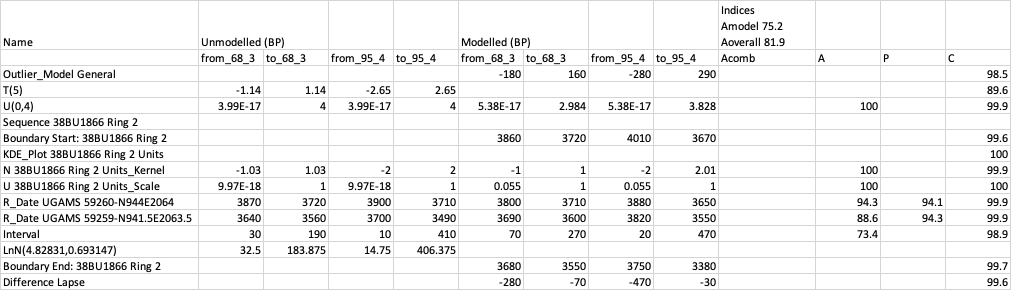
***

***38BU1866 Coosaw 3***

Coosaw Island Ring 3 samples come from test excavations described in Heide and Russo (2003).

Model 1 places all the dates into one Phase. The order of the dates within each Sequence is the stratigraphic order that the excavators recovered each sample. The results of Model 1 indicate good agreement. The Amodel (97.2) for the model indicates good agreement, i.e., exceeding the 60-threshold (see Table S24). The model estimates a start date for the ring of *3970-3730 cal. BP* (68.3 hpd) and an end date of *3830-3610 cal. BP* (68.3 hpd) and a start date for the ring of *4580-3720 cal. BP* (95.4 hpd) and an end date of *3840-3050 cal. BP* (95.4 hpd).

Model 2 is the same as Model 1, however, for this one we apply a General Outlier model to all the dates. The results of Model 2 indicate good agreement. The Amodel (99.7) for the model indicates good agreement, i.e., exceeding the 60-threshold (see Table S25). The model estimates a start date for the ring of *3960-3730 cal. BP* (68.3 hpd) and an end date of *3830-3620 cal. BP* (68.3 hpd) and a start date for the ring of *4590-3720 cal. BP* (95.4 hpd) and an end date of *3840-3040 cal. BP* (95.4 hpd).

Model 3 is identical to Model 2 in structure except that we apply a KDE command with a LnN(ln(125), ln (2) to summarize the probability distribution, which places emphasis on the earlier part of the distribution rather than later (i.e., < 125 years) (see above earlier discussion). The results of Model 3 indicate good agreement. The Amodel (75.2) for the model indicates good agreement, exceeding the 60-threshold (see Table S26). The model estimates a start date for the ring of *3890-3770 cal. BP* (68.3 hpd) and an end date of *3790-3680 cal. BP* (68.3 hpd) and a start date for the ring of *3970-3730 cal. BP* (95.4 hpd) and an end date of *3830-3600 cal. BP* (95.4 hpd). Interval of occupation is estimated to be 30-150 years (68.3 hpd) and 10-280 (95.3 hpd).

**Table S24:** 38BU1866R3M1


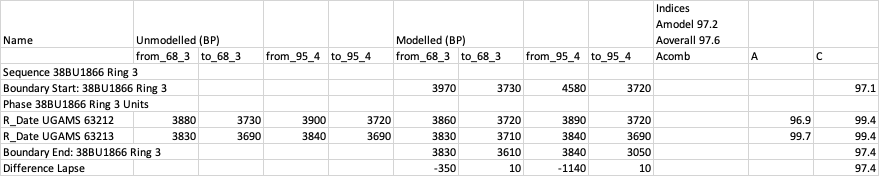


**Table S25:** 38BU1866R3M2


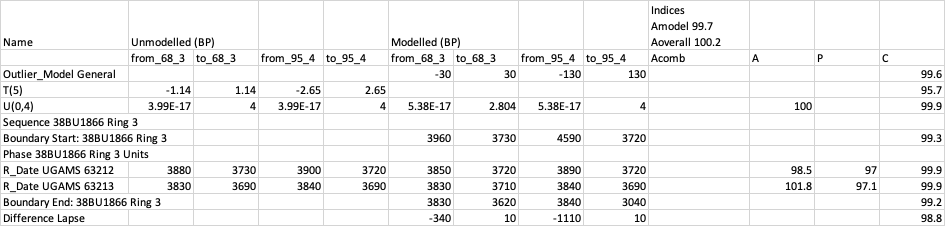


**Table S26:** 38BU1866R3M3


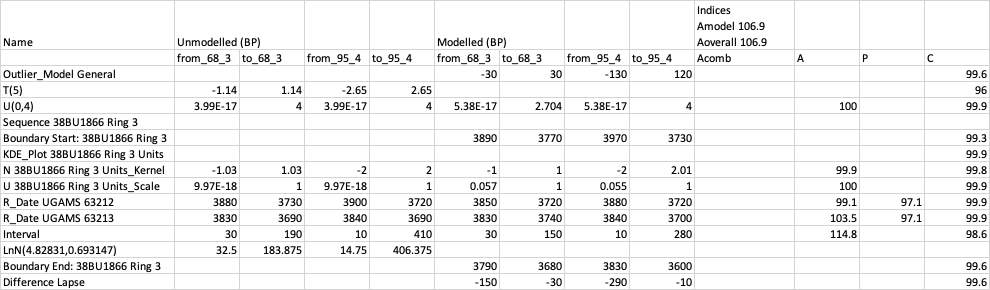


***38BU7 Sea Pines***

Model 1 places all the dates from the test units into multiple Phases and Sequences. The order of the dates within each Sequence is the stratigraphic order that the excavators recovered each sample. The results of Model 1 indicate good agreement. The Amodel (108.6) for the model indicates good agreement, exceeding the 60-threshold (see Table S27). The model estimates a start date for the ring of *4020-3950 cal. BP* (68.3 hpd) and an end date of *3840-3760 cal. BP* (68.3 hpd) and a start date for the ring of *4100-3930 cal. BP* (95.4 hpd) and an end date of *3870-3680 cal. BP* (95.4 hpd).

Model 2 is the same as Model 1, however for this one we apply a General Outlier model to all the dates. The results of Model 3 indicate good agreement, and does not identify an outlier. The Amodel (112.8) for the model indicates good agreement, i.e., exceeding the 60-threshold (see Table S28). The model estimates a start date for the ring of *4020-3950 cal. BP* (68.3 hpd) and an end date of *3840-3760 cal. BP* (68.3 hpd) and a start date for the ring of *4100-3930 cal. BP* (95.4 hpd) and an end date of *3880-3680 cal. BP* (95.4 hpd).

Model 3 is the same as Model 2, however, here we use the Charcoal Outlier model in place of th e General Outlier model. The results of Model 3 indicate good agreement, and does not identify an outlier. The Amodel (106.7) for the model indicates good agreement, i.e., exceeding the 60-threshold (see Table S29). The model estimates a start date for the ring of *4020-3940 cal. BP* (68.3 hpd) and an end date of *3900-3750 cal. BP* (68.3 hpd) and a start date for the ring of *4100-3900 cal. BP* (95.4 hpd) and an end date of *3900-3700 cal. BP* (95.4 hpd).

Model 4 is identical to Model 3 in structure except that we apply a KDE command with a LnN(ln(125), ln (2) to summarize the probability distribution, which places emphasis on the earlier part of the distribution rather than later (i.e., < 125 years) (see above earlier discussion). The results of Model 4 indicate good agreement. The Amodel (96) for the model indicates good agreement, exceeding the 60-threshold (see Table S30). The model estimates a start date for the ring of *4000-3940 cal. BP* (68.3 hpd) and an end date of *3850-3760 cal. BP* (68.3 hpd) and a start date for the ring of *4050-3920 cal. BP* (95.4 hpd) and an end date of *39120-3710 cal. BP* (95.4 hpd). Interval of occupation is estimated to be 110-220 years (68.3 hpd) and 40-300 (95.3 hpd).

**Table S27**: 38BU7R1M1


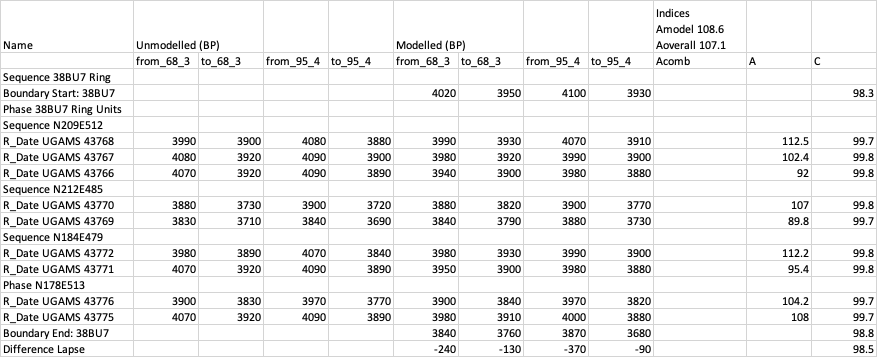


**Table S28**: 38BU7R1M2

***
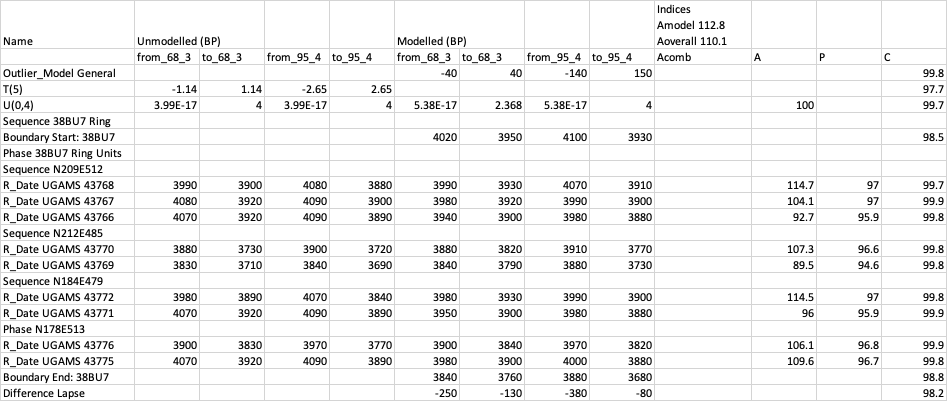
***

**Table S29**: 38BU7R1M3

***
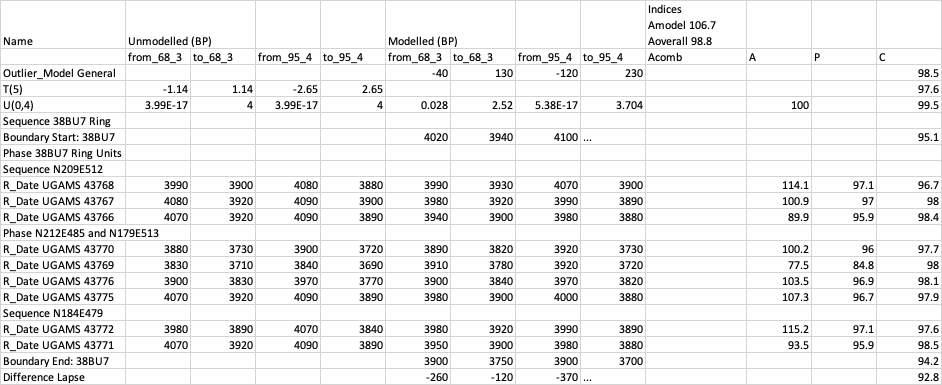
***

**Table S30**: 38BU7R1M4

***
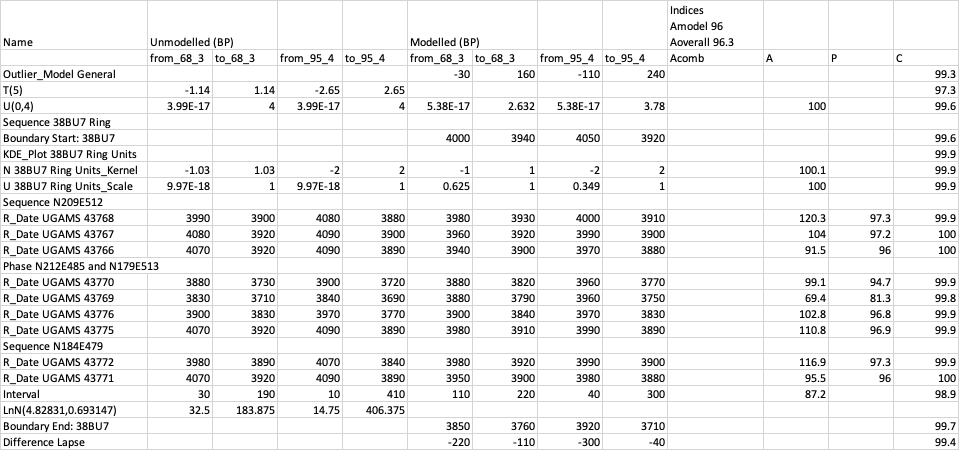
***

***9CH111 Odingsell***

Model 1 places all the dates from the test units into one Sequence. The order of the dates within the Sequence is the stratigraphic order that the excavators recovered each sample. The results of Model 1 indicate good agreement. The Amodel (82.6) for the model indicates good agreement, exceeding the 60-threshold (see Table S31). The model estimates a start date for the ring of *4720-4280 cal. BP* (68.3 hpd) and an end date of *3830-3390 cal. BP* (68.3 hpd) and a start date for the ring of *5450-4260 cal. BP* (95.4 hpd) and an end date of *3850-2640 cal. BP* (95.4 hpd).

Model 2 is the same as Model 1, however for this one we apply a General Outlier model to all the dates. The results of Model 2 indicate good agreement and do not identify an outlier. The Amodel (85.9) for the model indicates good agreement, i.e., exceeding the 60-threshold (Table S32). The model estimates a start date for the ring of *4750-4280 cal. BP* (68.3 hpd) and an end date of *3830-3370 cal. BP* (68.3 hpd) and a start date for the ring of *5450-4260 cal. BP* (95.4 hpd) and an end date of *3840-2640 cal. BP* (95.4 hpd).

Model 3 is the same as Model 1, however for this one we apply a Charcoal Outlier model to all the dates. The results of Model 3 indicate good agreement and do not identify an outlier. The Amodel (92.5) for the model indicates good agreement, i.e., exceeding the 60-threshold (see Table S33). The model estimates a start date for the ring of *5450-3830 cal. BP* (68.3 hpd) and an end date of *3840-3400 cal. BP* (68.3 hpd) and a start date for the ring of *5450-3780 cal. BP* (95.4 hpd) and an end date of *3850-2640 cal. BP* (95.4 hpd).

Model 4 applies both a General and Charcoal Outlier model. The results of Model 4 indicate good agreement and do not identify an outlier. The Amodel (92.9) for the model indicates good agreement, i.e., exceeding the 60-threshold (see Table S34). The model estimates a start date for the ring of *5450-4160 cal. BP* (68.3 hpd) and an end date of *3830-2640 cal. BP* (68.3 hpd) and a start date for the ring of *5460-3960 cal. BP* (95.4 hpd) and an end date of *3840-2640 cal. BP* (95.4 hpd).

Model 5 is identical to Model 1 in structure except that we apply a KDE command with a LnN(ln(125), ln (2) to summarize the probability distribution, which places emphasis on the earlier part of the distribution rather than later (i.e., < 125 years) (see above earlier discussion). The results of Model 5 indicate good agreement. The Amodel (104.6) for the model indicates good agreement, exceeding the 60-threshold (see Table S35). The model estimates a start date for the ring of *4430-4300 cal. BP* (68.3 hpd) and an end date of *4340-4210 cal. BP* (68.3 hpd) and a start date for the ring of *4510-4250 cal. BP* (95.4 hpd) and an end date of *4390-4120 cal. BP* (95.4 hpd). Interval of occupation is estimated to be 30-150 years (68.3 hpd) and 10-290 (95.3 hpd).

**Table S31:** 9CH111R1M1

**Table S32:** 9CH111R1M2

***
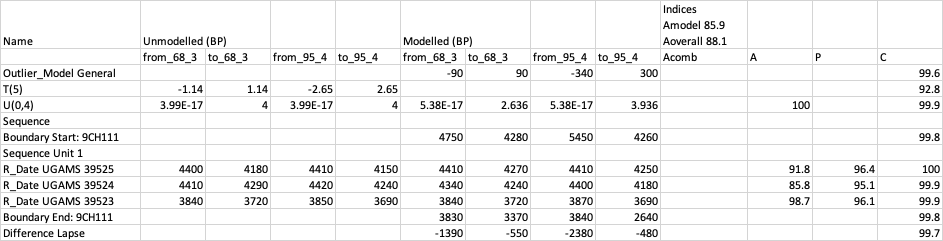
***

**Table S33:** 9CH111R1M3

***
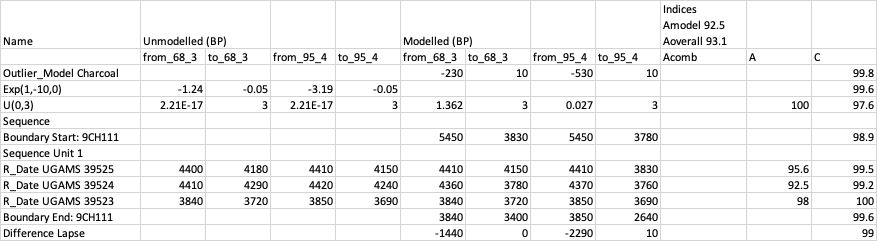
***

**Table S34:** 9CH111R1M4

***
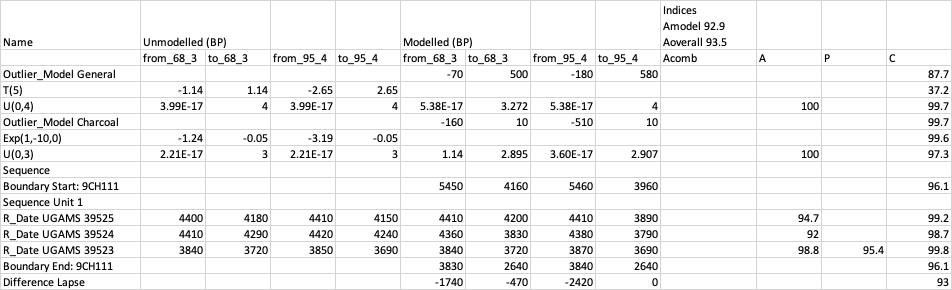
***

**Table S35:** 9CH111R1M5

***
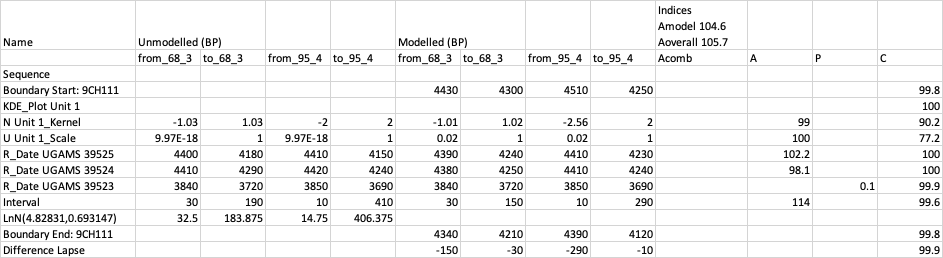
***

***9CH160 Hokfv-Mocvse***

Model 1 places all the dates from the test units into one Phase that contains several ordered Sequences. The order of the dates within each Sequence is the stratigraphic order that the excavators recovered each sample that include both shell dense layers and non-shell layers under the ring. The results of Model 1 indicate good agreement. The Amodel (114) for the model indicates good agreement, exceeding the 60-threshold (see Table S36). The model estimates a start date for the ring of *5060-4920 cal. BP* (68.3 hpd) and an end date of *4840-4790 cal. BP* (68.3 hpd) and a start date for the ring of *5090-4900 cal. BP* (95.4 hpd) and an end date of *4850-4730 cal. BP* (95.4 hpd).

Model 2 is the same as Model 1, however for this one we apply a General Outlier model to all the dates. The results of Model 2 indicate good agreement and do not identify an outlier. The Amodel (116.6) for the model indicates good agreement, i.e., exceeding the 60-threshold (see Table S37). The model estimates a start date for the ring of *5060-4890 cal. BP* (68.3 hpd) and an end date of *4840-4790 cal. BP* (68.3 hpd) and a start date for the ring of *5080-4880 cal. BP* (95.4 hpd) and an end date of *4870-4740 cal. BP* (95.4 hpd).

Model 3 is identical to Model 2 in structure except that we apply a KDE command with a LnN(ln(125), ln (2) to summarize the probability distribution, which places emphasis on the earlier part of the distribution rather than later (i.e., < 125 years) (see above earlier discussion). The results of Model 3 indicate good agreement. The Amodel (95.2) for the model indicates good agreement, exceeding the 60-threshold (see Table S38). The model estimates a start date for the ring of *5040-4910 cal. BP* (68.3 hpd) and an end date of *4840-4800 cal. BP* (68.3 hpd) and a start date for the ring of *5060-4880 cal. BP* (95.4 hpd) and an end date of *4870-4770 cal. BP* (95.4 hpd). Interval of occupation is estimated to be 80-230 years (68.3 hpd) and 20-270 (95.3 hpd).

**Table S36:** 9CH160R1M1

**
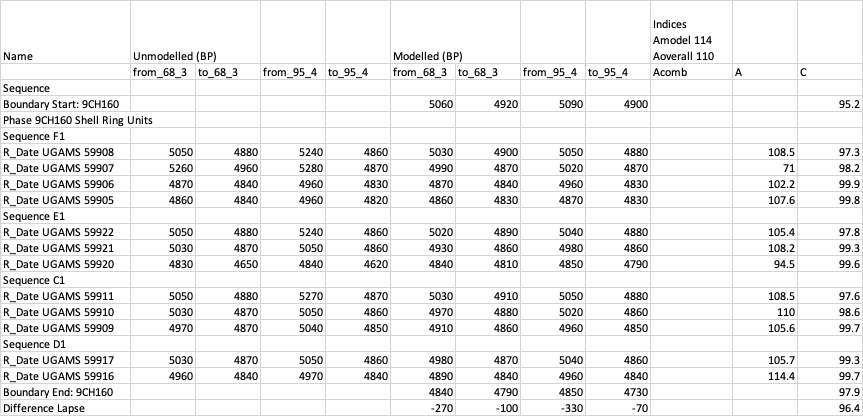
**

**Table S37:** 9CH160R1M2

***
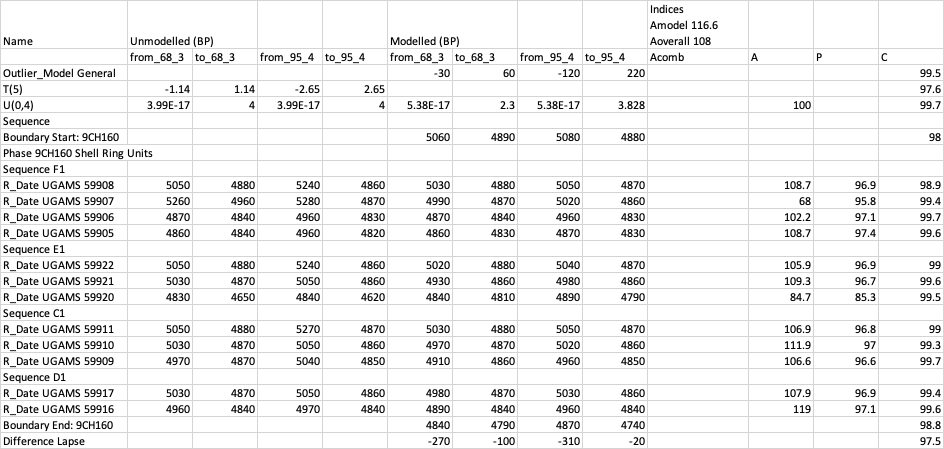
***

**Table S38:** 9CH160R1M3

***
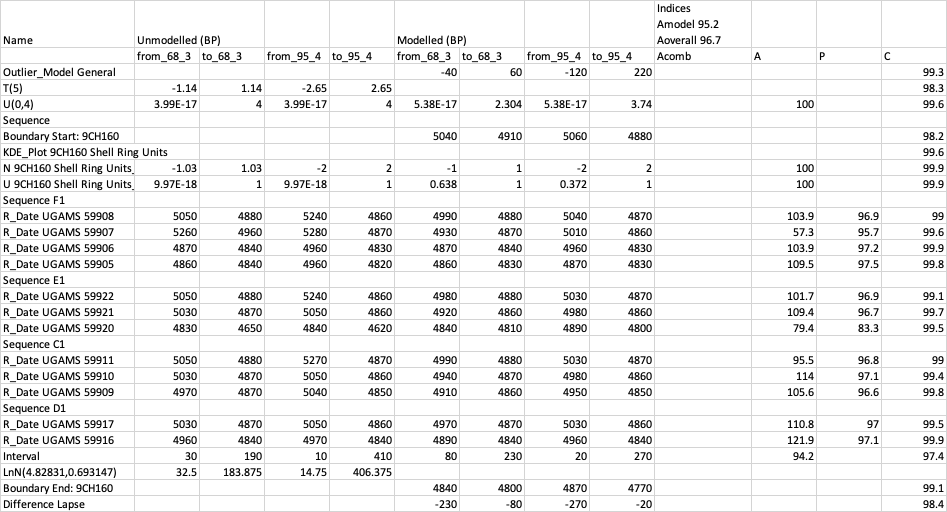
***

***9CH203 Ossabaw Shell Ring***

Model 1 places all the dates from the test units into a single Sequence. The order of the dates within the Sequence is the stratigraphic order that the excavators recovered each sample. The results of Model 1 indicate good agreement. The Amodel (82.1) for the model indicates good agreement, exceeding the 60-threshold (see Table S39). The model estimates a start date for the ring of *4280-4000 cal. BP* (68.3 hpd) and an end date of *4150-3920 cal. BP* (68.3 hpd) and a start date for the ring of *4540-4000 cal. BP* (95.4 hpd) and an end date of *4160-3700 cal. BP* (95.4 hpd).

Model 2 is the same as Model 1, however for this one we apply a General Outlier model to all the dates. The results of Model 2 indicate good agreement and do not identify an outlier. The Amodel (97.7) for the model indicates good agreement, i.e., exceeding the 60-threshold (see Table S40). The model estimates a start date for the ring of *4310-4100 cal. BP* (68.3 hpd) and an end date of *4060-3900 cal. BP* (68.3 hpd) and a start date for the ring of *4590-4080 cal. BP* (95.4 hpd) and an end date of *4140-3660 cal. BP* (95.4 hpd).

Model 3 is identical to Model 2 in structure except that we apply a KDE command with a LnN(ln(125), ln (2) to summarize the probability distribution, which places emphasis on the earlier part of the distribution rather than later (i.e., < 125 years) (see above earlier discussion). The results of Model 3 indicate good agreement. The Amodel (107.3) for the model indicates good agreement, exceeding the 60-threshold (see Table S41). The model estimates a start date for the ring of *4340-4150 cal. BP* (68.3 hpd) and an end date of *4010-3890 cal. BP* (68.3 hpd) and a start date for the ring of *4580-4090 cal. BP* (95.4 hpd) and an end date of *4060-3780 cal. BP* (95.4 hpd). Interval of occupation is estimated to be 20-110 years (68.3 hpd) and 10-200 (95.3 hpd).

**Table S39:** 9CH203R1M1

***
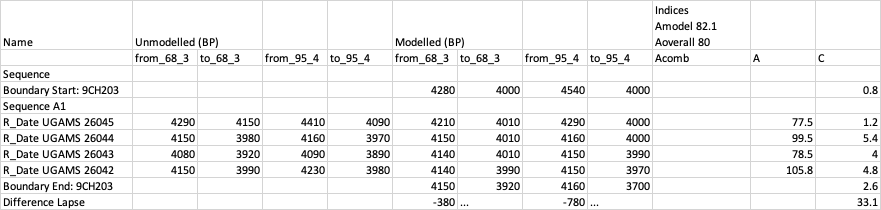
***

**Table S40:** 9CH203R1M2

***
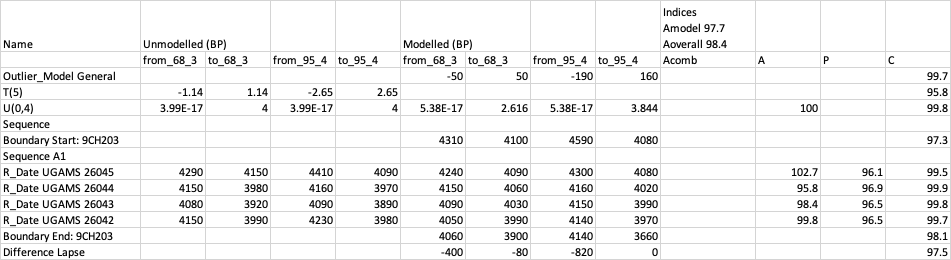
***

**Table S41:** 9CH203R1M3

**
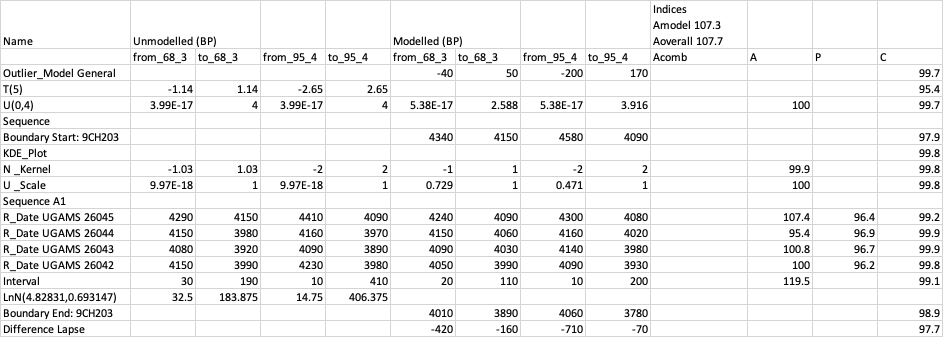
**

***9CH35 Cane Patch***

Model 1 places all the dates from the test units into one Phase that contains several ordered Sequences. The order of the dates within each Sequence is the stratigraphic order that the excavators recovered each sample. The results of Model 1 indicate good agreement. The Amodel (99.1) for the model indicates good agreement, exceeding the 60-threshold (see Table S42). The model estimates a start date for the ring of *4220-4100 cal. BP* (68.3 hpd) and an end date of *3890-3790 cal. BP* (68.3 hpd) and a start date for the ring of *4320-4090 cal. BP* (95.4 hpd) and an end date of *3960-3700 cal. BP* (95.4 hpd).

Model 2 is the same as Model 1, however for this one we apply a General Outlier model to all the dates. The results of Model 2 indicate good agreement and do not identify an outlier. The Amodel (103.1) for the model indicates good agreement, i.e., exceeding the 60-threshold (see Table S43). The model estimates a start date for the ring of *4230-4100 cal. BP* (68.3 hpd) and an end date of *3890-3790 cal. BP* (68.3 hpd) and a start date for the ring of *4330-4010 cal. BP* (95.4 hpd) and an end date of *3960-3710 cal. BP* (95.4 hpd).

Model 3 is the same as Model 1, however for this one we apply a Charcoal Outlier model to all the dates. The results of Model 3 indicate good agreement and do not identify an outlier. The Amodel (98.7) for the model indicates good agreement, i.e., exceeding the 60-threshold (see Table S44). The model estimates a start date for the ring of *4240-4080 cal. BP* (68.3 hpd) and an end date of *3880-3770 cal. BP* (68.3 hpd) and a start date for the ring of *4300-3970 cal. BP* (95.4 hpd) and an end date of *3950-3680 cal. BP* (95.4 hpd).

Model 4 is identical to Model 2 in structure except that we apply a KDE command with a LnN(ln(125), ln (2) to summarize the probability distribution, which places emphasis on the earlier part of the distribution rather than later (i.e., < 125 years) (see above earlier discussion). The results of Model 4 indicate good agreement. The Amodel (83.4) for the model indicates good agreement, exceeding the 60-threshold (see Table S45). The model estimates a start date for the ring of *4210-4100 cal. BP* (68.3 hpd) and an end date of *3900-3800 cal. BP* (68.3 hpd) and a start date for the ring of *4280-4010 cal. BP* (95.4 hpd) and an end date of *3970-3760 cal. BP* (95.4 hpd). Interval of occupation is estimated to be 220-390 years (68.3 hpd) and 60-480 (95.3 hpd).

**Table S42:** 9CH35R1M1

***
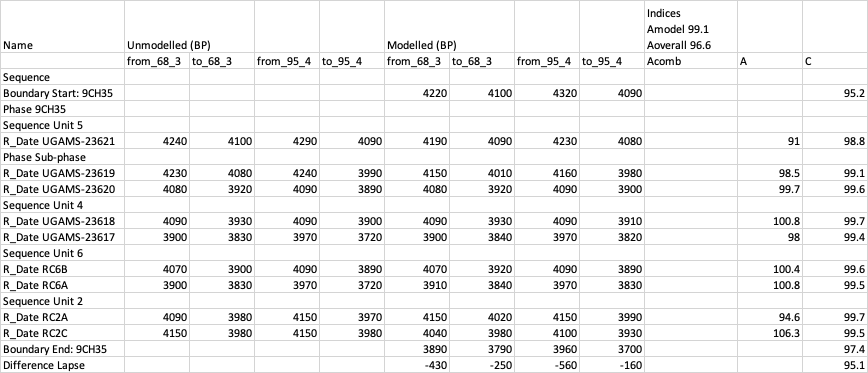
***

**Table S43:** 9CH35R1M2

**
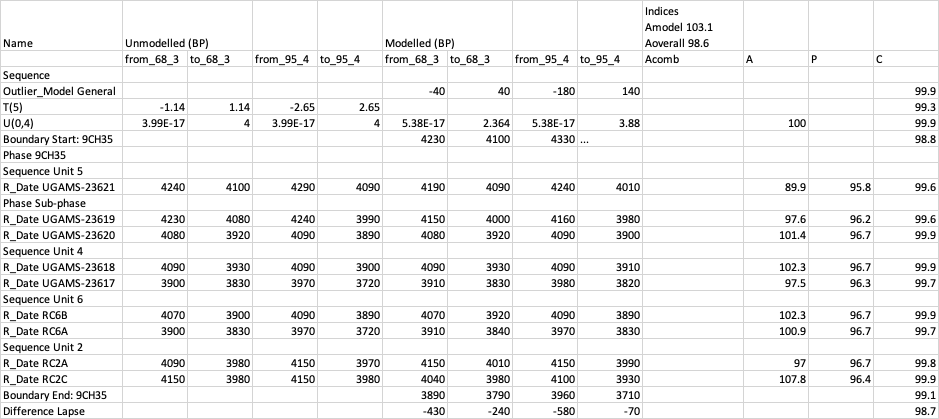
**

**Table S44:** 9CH35R1M3

**
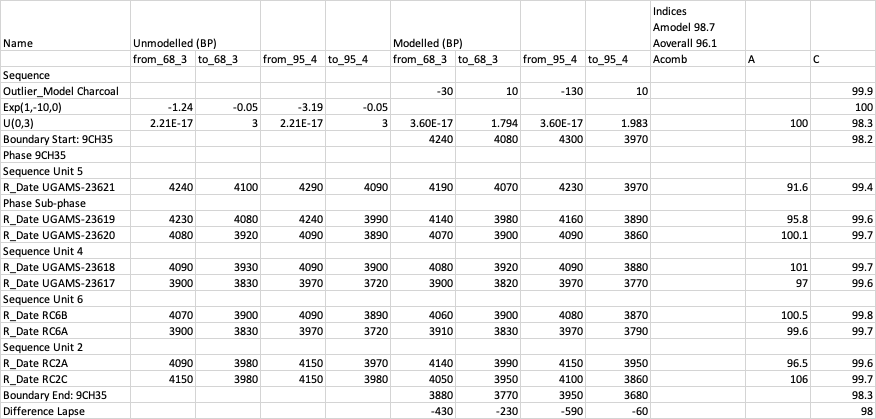
**

**Table S45:** 9CH35R1M4

***
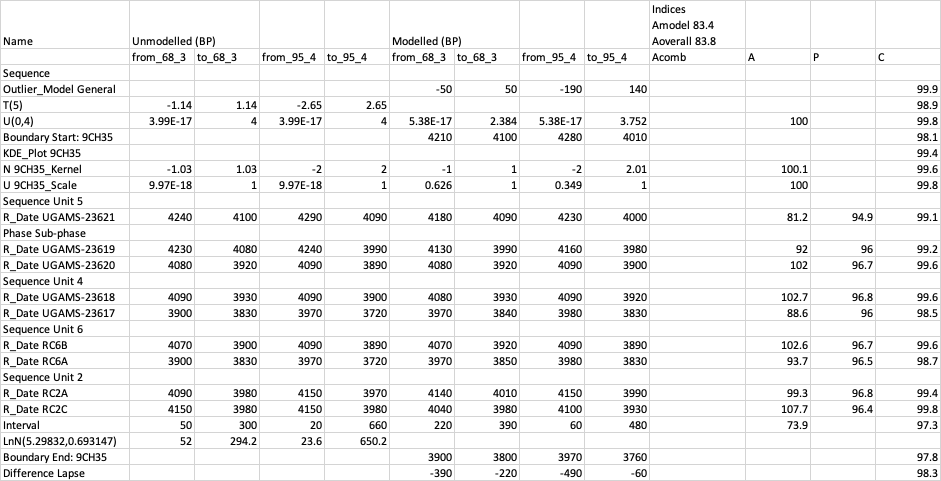
***

***9CH4 Bilbo***

Model 1 places all the dates from the test units into multiple Phase. The order of the dates within Phase is the stratigraphic order that the excavators recovered each sample. The results of Model 1 indicate good agreement. The Amodel (93.9) for the model indicates good agreement, exceeding the 60-threshold (see Table S46). The model estimates a start date for the ring of *4730-4260 cal. BP* (68.3 hpd) and an end date of *4110-3720 cal. BP* (68.3 hpd) and a start date for the ring of *5120-4100 cal. BP* (95.4 hpd) and an end date of *4290-3360 cal. BP* (95.4 hpd).

Model 2 is the same as Model 1, however for this one we apply a General Outlier model to all the dates. The results of Model 2 indicate good agreement and do not identify an outlier. The Amodel (103) for the model indicates good agreement, i.e., exceeding the 60-threshold (see Table S47). The model estimates a start date for the ring of *4600-4320 cal. BP* (68.3 hpd) and an end date of *3680-3390 cal. BP* (68.3 hpd) and a start date for the ring of *5140-4260 cal. BP* (95.4 hpd) and an end date of *3820-2840 cal. BP* (95.4 hpd).

Model 3 is the same as Model 1, however for this one we apply a Charcoal Outlier model to all the dates. The results of Model 3 indicate good agreement and do not identify an outlier. The Amodel (102.5) for the model indicates good agreement, i.e., exceeding the 60-threshold (see Table S48). The model estimates a start date for the ring of *4750-4140 cal. BP* (68.3 hpd) and an end date of *4060-3640 cal. BP* (68.3 hpd) and a start date for the ring of *5130-3810 cal. BP* (95.4 hpd) and an end date of *4230-3210 cal. BP* (95.4 hpd).

Model 4 is identical to Model 2 in structure except that we apply a KDE command with a LnN(ln(125), ln (2) to summarize the probability distribution, which places emphasis on the earlier part of the distribution rather than later (i.e., < 125 years) (see above earlier discussion). The results of Model 4 indicate good agreement. The Amodel (136.8) for the model indicates good agreement, exceeding the 60-threshold (see Table S49). The model estimates a start date for the ring of *4220-4000 cal. BP* (68.3 hpd) and an end date of *4090-3880 cal. BP* (68.3 hpd) and a start date for the ring of *4360-3920 cal. BP* (95.4 hpd) and an end date of *4210-3790 cal. BP* (95.4 hpd). Interval of occupation is estimated to be 30-180 years (68.3 hpd) and 10-350 (95.3 hpd).

**Table S46:** 9CH4R1M1

**
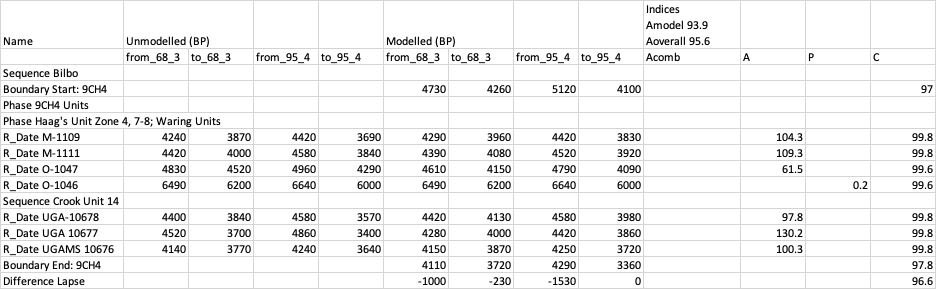
**

**Table S47:** 9CH4R1M2

***
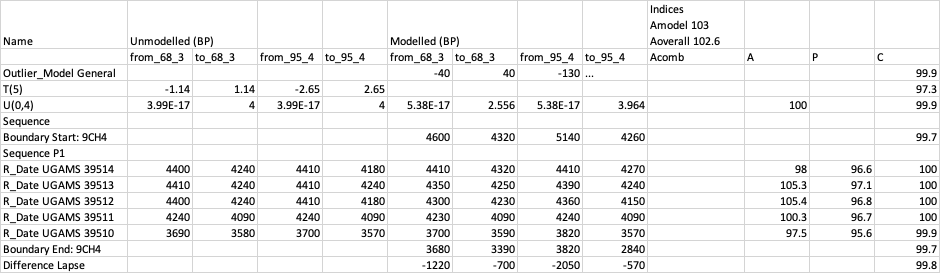
***

**Table S48:** 9CH4R1M3

***
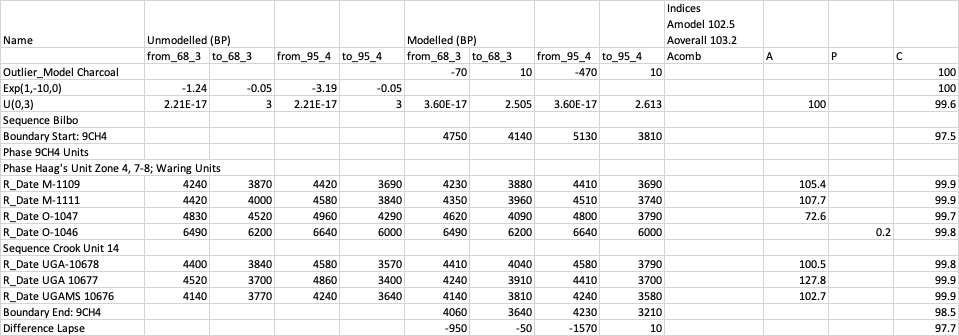
***

**Table S49:** 9CH4R1M4


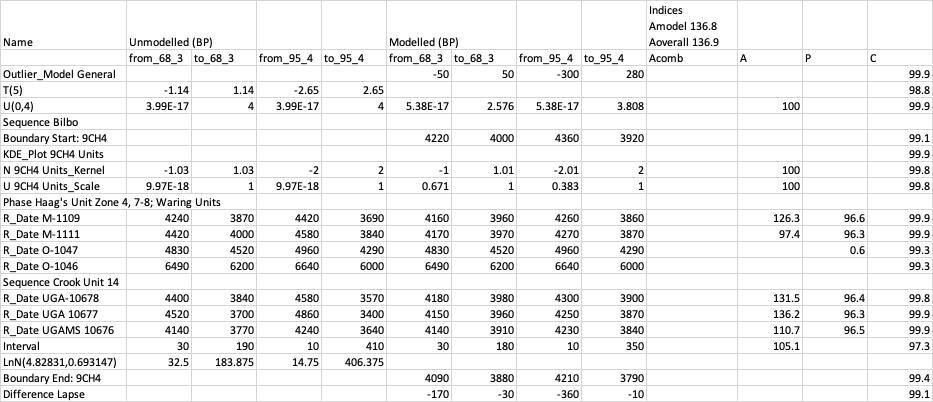


***9CH61 Pagan Plum***

Model 1 places all the dates from the test units into one Sequence. The order of the dates within the Sequence is the stratigraphic order that the excavators recovered each sample. The results of Model 1 indicate good agreement. The Amodel (99.6) for the model indicates good agreement, exceeding the 60-threshold (see Table S50). The model estimates a start date for the ring of *4610-4320 cal. BP* (68.3 hpd) and an end date of *3680-3400 cal. BP* (68.3 hpd) and a start date for the ring of *5140-4260 cal. BP* (95.4 hpd) and an end date of *3810-2830 cal. BP* (95.4 hpd).

Model 2 is the same as Model 1, however for this one we apply a General Outlier model to all the dates. The results of Model 2 indicate good agreement and do not identify an outlier. The Amodel (100.8) for the model indicates good agreement, i.e., exceeding the 60-threshold (see Table S51). The model estimates a start date for the ring of *4620-4310 cal. BP* (68.3 hpd) and an end date of *3690-3330 cal. BP* (68.3 hpd) and a start date for the ring of *5210-4260 cal. BP* (95.4 hpd) and an end date of *4100-2720 cal. BP* (95.4 hpd).

Model 3 is the same as Model 1, however for this one we apply a Charcoal Outlier model to all the dates. The results of Model 3 indicate good agreement and do not identify an outlier. The Amodel (97.8) for the model indicates good agreement, i.e., exceeding the 60-threshold (see Table S52). The model estimates a start date for the ring of *4590-4310 cal. BP* (68.3 hpd) and an end date of *3680-3410 cal. BP* (68.3 hpd) and a start date for the ring of *5120-4250 cal. BP* (95.4 hpd) and an end date of *3810-2850 cal. BP* (95.4 hpd).

Model 4 is the same as Model 1, however for this one we apply a General and Charcoal Outlier model to all the dates. The results of Model 4 indicate good agreement and do not identify an outlier. The Amodel (99.3) for the model indicates good agreement, i.e., exceeding the 60-threshold (see Table S53). The model estimates a start date for the ring of *4610-4310 cal. BP* (68.3 hpd) and an end date of *3680-3380 cal. BP* (68.3 hpd) and a start date for the ring of *5180-4250 cal. BP* (95.4 hpd) and an end date of *3820-2750 cal. BP* (95.4 hpd).

Model 5 is identical to Model 2 in structure except that we apply a KDE command with a LnN(ln(125), ln (2) to summarize the probability distribution, which places emphasis on the earlier part of the distribution rather than later (i.e., < 125 years) (see above earlier discussion). The results of Model 5 indicate good agreement. The Amodel (110.2) for the model indicates good agreement, exceeding the 60-threshold (see Table S54). The model estimates a start date for the ring of *5090-4330 cal. BP* (68.3 hpd) and an end date of *3620-3470 cal. BP* (68.3 hpd) and a start date for the ring of *5090-4310 cal. BP* (95.4 hpd) and an end date of *3650-3400 cal. BP* (95.4 hpd). Interval of occupation is estimated to be 30-140 years (68.3 hpd) and 10-220 (95.3 hpd).

**Table S50:** 9CH61R1M1

***
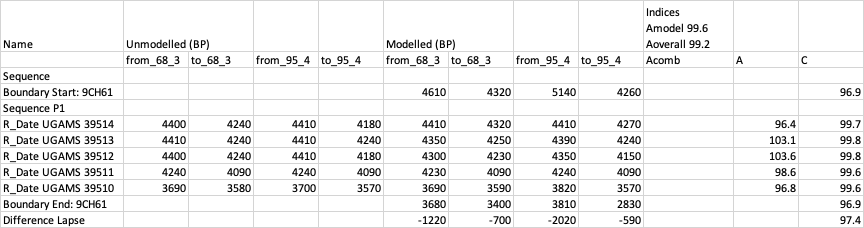
***

**Table S51:** 9CH61R1M2

***
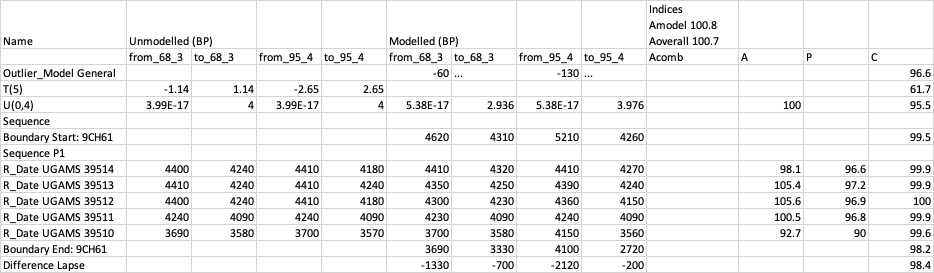
***

**Table S52:** 9CH61R1M3

**
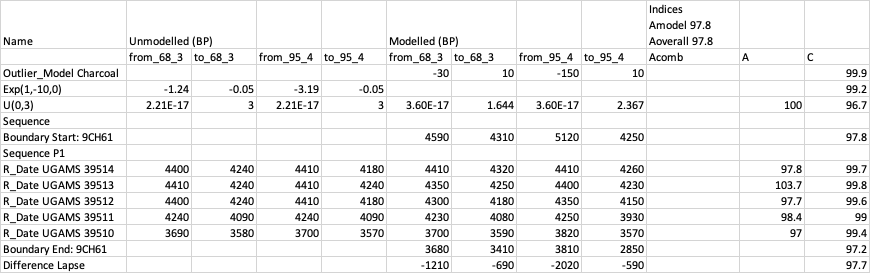
**

**Table S53:** 9CH61R1M4


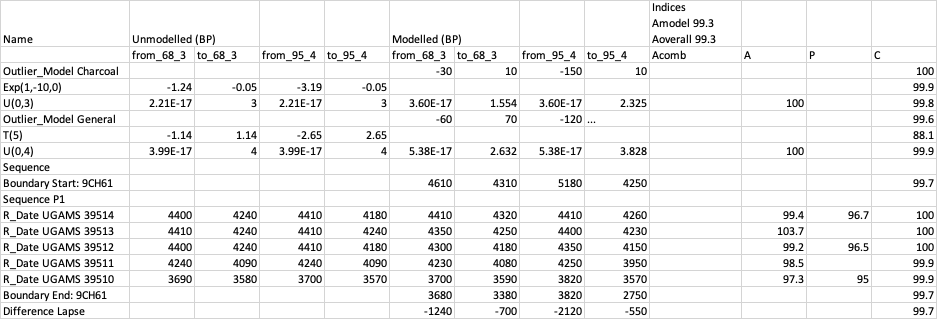


**Table S54:** 9CH61R1M5

***
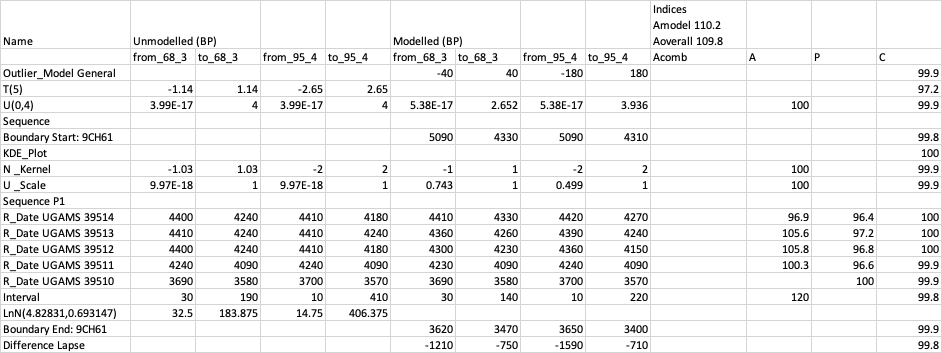
***

***9MC23 Sapelo Shell Ring 1***

Model 1 places all the dates from the test units into multiple Phases. The order of the dates is the stratigraphic order that the excavators recovered each sample. The results of Model 1 indicate good agreement. The Amodel (86.7) for the model indicates good agreement, i.e., exceeding the 60-threshold (see Table S55). The model estimates a start date for the ring of *4250-4200 cal. BP* (68.3 hpd) and an end date of *4150-4090 cal. BP* (68.3 hpd) and a start date for the ring of *4270-4160 cal. BP* (95.4 hpd) and an end date of *4160-4010 cal. BP* (95.4 hpd).

Model 2 is the same as Model 1; however, UGAMS 15084 was identified as an outlier. The Amodel (141.7) for the model indicates good agreement, i.e., exceeding the 60-threshold (see Table S56). The model estimates a start date for the ring of *4250-4170 cal. BP* (68.3 hpd) and an end date of *4160-4110 cal. BP* (68.3 hpd) and a start date for the ring of *4270-4160 cal. BP* (95.4 hpd) and an end date of *4230-4050 cal. BP* (95.4 hpd).

Model 3 is the same as Model 1, however for this one we apply a General and Charcoal Outlier model to all the dates. The results of Model 4 indicate good agreement and do not identify an outlier. The Amodel (139.9) for the model indicates good agreement, i.e., exceeding the 60-threshold (see Table S57). The model estimates a start date for the ring of *4250-4170 cal. BP* (68.3 hpd) and an end date of *4160-4110 cal. BP* (68.3 hpd) and a start date for the ring of *4270-4160 cal. BP* (95.4 hpd) and an end date of *4220-4050 cal. BP* (95.4 hpd).

Model 4 is the same as Model 2, however for this one we apply a General and Charcoal Outlier model to all the dates. The results of Model 4 indicate good agreement and do not identify an outlier. The Amodel (139.9) for the model indicates good agreement, i.e., exceeding the 60-threshold (see Table S58). The model estimates a start date for the ring of *4250-4170 cal. BP* (68.3 hpd) and an end date 4160-4110 *cal. BP* (68.3 hpd) and a start date for the ring of *4270-4160 cal. BP* (95.4 hpd) and an end date of *4220-4050 cal. BP* (95.4 hpd).

Model 5 is the same as Model 4 in structure except that we apply a KDE command with a LnN(ln(125), ln (2) to summarize the probability distribution, which places emphasis on the earlier part of the distribution rather than later (i.e., < 125 years) (see above earlier discussion). The results of Model 5 indicate good agreement and do not identify an outlier. The Amodel (149.4) for the model indicates good agreement, i.e., exceeding the 60-threshold (see Table S59). The model estimates a start date for the ring of *4250-4190 cal. BP* (68.3 hpd) and an end date 4150-4110 *cal. BP* (68.3 hpd) and a start date for the ring of *4260-4170 cal. BP* (95.4 hpd) and an end date of *4180-4060 cal. BP* (95.4 hpd). Interval of occupation is estimated to be 50-120 years (68.3 hpd) and 10-160 (95.3 hpd).

**Table S55:** 9MC23R1M1


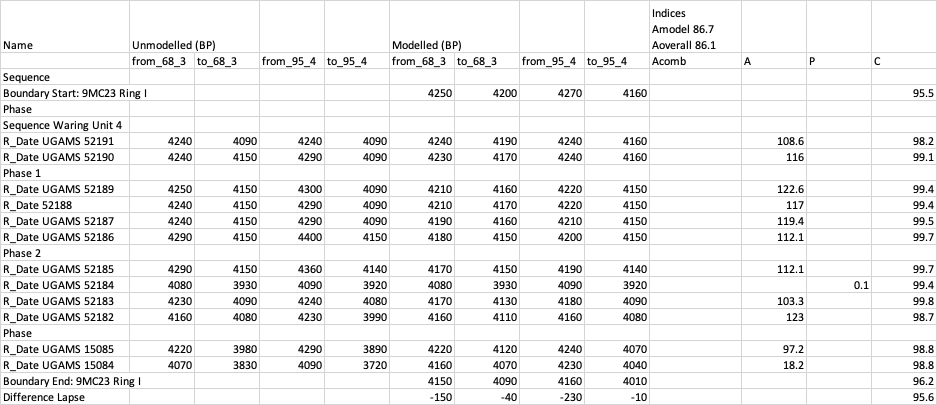


**Table S56:** 9MC23R1M2

**
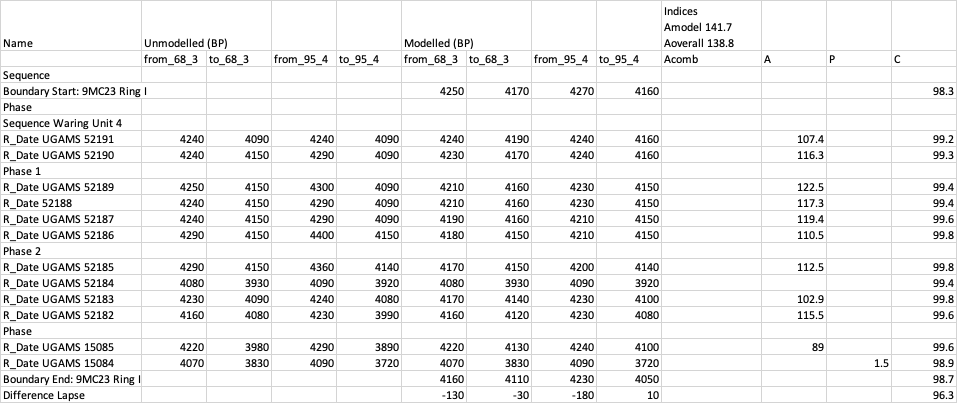
**

**Table S57:** 9MC23R1M3

***
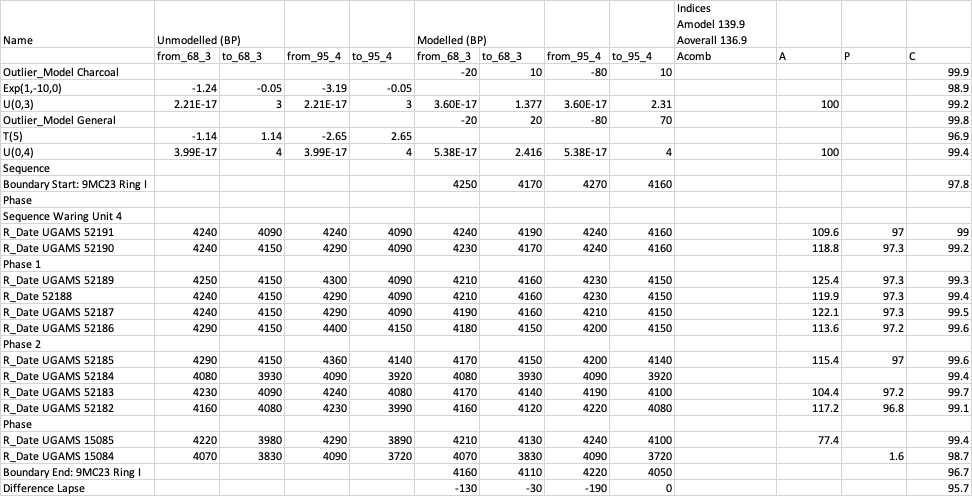
***

**Table S58:** 9MC23R1M4

**
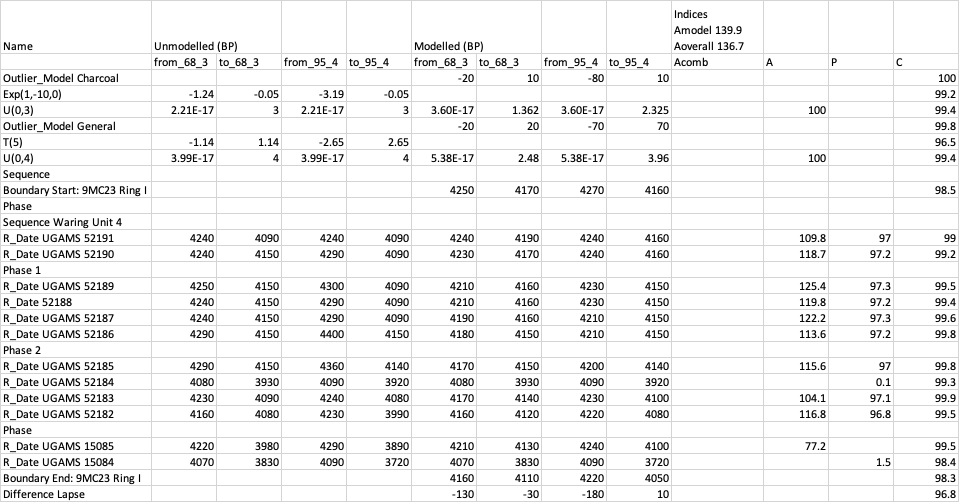
**

**Table S59:** 9MC23R1M5

***
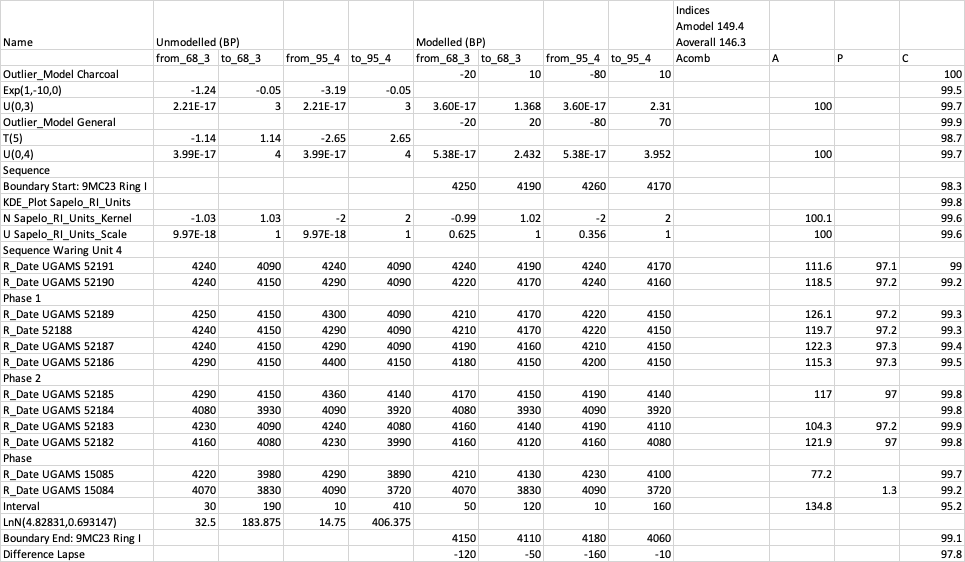
***

***9MC23 Sapelo Shell Ring 2***

Model 1 places all the dates from the test units into multiple Phases. The order of the dates is the stratigraphic order that the excavators recovered each sample. The results of Model 1 indicate good agreement. The Amodel (89.8) for the model indicates good agreement, exceeding the 60-threshold (see Table S60). The model estimates a start date for the ring of *4260-4150 cal. BP* (68.3 hpd) and an end date of *4090-3970 cal. BP* (68.3 hpd) and a start date for the ring of *4390-4100 cal. BP* (95.4 hpd) and an end date of *4150-3860 cal. BP* (95.4 hpd).

Model 2 is the same as Model 1, however for this one we apply a General Outlier model to all the dates. The results of Model 2 indicate good agreement and do not identify an outlier. The Amodel (92.3) for the model indicates good agreement, i.e., exceeding the 60-threshold (see Table S61). The model estimates a start date for the ring of *4270-4150 cal. BP* (68.3 hpd) and an end date of *4115-3970 cal. BP* (68.3 hpd) and a start date for the ring of *4400-4100 cal. BP* (95.4 hpd) and an end date of *4210-3820 cal. BP* (95.4 hpd).

Model 3 is the same as Model 1, however for this one we apply a Charcoal Outlier model to all the dates. The results of Model 3 indicate good agreement and do not identify an outlier. The Amodel (103.9) for the model indicates good agreement, i.e., exceeding the 60-threshold (see Table S62). The model estimates a start date for the ring of *4220-4070 cal. BP* (68.3 hpd) and an end date of *4090-3970 cal. BP* (68.3 hpd) and a start date for the ring of *4360-3990 cal. BP* (95.4 hpd) and an end date of *4140-3810 cal. BP* (95.4 hpd).

Model 4 applies a General and Charcol Outlier model and we apply a KDE command with a LnN(ln(125), ln (2) to summarize the probability distribution, which places emphasis on the earlier part of the distribution rather than later (i.e., < 125 years) (see above earlier discussion). The results of Model 5 indicate good agreement and do not identify an outlier. The Amodel (109.2) for the model indicates good agreement, i.e., exceeding the 60-threshold (see Table 63). The model estimates a start date for the ring of *4200-4080 cal. BP* (68.3 hpd) and an end date 4080-3990 *cal. BP* (68.3 hpd) and a start date for the ring of *4280-4030 cal. BP* (95.4 hpd) and an end date of *4130-3910 cal. BP* (95.4 hpd). Interval of occupation is estimated to be 30-160 years (68.3 hpd) and 10-260 (95.3 hpd).

**Table S60:** 9MC23R2M1

***
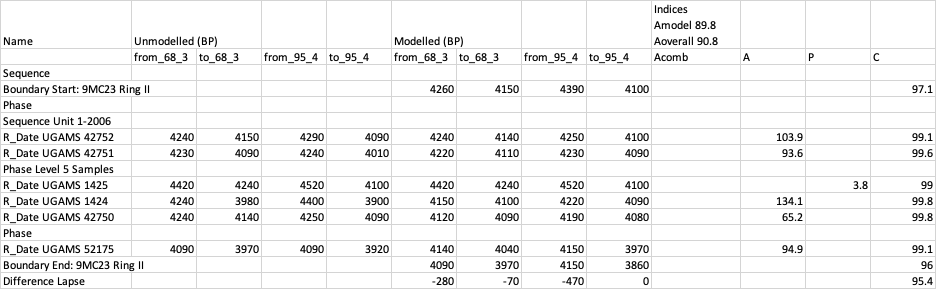
***

**Table S61:** 9MC23R2M2

***
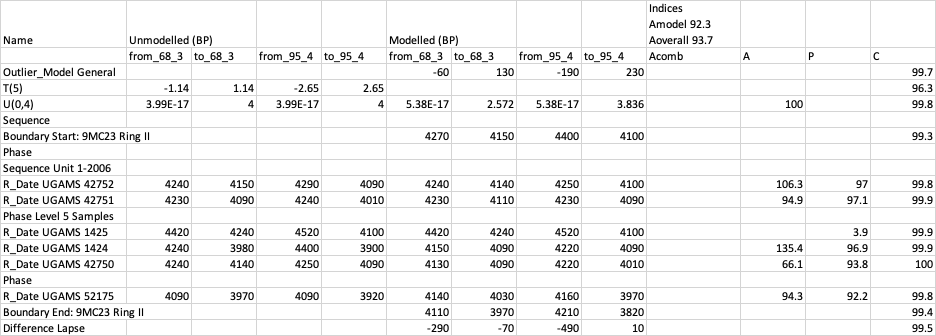
***

**Table S62:** 9MC23R2M3

***
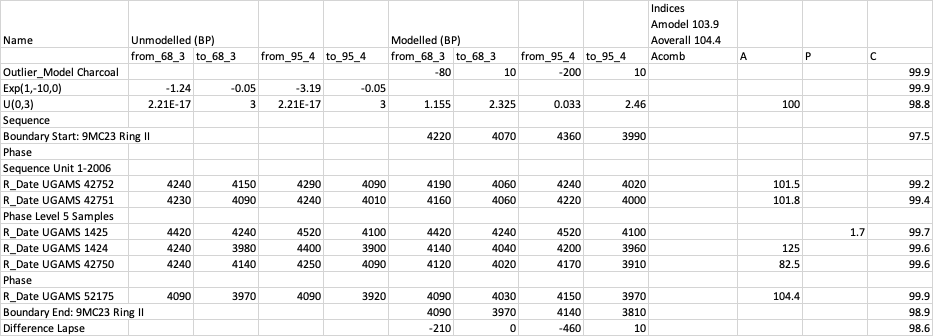
***

**Table S63:** 9MC23R2M4

***
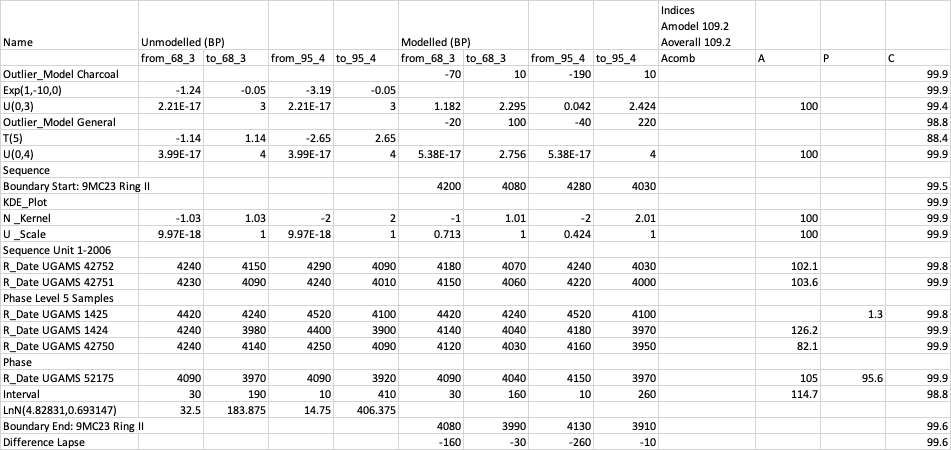
***

***9MC23 Sapelo Shell Ring 3***

Model 1 groups the dates into two phases with one sequence. The Amodel (93.7) for the model indicates good agreement, i.e., exceeding the 60-threshold (see Table S64). The model estimates a start date for the ring of *4110-3980 cal. BP* (68.3 hpd) and an end date of *3960-3840 cal. BP* (68.3 hpd) and a start date for the ring of *4230-3930 cal. BP* (95.4 hpd) and an end date of *3980-3750 cal. BP* (95.4 hpd).

Model 2 is smilar to Model 1 except the grouping of dates are put into one sequence with two phases. The results of Model 2 indicate good agreement and do not identify an outlier. The Amodel (100.5) for the model indicates good agreement, i.e., exceeding the 60-threshold (see Table S65). The model estimates a start date for the ring of *4200-4010 cal. BP* (68.3 hpd) and an end date of *3890-3800 cal. BP* (68.3 hpd) and a start date for the ring of *4270-4000 cal. BP* (95.4 hpd) and an end date of *3960-3720 cal. BP* (95.4 hpd).

Model 3 groups the dates into mulitple sequences and phases. The results of Model 3 indicate good agreement and do not identify an outlier The results of Model 4 indicate good agreement and do not identify an outlier. The Amodel (89.4) for the model indicates good agreement, i.e., exceeding the 60-threshold (see Table S66). The model estimates a start date for the ring of *4100-3980 cal. BP* (68.3 hpd) and an end date *3960-3830* *cal. BP* (68.3 hpd) and a start date for the ring of *4210-3930 cal. BP* (95.4 hpd) and an end date of *3970-3730cal. BP* (95.4 hpd).

Model 4 is the same as Model 3, however for this one we apply a General Outlier model to all the dates. The results of Model 4 indicate good agreement and do not identify an outlier. The Amodel (76.5) for the model indicates good agreement, i.e., exceeding the 60-threshold (see Table S67). The model estimates a start date for the ring of *4100-3980 cal. BP* (68.3 hpd) and an end date *3960-3850* *cal. BP* (68.3 hpd) and a start date for the ring of *4180-3930 cal. BP* (95.4 hpd) and an end date of *3980-3760 cal. BP* (95.4 hpd).

Model 5 is the same as Model 3, however for this one we apply a General and Charcoal Outlier model to all the dates. The results of Model 4 indicate good agreement and do not identify an outlier. The Amodel (80.9) for the model indicates good agreement, i.e., exceeding the 60-threshold (see Table S68). The model estimates a start date for the ring of *4100-3940 cal. BP* (68.3 hpd) and an end date *3950-3830* *cal. BP* (68.3 hpd) and a start date for the ring of *4180-3920 cal. BP* (95.4 hpd) and an end date of *3970-3720 cal. BP* (95.4 hpd).

Model 6 is the same as Model 6 in structure except that we apply a KDE command with a LnN(ln(125), ln (2) to summarize the probability distribution, which places emphasis on the earlier part of the distribution rather than later (i.e., < 125 years) (see above earlier discussion). The results of Model 5 indicate good agreement and do not identify an outlier. The Amodel (75) for the model indicates good agreement, i.e., exceeding the 60-threshold (see Table S69). The model estimates a start date for the ring of *4060-3970 cal. BP* (68.3 hpd) and an end date 3950-3860 *cal. BP* (68.3 hpd) and a start date for the ring of *4110-3930 cal. BP* (95.4 hpd) and an end date of 3970-3800 *cal. BP* (95.4 hpd). Interval of occupation is estimated to be 40-170 years (68.3 hpd) and 20-280 (95.3 hpd).

**Table S64:** 9MC23R3M1

***
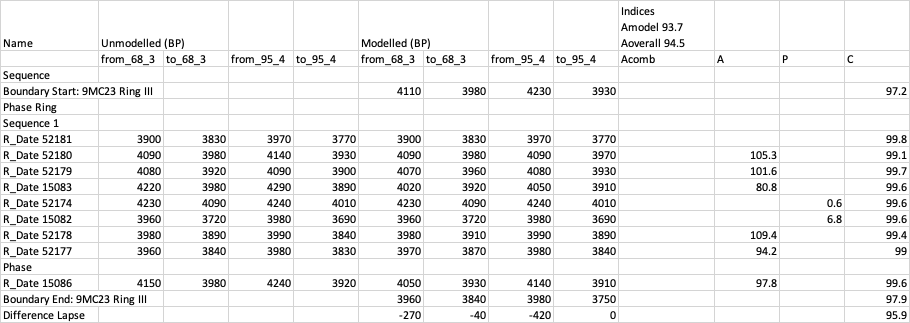
***

**Table S65:** 9MC23R3M2

**
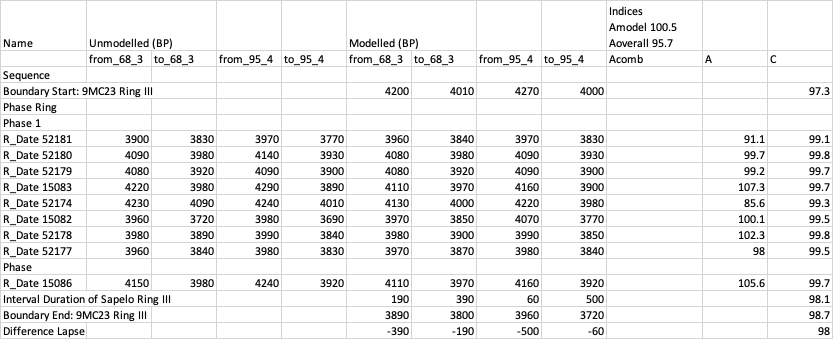
**

**Table S66:** 9MC23R3M3

***
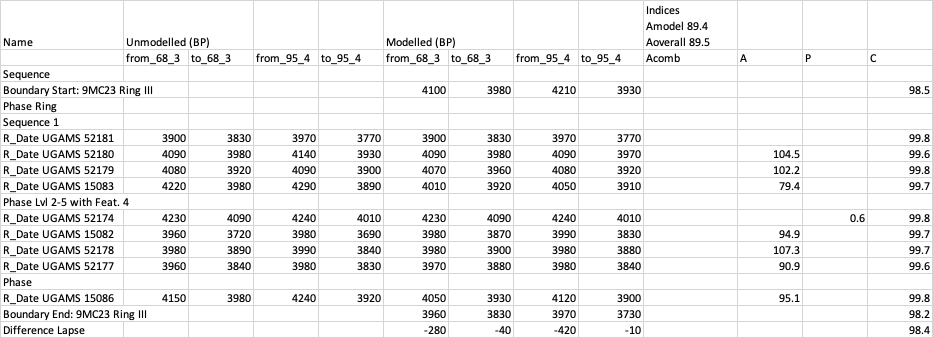
***

**Table S67:** 9MC23R3M4

***
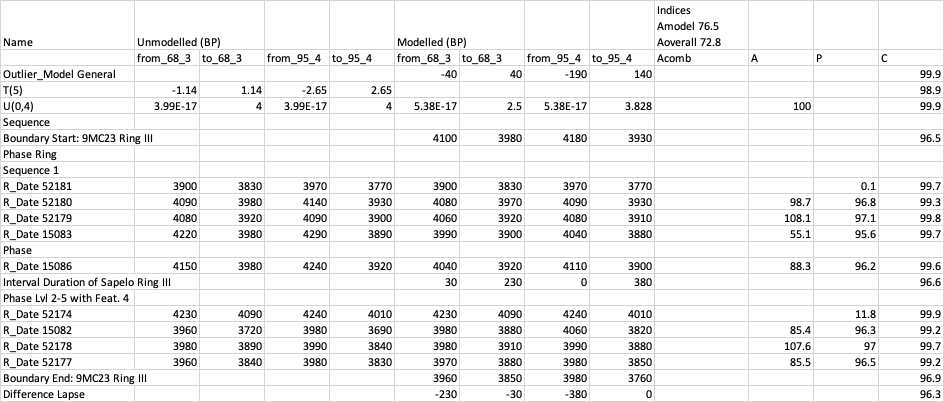
***

**Table S68:** 9MC23R3M5

***
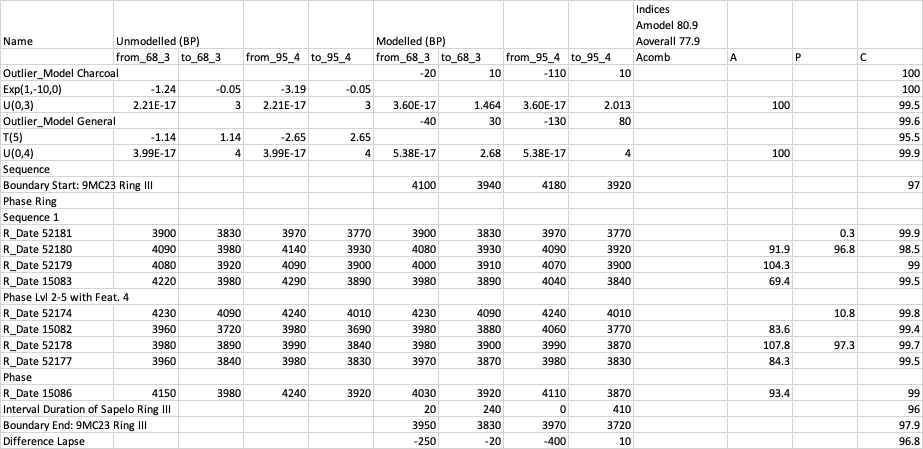
***

**Table S69:** 9MC23R3M6

***
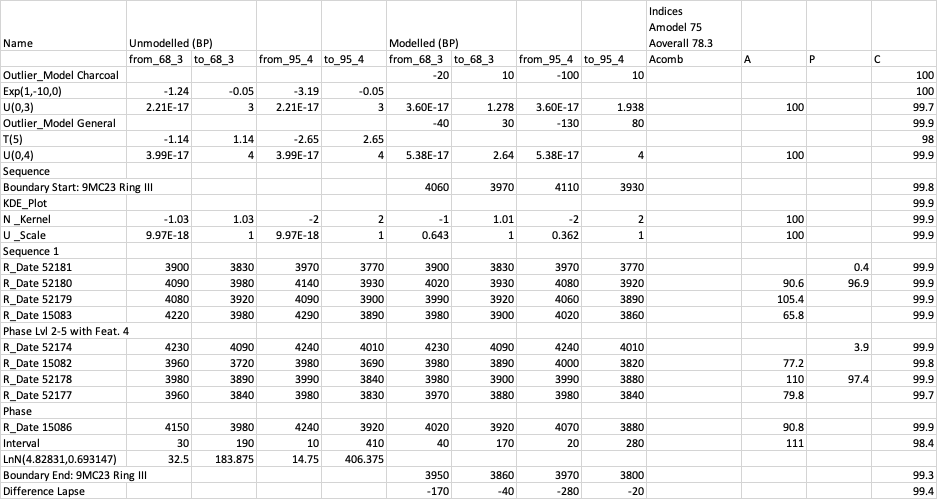
***

***9MC87 Creighton Shell Ring***

Model 1 places all the dates from the test units into one Phase that contains several ordered

Sequences. The order of the dates within each Sequence is the stratigraphic order that the excavators recovered each sample that include both shell dense layers and non-shell layers under the ring. The results of Model 1 indicate good agreement. The Amodel (109.6) for the model indicates good agreement, exceeding the 60-threshold (see Table S70). The model estimates a start date for the ring of *4430-4260 cal. BP* (68.3 hpd) and an end date of *4060-3860 cal. BP* (68.3 hpd) and a start date for the ring of *4640-4190 cal. BP* (95.4 hpd) and an end date of *4080-3680 cal. BP* (95.4 hpd).

Model 2 is the same as Model 1, however for this one we apply a General Outlier model to all the dates. The results of Model 2 indicate good agreement and do not identify an outlier. The Amodel (112.7) for the model indicates good agreement, i.e., exceeding the 60-threshold (see Table S71). The model estimates a start date for the ring of *4420-4250 cal. BP* (68.3 hpd) and an end date of *4070-3870 cal. BP* (68.3 hpd) and a start date for the ring of *4630-4170 cal. BP* (95.4 hpd) and an end date of *4080-3690 cal. BP* (95.4 hpd).

Model 3 is the same as Model 1, however for this one we apply a Charcoal Outlier model to all the dates. The results of Model 3 indicate good agreement and do not identify an outlier. The Amodel (113.5) for the model indicates good agreement, i.e., exceeding the 60-threshold (see Table S72). The model estimates a start date for the ring of *4420-4260 cal. BP* (68.3 hpd) and an end date of *4070-3860 cal. BP* (68.3 hpd) and a start date for the ring of *4640-4170 cal. BP* (95.4 hpd) and an end date of *4080-3680 cal. BP* (95.4 hpd).

Model 4 is the same as Model 1, however for this one we apply a General and Charcoal Outlier model to all the dates. The results of Model 4 indicate good agreement and do not identify an outlier. The Amodel (107.2) for the model indicates good agreement, i.e., exceeding the 60-threshold (see Table S73). The model estimates a start date for the ring of *4430-4240 cal. BP* (68.3 hpd) and an end date of *4070-3860 cal. BP* (68.3 hpd) and a start date for the ring of *4600-4145 cal. BP* (95.4 hpd) and an end date of *4090-3690 cal. BP* (95.4 hpd).

Model 5 is identical to Model 2 in structure except that we apply a KDE command with a LnN(ln(125), ln (2) to summarize the probability distribution, which places emphasis on the earlier part of the distribution rather than later (i.e., < 125 years) (see above earlier discussion). The results of Model 5 indicate good agreement. The Amodel (118.8) for the model indicates good agreement, exceeding the 60-threshold (see Table S74). The model estimates a start date for the ring of *4430-4260 cal. BP* (68.3 hpd) and an end date of *4030-3840 cal. BP* (68.3 hpd) and a start date for the ring of *4620-4180 cal. BP* (95.4 hpd) and an end date of *4050-3760 cal. BP* (95.4 hpd). Interval of occupation is estimated to be 20-100 years (68.3 hpd) and 10-190 (95.3 hpd).

**Table S70:** 9MC87R1M1

**Table S71:** 9MC87R1M2

**Table S72:** 9MC87R1M3

**Table S73:** 9MC87R1M5

**Table S74:** 9MC87R1M5

***38CH12 Lighthouse Point***

Lighthouse Point shell ring samples come from excavations conducted in 1976 and 1979 and described in Trinkley (1980).

Model 1 places all the dates from the units into one Phase. The order of the dates is the stratigraphic order that the excavators recovered each sample. The results of Model 1 indicate good agreement. The Amodel (72.9) for the model indicates good agreement, exceeding the 60-threshold (see Table S75). However, UGA2902 is in poor agreement. The model estimates a start date for the ring of *3620-3440 cal. BP* (68.3 hpd) and an end date of *3460-3260 cal. BP* (68.3 hpd) and a start date for the ring of *3840-3390 cal. BP* (95.4 hpd) and an end date of *3540-2900 cal. BP* (95.4 hpd).

Model 2 is the same as Model 1, however for this one we apply a General Outlier model to all the dates. The results of Model 2 indicate good agreement and do not identify an outlier. The Amodel (76.4) for the model indicates good agreement, exceeding the 60-threshold (see Table S76). However, UGA2902 is in poor agreement. The model estimates a start date for the ring of *3620-3440 cal. BP* (68.3 hpd) and an end date of *3460-3250 cal. BP* (68.3 hpd) and a start date for the ring of *3840-3390 cal. BP* (95.4 hpd) and an end date of *3540-2920 cal. BP* (95.4 hpd).

Model 3 is the same as Model 1, however for this one we apply a Charcoal Outlier model to all the dates. The results of Model 3 indicate good agreement and do not identify an outlier. The Amodel (79.9) for the model indicates good agreement, i.e., exceeding the 60-threshold (see Table S77). However, UGA2902 is in poor agreement. The model estimates a start date for the ring of *3620-3400 cal. BP* (68.3 hpd) and an end date of *3450-3200 cal. BP* (68.3 hpd) and a start date for the ring of *3870-3280 cal. BP* (95.4 hpd) and an end date of *3490-2790 cal. BP* (95.4 hpd).

Model 4 is identical to Model 2 in structure except that we apply a KDE command with a LnN(ln(125), ln (2) to summarize the probability distribution, which places emphasis on the earlier part of the distribution rather than later (i.e., < 125 years) (see above earlier discussion). The results of Model 5 indicate good agreement. The Amodel (66.9) for the model indicates good agreement, exceeding the 60-threshold (see Table S78). However, UGA2902 continues to be in poor agreement. The model estimates a start date for the ring of *3560-3450 cal. BP* (68.3 hpd) and an end date of *3450-3340 cal. BP* (68.3 hpd) and a start date for the ring of *3630-3400 cal. BP* (95.4 hpd) and an end date of *3490-3240 cal. BP* (95.4 hpd). Interval of occupation is estimated to be 30-170 years (68.3 hpd) and 0-310 (95.3 hpd).

**Table S75:** 38CH12M1

**Table S76:** 38CH12M2

**Table S77:** 38CH12M3

**Table S78:** 38CH12M4

***38CH42R1 Fig Ring 1***

Fig Island Ring 1 samples come from excavations described in Saunders (2002).

Model 1 places all the dates from the test units into one Phase and Sequence. The order of the dates within the Sequence is the stratigraphic order that the excavators recovered each sample. The results of Model 1 indicate good agreement. The Amodel (61.7) for the model indicates good agreement, exceeding the 60-threshold (see Table S79). However, UGAMS 52714 and UGAMS 53619 are in poor agreement. The model estimates a start date for the ring of *3930-3860 cal. BP* (68.3 hpd) and an end date of *3880-3800 cal. BP* (68.3 hpd) and a start date for the ring of *4020-3840 cal. BP* (95.4 hpd) and an end date of *3890-3700 cal. BP* (95.4 hpd).

Model 2 is the same as Model 1, however for this one we apply a General Outlier model to all the dates. The results of Model 2 indicate good agreement and do not identify an outlier. The Amodel (65.8) for the model indicates good agreement, i.e., exceeding the 60-threshold (see Table S80). However, UGAMS 52714 and UGAMS 53619 are in poor agreement. The model estimates a start date for the ring of *3930-3860 cal. BP* (68.3 hpd) and an end date of *3880-3800 cal. BP* (68.3 hpd) and a start date for the ring of *4000-3840 cal. BP* (95.4 hpd) and an end date of *3890-3710 cal. BP* (95.4 hpd).

Model 3 is identical to Model 2 in structure except that we apply a KDE command with a LnN(ln(125), ln (2) to summarize the probability distribution, which places emphasis on the earlier part of the distribution rather than later (i.e., < 125 years) (see above earlier discussion). The results of Model 5 indicate good agreement. The Amodel (79) for the model indicates good agreement, exceeding the 60-threshold (see Table S81). However, UGAMS 52714 and UGAMS 53619 are in poor agreement. The model estimates a start date for the ring of *3930-3870 cal. BP* (68.3 hpd) and an end date of *3870-3810 cal. BP* (68.3 hpd) and a start date for the ring of *3980-3850 cal. BP* (95.4 hpd) and an end date of *3880-3750 cal. BP* (95.4 hpd).

**Table S79:** 38CH42R1M1

**Table S80:** 38CH42R1M2

**Table S81:** 38CH42R1M3

***38CH42R2 Fig Ring 2***

Fig Island Ring 2 samples come from excavations described in Saunders (2002) and a 1m test unit on file with the Heritage Trust Program, South Carolina Department of Natural Resources.

Model 1 places all the dates from the test units into one Phase and Sequence. The order of the dates the Sequence is the stratigraphic order that the excavators recovered each sample. The results of Model 1 indicate good agreement. The Amodel (95.5) for the model indicates good agreement, exceeding the 60-threshold (see Table S82). The model estimates a start date for the ring of *4190-4020 cal. BP* (68.3 hpd) and an end date of *4070-3920 cal. BP* (68.3 hpd) and a start date for the ring of *4480-3980 cal. BP* (95.4 hpd) and an end date of *4090-3640 cal. BP* (95.4 hpd).

Model 2 is the same as Model 1, however for this one we apply a General Outlier model to all the dates. The results of Model 2 indicate good agreement and do not identify an outlier. The Amodel (97.9) for the model indicates good agreement, i.e., exceeding the 60-threshold (see Table S83). The model estimates a start date for the ring of *4200-4020 cal. BP* (68.3 hpd) and an end date of *4070-3910 cal. BP* (68.3 hpd) and a start date for the ring of *4550-3980 cal. BP* (95.4 hpd) and an end date of *4090-3570 cal. BP* (95.4 hpd).

Model 3 is identical to Model 1 in structure except that we apply a KDE command with a LnN(ln(125), ln (2) to summarize the probability distribution, which places emphasis on the earlier part of the distribution rather than later (i.e., < 125 years) (see above earlier discussion). The results of Model 5 indicate good agreement. The Amodel (106.6) for the model indicates good agreement, exceeding the 60-threshold (see Table S84). The model estimates a start date for the ring of *4150-4040 cal. BP* (68.3 hpd) and an end date of *4060-3960 cal. BP* (68.3 hpd) and a start date for the ring of *4210-3990 cal. BP* (95.4 hpd) and an end date of *4080-3880 cal. BP* (95.4 hpd). Interval of occupation is estimated to be 30-140 years (68.3 hpd) and 10-260 (95.3 hpd).

**Table S82:** 38CH42R2M1

**Table S83:** 38CH42R2M2

**Table S84:** 38CH42R2M3

***38CH42R3 Fig Ring 3***

Fig Island Ring 3 samples come from excavations described in Saunders (2002).

Model 1 places all the dates from the test units into one Phase and Sequence. The order of the dates within each Sequence is the stratigraphic order that the excavators recovered each sample. The results of Model 1 indicate good agreement. The Amodel (117.5) for the model indicates good agreement, exceeding the 60-threshold (see Table S85). The model estimates a start date for the ring of *4190-4090 cal. BP* (68.3 hpd) and an end date of *4150-4040 cal. BP* (68.3 hpd) and a start date for the ring of *4430-4080 cal. BP* (95.4 hpd) and an end date of *4160-3790 cal. BP* (95.4 hpd).

Model 2 is the same as Model 1, however for this one we apply a General Outlier model to all the dates. The results of Model 2 indicate good agreement and do not identify an outlier. The Amodel (118.1) for the model indicates good agreement, i.e., exceeding the 60-threshold (see Table S86). The model estimates a start date for the ring of *4200-4090 cal. BP* (68.3 hpd) and an end date of *4150-4030 cal. BP* (68.3 hpd) and a start date for the ring of *4490-4080 cal. BP* (95.4 hpd) and an end date of *4160-3740 cal. BP* (95.4 hpd).

Model 3 is identical to Model 1 in structure except that we apply a KDE command with a LnN(ln(125), ln (2) to summarize the probability distribution, which places emphasis on the earlier part of the distribution rather than later (i.e., < 125 years) (see above earlier discussion). The results of Model 5 indicate good agreement. The Amodel (124.6) for the model indicates good agreement, exceeding the 60-threshold (see Table S87). The model estimates a start date for the ring of *4190-4110 cal. BP* (68.3 hpd) and an end date of *4120-4050 cal. BP* (68.3 hpd) and a start date for the ring of *4260-4090 cal. BP* (95.4 hpd) and an end date of *4150-3970 cal. BP* (95.4 hpd). Interval of occupation is estimated to be 20-120 years (68.3 hpd) and 10-230 (95.3 hpd).

**Table S85:** 38CH42R3M1

**Table S86:** 38CH42R3M2

**Table S87:** 38CH42R3M3

***38CH62 Spanish Mount***

Spanish Mount samples come from Smith’s excavations currently undergoing analysis at the Heritage Trust Program, South Carolina Department of Natural Resources.

Model 1 places all the dates from test units into a single Phase that contains several ordered Sequences. The order of the dates within each Sequence is the stratigraphic order that the excavators recovered each sample. The results of Model 1 indicate good agreement. The Amodel (70.2) for the model indicates good agreement, exceeding the 60-threshold (see Table S88). However, the model identifies Beta 459730 and 459728 as outliers with poor agreement with the overall model (A=26.1 and 57.4, respectively). The model estimates a start date for the ring of *4070-3980 cal. BP* (68.3 hpd) and an end date of *3870-3790 cal. BP* (68.3 hpd) and a start date for the ring of *4170-3980 cal. BP* (95.4 hpd) and an end date of *3900-3730 cal. BP* (95.4 hpd).

Model 2 is the same as Model 1, however for this one we apply a General Outlier model to all the dates. The results of Model 1 indicate poor agreement. The Amodel (56.2) for the model do not indicate good agreement, i.e., exceeding the 60-threshold (Table S89).

Model 3 is the same as Model 1, however for this one we apply a Charcoal Outlier model to all the dates. The Amodel (85.7) for the model indicates good agreement, exceeding the 60-threshold (see Table S90). However, the model identifies Beta 459730 and 459728 as outliers with poor agreement with the overall model (A=54.8 and 52.9, respectively). The model estimates a start date for the ring of *4090-3940 cal. BP* (68.3 hpd) and an end date of *3870-3770 cal. BP* (68.3 hpd) and a start date for the ring of *4180-3920 cal. BP* (95.4 hpd) and an end date of *3900-3690 cal. BP* (95.4 hpd).

Model 4 is the same as Model 1, however for this one we apply a General and Charcoal Outlier model to all the dates. The results of Model 4 indicate good agreement and do not identify an outlier. The Amodel (91.6) for the model indicates good agreement, i.e., exceeding the 60-threshold (see Table S91). However, the model continues to identify Beta 459730 as an outlier with poor agreement with the overall model (A=58.4). The model estimates a start date for the ring of *4040-3930 cal. BP* (68.3 hpd) and an end date of *3910-3800 cal. BP* (68.3 hpd) and a start date for the ring of *4150-3920 cal. BP* (95.4 hpd) and an end date of *3960-3740 cal. BP* (95.4 hpd).

Model 5 is identical to Model 3 in structure except that we apply a KDE command with a LnN(ln(125), ln (2) to summarize the probability distribution, which places emphasis on the earlier part of the distribution rather than later (i.e., < 125 years) (see above earlier discussion). The results of Model 5 indicate good agreement. The Amodel (69.2) for the model indicates good agreement, exceeding the 60-threshold (see Table S92). However, Beta 459730 and 459728 as outliers with poor agreement with the overall model (A=47.3 and 46, respectively). The model estimates a start date for the ring of *4030-3930 cal. BP* (68.3 hpd) and an end date of *3880-3790 cal. BP* (68.3 hpd) and a start date for the ring of *4110-3920 cal. BP* (95.4 hpd) and an end date of *3920-3740 cal. BP* (95.4 hpd). Interval of occupation is estimated to be 110-230 years (68.3 hpd) and 25-330 (95.3 hpd).

**Table S88:** 38CH62M1

**Table S89:** 38CH62M2

**Table S90:** 38CH62M3

**Table S91:** 38CH62M4

**Table S92:** 38CH62M5

***38CH1781R1 Parrot Point A***

Parrot Point A samples come from excavations described in Baluha and Poplin (2005).

Model 1 places all the dates from the test units into one Phase that contains several ordered Sequences. The order of the dates within each Sequence is the stratigraphic order that the excavators recovered each sample. The results of Model 1 indicate poor agreement. The Amodel (25) for the model do not indicate good agreement, i.e., exceeding the 60-threshold (Table S93).

Model 2 is the same as Model 1, however for this one we apply a General Outlier model to all the dates. The results of Model 1 indicate poor agreement. The Amodel (29.5) for the model do not indicate good agreement, i.e., exceeding the 60-threshold (see Table S94).

Model 3 is the same as Model 1, however for this one we apply a Charcoal Outlier model to all the dates. The results of Model 1 indicate poor agreement. The Amodel (32.1) for the model do not indicate good agreement, i.e., exceeding the 60-threshold (see Table S95).

Model 4 is the same as Model 1, however for this one we apply a General and Charcoal Outlier model to all the dates. UGAMS 61231 was flagged as an outlier in the model. The results of Model 4 indicate good agreement and do not identify an outlier. The Amodel (101.5) for the model indicates good agreement, i.e., exceeding the 60-threshold (see Table S96). The model estimates a start date for the ring of *4580-4440 cal. BP* (68.3 hpd) and an end date of *4510-4370 cal. BP* (68.3 hpd) and a start date for the ring of *4760-4420 cal. BP* (95.4 hpd) and an end date of *4520-4210 cal. BP* (95.4 hpd).

Model 5 is identical to Model 4 in structure except that we apply a KDE command with a LnN(ln(125), ln (2) to summarize the probability distribution, which places emphasis on the earlier part of the distribution rather than later (i.e., < 125 years) (see above earlier discussion). The results of Model 5 indicate good agreement. The Amodel (111) for the model indicates good agreement, exceeding the 60-threshold (see Table S97). The model estimates a start date for the ring of *4560-4460 cal. BP* (68.3 hpd) and an end date of *4500-4380 cal. BP* (68.3 hpd) and a start date for the ring of *4620-4430 cal. BP* (95.4 hpd) and an end date of *4510-4330 cal. BP* (95.4 hpd). Interval of occupation is estimated to be 30-130 years (68.3 hpd) and 10-240 (95.3 hpd).

**Table S93:** 38CH1781AM1

**Table S94:** 38CH1781AM2

**Table S95:** 38CH1781AM3

**Table S96:** 38CH1781AM4

**Table S97:** 38CH1781AM5

***38CH1781R2 Parrot Point D***

Parrot Point D samples come from excavations described in Baluha and Poplin (2005).

Model 1 places all the dates from the test units into one Phase that contains several ordered Sequences. The order of the dates within each Sequence is the stratigraphic order that the excavators recovered each sample. The results of Model 1 indicate poor agreement. The Amodel (55.6) for the model do not indicate good agreement, i.e., exceeding the 60-threshold (see Table S98). However, the Aoverall (60.5) does exceed the 60-threshold.

Model 2 is the same as Model 1, however for this one we apply a General Outlier model to all the dates. The results of Model 2 indicate good agreement and do not identify an outlier. The Amodel (60.7) for the model indicates good agreement, i.e., exceeding the 60-threshold (see Table S99). However, the model identifies UGAMS 61235 as an outlier with poor agreement with the overall model (A=44.9). The model estimates a start date for the ring of *4520-4420 cal. BP* (68.3 hpd) and an end date of *4510-4400 cal. BP* (68.3 hpd) and a start date for the ring of *4560-4410 cal. BP* (95.4 hpd) and an end date of *4510-4360 cal. BP* (95.4 hpd).

Model 3 is identical to Model 2 in structure except that we apply a KDE command with a LnN(ln(125), ln (2) to summarize the probability distribution, which places emphasis on the earlier part of the distribution rather than later (i.e., < 125 years) (see above earlier discussion). The results of Model 5 indicate good agreement. The Amodel (69.1) for the model indicates good agreement, exceeding the 60-threshold (see Table S100). However, the model continues to identify UGAMS 61235 as an outlier with poor agreement with the overall model (A=36.2). The model estimates a start date for the ring of *4530-4430 cal. BP* (68.3 hpd) and an end date of *4490-4390 cal. BP* (68.3 hpd) and a start date for the ring of *4560-4420 cal. BP* (95.4 hpd) and an end date of *4500-4360 cal. BP* (95.4 hpd). Interval of occupation is estimated to be 10-100 years (68.3 hpd) and 10-160 (95.3 hpd).

**Table S98:** 38CH1781DM1

**Table S99:** 38CH1781DM2

**Table S100:** 38CH1781DM3

***38CH2533R1 Pockoy Ring 1***

Pockoy Island Ring 1 samples come from unit excavations and shovel tests that are currently undergoing analysis at the Heritage Trust Program, South Carolina Department of Natural Resources. This work is briefly described in Smith et al. (2021).

Model 1 places all the dates from the shovel test and excavation units into a single Phase that contains multiple sequences. Four samples were in sequence within their respective quadrats. The remaining AMS samples do not share demonstrable stratigraphic relationships to one another; although it may be possible to aggregate contexts into broad stratigraphic groups that cross quadrats in future models. One OSL sample taken from the base of the ring midden deposit in Quadrat 2 provides a TPQ for the Ring 1 models. The results of Model 1 indicate good agreement. The Amodel (94) for the model indicates good agreement, exceeding the 60-threshold (see Table S101). The model estimates a start date for the ring of *4370-4250 cal. BP* (68.3 hpd) and an end date of *4250-4160 cal. BP* (68.3 hpd) and a start date for the ring of *4430-4240 cal. BP* (95.4 hpd) and an end date of *4290-4120 cal. BP* (95.4 hpd).

Model 2 is the same as Model 1, however for this one we apply a General Outlier model to all the dates. The results of Model 2 indicate good agreement and do not identify an outlier. The Amodel (96.2) for the model indicates good agreement, i.e., exceeding the 60-threshold (see Table S102). The model estimates a start date for the ring of *4350-4250 cal. BP* (68.3 hpd) and an end date of *4250-4170 cal. BP* (68.3 hpd) and a start date for the ring of *4420-4240 cal. BP* (95.4 hpd) and an end date of *4290-4130 cal. BP* (95.4 hpd).

Model 3 is identical to Model 2 in structure except that we apply a KDE command with a LnN(ln(125), ln (2) to summarize the probability distribution, which places emphasis on the earlier part of the distribution rather than later (i.e., < 125 years) (see above earlier discussion). The results of Model 5 indicate good agreement. The Amodel (94.5) for the model indicates good agreement, exceeding the 60-threshold (see Table S103). The model estimates a start date for the ring of *4330-4250 cal. BP* (68.3 hpd) and an end date of *4280-4199 cal. BP* (68.3 hpd) and a start date for the ring of *4390-4240 cal. BP* (95.4 hpd) and an end date of *4290-4150 cal. BP* (95.4 hpd). Interval of occupation is estimated to be 0-120 years (68.3 hpd) and 0-210 (95.3 hpd).

**Table S101:** 38CH2533R1M1

**Table S102:** 38CH2533R1M2

**Table S103:** 38CH2533R1M3

***38CH2533R2 Pockoy Ring 2***

Pockoy Island Ring 2 samples come from a unit excavation and shovel tests described in Smith et al. (2019).

Model 1 places all the dates from the shovel test units into one Phase and are in stratigraphic order. The results of Model 1 indicate good agreement. The Amodel (84.9) for the model indicates good agreement, exceeding the 60-threshold (see Table S104). The model estimates a start date for the ring of *4330-4190 cal. BP* (68.3 hpd) and an end date of *4150-4060 cal. BP* (68.3 hpd) and a start date for the ring of *4430-4170 cal. BP* (95.4 hpd) and an end date of *4150-3930 cal. BP* (95.4 hpd).

Model 2 is the same as Model 1, however for this one we apply a General Outlier model to all the dates. The results of Model 2 indicate good agreement and do not identify an outlier. The Amodel (88.6) for the model indicates good agreement, i.e., exceeding the 60-threshold (see Table S105). The model estimates a start date for the ring of *4330-4190 cal. BP* (68.3 hpd) and an end date of *4250-4060 cal. BP* (68.3 hpd) and a start date for the ring of *4430-4160 cal. BP* (95.4 hpd) and an end date of *4150-3930 cal. BP* (95.4 hpd).

Model 3 is identical to Model 1 in structure except that we apply a KDE command with a LnN(ln(125), ln (2) to summarize the probability distribution, which places emphasis on the earlier part of the distribution rather than later (i.e., < 125 years) (see above earlier discussion). The results of Model 5 indicate good agreement. The Amodel (82.2) for the model indicates good agreement, exceeding the 60-threshold (see Table S106). However, the model identifies OxA - 41062-N2140E1020 as an outlier with poor agreement with the overall model (A=57.4). The model estimates a start date for the ring of *4300-4180 cal. BP* (68.3 hpd) and an end date of *4150-4090 cal. BP* (68.3 hpd) and a start date for the ring of *4350-4160 cal. BP* (95.4 hpd) and an end date of *4150-4020 cal. BP* (95.4 hpd). Interval of occupation is estimated to be 60-190 years (68.3 hpd) and 30-300 (95.3 hpd).

**Table S104:** 38CH2533R2M1

**Table S105:** 38CH2533R2M2

**Table S106:** 38CH2533R2M3

**A Comparison of all 25 shell rings/mounds in the study area**

In order to compare the dates from each ring, we saved the KDE plot estimate for the duration of occupation Prior from each model for the date estimate for each shell ring were then saved in OxCal (see Manning et al. 2022). We then placed all these in a single phase with the Order command. The Order command is used to determine the relative order of events within the program. In this case we took the posterior probability for the duration of each of the rings to assess the chronological relationship between each of the rings. Table S105 presents the probabilities regarding these relationships.

**Table S107. The Order of each shell ring and mound in the study area based on the KDE distribution.**

**References Cited**

Baluha, D. S., Poplin, E. C. (2005). Archaeological Data Recovery at 38CH1781 Charleston County, South Carolina. Brockington and Associates, Inc. Draft Report Prepared for Ford Development Corporation Dallas, Texas.

Beasley, Dana. (1970). Archaeological Investigations on Skidaway Island, Chatham County, Georgia. Proposal to the Branigar Organization. Ms. on file, South Carolina Institute of Archaeology and Anthropology, Columbia.

Bragg, Laura M. (1925) An Indian Shell Culture in South Carolina. The Charleston Museum Quarterly 1(2):3–7.

Bronk Ramsey, C. (1995). Radiocarbon calibration and analysis of stratigraphy: the OxCal program. *Radiocarbon*, *37*(2), 425-430.

Bronk Ramsey, C. (2009). Bayesian analysis of radiocarbon dates. *Radiocarbon*, *51*(1), 337-360.

Buck, C. E., Kenworthy, J. B., Litton, C. D., & Smith, A. F. (1991). Combining archaeological and radiocarbon information: a Bayesian approach to calibration. *Antiquity*, *65*(249), 808-821.

Cajigas, Rachel, Matthew C. Sanger, Anna Semon, Victor Thompson, Carey James Garland, Elliot H. Blair, David Hurst Thomas. (2024, in review). Sequential Villages and Settling Down on the Southeast U.S. Coast. Frontiers in Human Dynamics, Institutions and Collective Action.

Calmes, Alan. (1967) Test Excavations at Two Late Archaic Sites on Hilton Head Island, Beaufort County, South Carolina. Paper presented at the Southeastern Archaeological Conference, Macon, Georgia, Nov. 9, 1967.

Crook, M.R. Jr. (2009). Bilbo (9CH4) and Delta (38JA23): late archaic and Early Woodland shell mounds at the mouth of the Savannah River. *Occasional Papers in Cultural Resrource Management* 317:1-105.

Crusoe, Donald L., and Chester B. DePratter. (1976). A New Look at the Georgia Shell Mound Archaic. The Florida Anthropologist 29(1):1–23.

DePratter, Chester (1991) W.P.A. Archaeological Excavations in Chatham County, Georgia, 1937–1942. University of Georgia Laboratory of Archaeology Series Report Number 29, Athens.

DePratter, Chester (1976) The Shellmound Archaic on the Georgia Coast. Master’s thesis. University of Georgia, Athens.

DePratter, Chester (1975). An Archaeological Survey of the P. H. Lewis Property on Skidaway Island, Chatham County, Georgia. Manuscript on file, University of Georgia, Laboratory of Archaeology (Research Manuscript 343).

DePratter, Chester (1974). An Archaeological Survey of Ossabaw Island, Chatham County, Georgia: Preliminary Report. Ms. on file (Research Manuscript 344), University of Georgia, Laboratory of Archaeology, Athens.

Dorroh, Rita J. (1971) The Vegetation of Indian Shell Mounds and Rings of the South Carolina Coast. Master’s thesis, Department of Biology, University of South Carolina, Columbia.

Drayton, John (1802) A View of South Carolina, as Respects Her Natural and Civil Concerns. W. P. Young, Charleston.

Flannery, Regina (1943) Some Notes on a Few Sites in Beaufort County, South Carolina. Anthropological Papers No. 21. From Bureau of American Ethnology Bulletin 133, pp. 143–153. Smithsonian Institution, Washington.

Garland C.J.**,** Thompson V.D., Gragson T., Demyan M., Parbus B., Howland M.D., Andrus C.F.T. (Under Review) New evidence from the *Hokfv-Mocvse* Shell Ring (5000-4800 cal. BP) on the emergence of preceramic sedentary villages on South Atlantic Coast of Georgia. Submitted to *American Antiquity*

Hamilton, W. D., & Krus, A. M. (2018). The myths and realities of Bayesian chronological modeling revealed. *American Antiquity*, *83*(2), 187-203.

Heide, G., & Russo, M. (2003). Investigation of the Coosaw Island Shell Ring Complex (38BU1866). Southeast Archeological Center, National Park Service.

Heide, Gregory (2002) Mapping of the Fig Island Shell Rings. In The Fig Island Ring Complex (38CH42): Coastal Adaptation and the Question of Ring Function in the Late Archaic, by Rebecca Saunders and Michael Russo, pp. 67–84. Grant No. 45-01-16441, South Carolina Department of Archives and History, Columbia.

Hemmings, E. Thomas. (1970a) Buzzard’s Island (38CH23). National Register of Historic Places Inventory–Nomination Form. National Park Service, Washington.

Hemmings, E. Thomas. (1970b) Hanckel Mound (38CH7). National Register of Historic Places Inventory–Nomination Form. National Park Service, Washington.

Hemmings, E. Thomas. (1970c) Horse Island (38Ch14). National Register of Historic Places Inventory–Nomination Form. National Park Service, Washington.

Hemmings, E. Thomas. (1970d) Auld Mound or Yough Hall Plantation Shell Ring (38CH41). National Register of Historic Places Inventory–Nomination Form. National Park Service, Washington.

Hemmings, E. Thomas. (1970e) Fig Island (38CH42). National Register of Historic Places Inventory–Nomination Form. National Park Service, Washington.

Hemmings, E. Thomas. (1970f) Preliminary Report of Excavations at Fig Island, South Carolina. Notebook 2(9):9–15. Institute of Archaeology and Anthropology, University of South Carolina, Columbia.

Hemmings, E. Thomas. (1970g) Sewee Shell Ring (38CH45). National Register of Historic Places Inventory–Nomination Form. National Park Service, Washington.

Howard, James D., and Chester B. DePratter. (1980). Field Trip Guide to the Archaeology-Geology of the Georgia Coast. In Excursions in Southeastern Geology: The Archaeology-Geology of the Georgia Coast (Guidebook 20), edited by James D. Howard, Chester B. DePratter, and Robert W. Frey, pp. 234–253. Geological Society of America, Georgia Geological Survey, Atlanta.

Judge, Christopher, and Steven D. Smith (1991) Acquiring the Past for the Future: The South Carolina Heritage Trust Statewide Assessment of Cultural Sites. Research Manuscript Series 213, South Carolina Institute of Archaeology and Anthropology, Columbia.

Lulewicz, I. H., Thompson, V. D., Cramb, J., & Tucker, B. (2017). Oyster paleoecology and native American subsistence practices on Ossabaw Island, Georgia, USA. *Journal of Archaeological Science: Reports*, *15*, 282-289.

Manning, S. W., & Birch, J. (2022). A centennial ambiguity: The challenge of resolving the date of the Jean-Baptiste Lainé (Mantle), Ontario, site—around AD 1500 or AD 1600?—and the case for wood-charcoal as a terminus post quem. *Radiocarbon*, *64*(2), 279-308.

Marrinan, Rochelle. (1975) Ceramics, Molluscs, and Sedentism: The Late Archaic Period on the Georgia Coast. Ph.D. dissertation, Department of Anthropology, University of Florida, Gainesville.

Marquardt, W. H., Krus, A. M., & Thompson, V. D. (2020). Rethinking the Estero Island site: A possible satellite village of Mound Key. *Journal of Anthropological Archaeology*, *58*, 101145.

Marquardt, W. H., Walker, K. J., Thompson, V. D., Savarese, M., Thompson, A. D. R., & Newsom, L. A. (2022). Episodic complexity and the emergence of a coastal kingdom: Climate, cooperation, and coercion in Southwest Florida. *Journal of Anthropological Archaeology*, *65*, 101364.

McKinley, William. (1873) Mounds in Georgia. Smithsonian Annual Report for 1872, 27:422–428.

Moore, Clarence B. (1898) Certain Aboriginal Mounds of the Coast of South Carolina. Journal of the Academy of Natural Sciences of Philadelphia 11:147–166.

Reimer, P. J., Austin, W. E., Bard, E., Bayliss, A., Blackwell, P. G., Ramsey, C. B., ... & Talamo, S. (2020). The IntCal20 Northern Hemisphere radiocarbon age calibration curve (0–55 cal kBP). *Radiocarbon*, *62*(4), 725-757.

Ritter, Woldemar H. (1933) Shell Mound on Land Owned by Chester Fields, Burton, Port Royal Island, Beaufort County, South Carolina. Plan of Mound and Location of Test Pits. Map and associated field notes on file at the Charleston Museum, South Carolina.

Russo, M. (2006). *Archaic shell rings of the Southeast US*. Southeast Archeological Center, National Park Service.

Russo, M. (2002). Architectural Features at Fig Island. In The Fig Island Ring Complex (38CH42): Coastal Adaptation and the Question of Ring Function in the Late Archaic, by Rebecca Saunders and Michael Russo, pp. 69–72. Grant 45-01-16441, South Carolina Department of Archives and History, Columbia.

Sanger, M.C. (2015). Life in the Round: Shell Rings of the Georgia Bight. Ph.D. Dissertation, Columbia University.

Saunders, R., editor. (2002). The Fig Island Ring Complex (38CH42): Coastal Adaptation and the Question of Ring Function in the Late Archaic. Report prepared for the South Carolina Department of Archives and History under grant #45-01-16441.

Saunders, Rebecca, Michael Russo, and Virginia Horak. (2006) Daws Island Project-2006. Poster presented at the annual meeting of the Archaeological Society of South Carolina, Columbia.

Smith, K. Y. (2023). Letter Report for Godfrey’s Privet Planting Holes, South Bluff Heritage Preserve, Beaufort County, South Carolina. Report on file with the Heritage Trust Program, South Carolina Department of Natural Resources.

Smith, K. Y., Gaillard, M., and Taylor, T. (2021). Pockoy Island Shell Ring 1 Management Summary. Report on file with the Heritage Trust Program, South Carolina Department of Natural Resources.

Smith, K. Y., Taylor, T., Ghaffar, T., Gaillard, M., and Arrington, T. (2019). An Archaeological Survey of Pockoy Island, Botany Bay Plantation Heritage Preserve, Charleston County, South Carolina. Report on file with the Heritage Trust Program, South Carolina Department of Natural Resources.

Sutherland, Donald R. (1974). Excavations at the Spanish Mount Shell Midden, Edisto Island, South Carolina. South Carolina Antiquities 6(1):25–36

Thompson, Victor D. (2006) Questioning Complexity: The Prehistoric Hunter-Gatherers of Sapelo Island, Georgia. Ph.D. Dissertation, University of Kentucky, Lexington.

Trinkley, M.B. (1980). *Investigation of the Woodland Period Along the South Carolina Coast*. Ph.D. dissertation, Department of Anthropology, University of North Carolina, Chapel Hill.

Trinkley, M.B. (1985). The Form and Function of South Carolina’s Early Woodland Shell Rings. In Structure and Process in Southeastern Archaeology, edited by Roy S. Dickens Jr. and H. Trawick Ward, pp. 102–118. University of Alabama Press, Tuscaloosa.

Waring, Antonio. (1968). The Archaic Hunting and Gathering Cultures. The Archaic and Some Shell Rings. In The Waring Papers: The Collected Works of Antonio J. Waring, Jr., edited by Stephen Williams, pp. 243–246. Papers of the Peabody Museum of Archaeology and Ethnology 58. Harvard University, Cambridge.

Waring, Antonio J., Jr., and Lewis H. Larson. (1968). The Shell Ring on Sapelo Island. In The Waring Papers: The Collected Works of Antonio J. Waring, Jr., edited by Stephen Williams, pp. 263–278. Papers of the Peabody Museum of Archaeology and Ethnology 58, Harvard University, Cambridge.

**INDIVIDUAL MODEL RUNFILES**

Below are the runfiles for each individual model. Each runfile is labeled by site number, ring number (e.g., R1), and model number (e.g., M1).

***9LI2169R1M1***

*Plot()*

*{*

*Sequence(9LI2169)*

*{*

*Boundary("Start: 9LI2169");*

*Phase("9LI1648 Units")*

*{*

*Sequence("N2093 E4176")*

*{*

*R_Date("UGAMS 65147",3820,25);*

*R_Date("UGAMS 65148",3800,25);*

*R_Date("UGAMS 65149", 3840, 25)*

*{*

*};*

*R_Date("UGAMS 65150",3860,25);*

*};*

*Sequence("N2131 E4186")*

*{*

*R_Date("UGAMS 65158",3950,25)*

*{*

*Outlier();*

*};*

*R_Date("UGAMS 65157",3840,25);*

*R_Date("UGAMS 65161",4060,25);*

*R_Date("UGAMS 65160",3860,25);*

*R_Date("UGAMS 65159",3860,25);*

*R_Date("UGAMS 65156",3820,25);*

*};*

*Sequence("N2221 E4209")*

*{*

*R_Date("UGAMS 65155",3910,25);*

*R_Date("UGAMS 65153",3810,25);*

*R_Date("UGAMS 65152",3910,25);*

*R_Date("UGAMS 65151",3900,25);*

*};*

*};*

*Boundary("End: 9LI2169");*

*};*

*Difference("Lapse","Start: 9LI2169","End: 9LI2169");*

*};*

***9LI2169R1M2***

*Plot()*

*{*

*Sequence(9LI2169)*

*{*

*Boundary("Start: 9LI2169");*

*Phase("9LI1648 Units")*

*{*

*Sequence("N2093 E4176")*

*{*

*R_Date("UGAMS 65147",3820,25);*

*R_Date("UGAMS 65148",3800,25);*

*R_Date("UGAMS 65150",3860,25);*

*};*

*Sequence("N2131 E4186")*

*{*

*R_Date("UGAMS 65157",3840,25);*

*R_Date("UGAMS 65161",4060,25);*

*R_Date("UGAMS 65160",3860,25);*

*R_Date("UGAMS 65159",3860,25);*

*R_Date("UGAMS 65156",3820,25);*

*};*

*Sequence("N2221 E4209")*

*{*

*R_Date("UGAMS 65152",3910,25);*

*R_Date("UGAMS 65151",3900,25);*

*};*

*Phase("Pre-Shell Deposit Layers")*

*{*

*R_Date("UGAMS 65149", 3840, 25)*

*{*

*};*

*R_Date("UGAMS 65155",3910,25);*

*R_Date("UGAMS 65153",3810,25);*

*R_Date("UGAMS 65158",3950,25)*

*{*

*};*

*};*

*};*

*Boundary("End: 9LI2169");*

*};*

*Difference("Lapse","Start: 9LI2169","End: 9LI2169");*

*};*

***9LI2169R1M3***

*Plot()*

*{*

*Sequence(9LI2169)*

*{*

*Boundary("Start: 9LI2169");*

*Phase("9LI1648 Units")*

*{*

*Sequence("N2093 E4176")*

*{*

*R_Date("UGAMS 65147",3820,25);*

*R_Date("UGAMS 65148",3800,25);*

*R_Date("UGAMS 65150",3860,25);*

*};*

*Phase("N2131 E4186")*

*{*

*R_Date("UGAMS 65157",3840,25);*

*R_Date("UGAMS 65161",4060,25);*

*R_Date("UGAMS 65160",3860,25);*

*R_Date("UGAMS 65159",3860,25);*

*R_Date("UGAMS 65156",3820,25);*

*};*

*Sequence("N2221 E4209")*

*{*

*R_Date("UGAMS 65152",3910,25);*

*R_Date("UGAMS 65151",3900,25);*

*};*

*Phase("Pre-Shell Deposit Layers")*

*{*

*R_Date("UGAMS 65149", 3840, 25)*

*{*

*};*

*R_Date("UGAMS 65155",3910,25);*

*R_Date("UGAMS 65153",3810,25);*

*R_Date("UGAMS 65158",3950,25)*

*{*

*};*

*};*

*};*

*Boundary("End: 9LI2169");*

*};*

*Difference("Lapse","Start: 9LI2169","End: 9LI2169");*

*};*

***9LI2169R1M4***

*Plot()*

*{*

*Outlier_Model("Charcoal",Exp(1,-10,0),U(0,3),"t");*

*Outlier_Model("General",T(5),U(0,4),"t");*

*Sequence(9LI2169)*

*{*

*Boundary("Start: 9LI2169");*

*Phase("9LI1648 Units")*

*{*

*Sequence("N2093 E4176")*

*{*

*R_Date("UGAMS 65147",3820,25)*

*{*

*Outlier("Charcoal",1);*

*};*

*R_Date("UGAMS 65148",3800,25)*

*{*

*Outlier("General",0.01);*

*};*

*R_Date("UGAMS 65150",3860,25)*

*{*

*Outlier("Charcoal",1);*

*};*

*};*

*Phase("N2131 E4186")*

*{*

*R_Date("UGAMS 65157",3840,25)*

*{*

*Outlier("Charcoal",1);*

*};*

*R_Date("UGAMS 65161",4060,25)*

*{*

*Outlier("Charcoal",1);*

*};*

*R_Date("UGAMS 65160",3860,25)*

*{*

*Outlier("Charcoal",1);*

*};*

*R_Date("UGAMS 65159",3860,25)*

*{*

*Outlier("Charcoal",1);*

*};*

*R_Date("UGAMS 65156",3820,25)*

*{*

*Outlier("Charcoal",1);*

*};*

*};*

*Sequence("N2221 E4209")*

*{*

*R_Date("UGAMS 65152",3910,25)*

*{*

*Outlier("Charcoal",1);*

*};*

*R_Date("UGAMS 65151",3900,25)*

*{*

*Outlier("Charcoal",1);*

*};*

*};*

*Phase("Pre-Shell Deposit Layers")*

*{*

*R_Date("UGAMS 65149", 3840, 25)*

*{*

*Outlier("General",0.01);*

*};*

*R_Date("UGAMS 65155",3910,25)*

*{*

*Outlier("Charcoal",1);*

*};*

*R_Date("UGAMS 65153",3810,25)*

*{*

*Outlier("General",0.01);*

*};*

*R_Date("UGAMS 65158",3950,25)*

*{*

*Outlier("Charcoal",1);*

*};*

*Boundary("End: 9LI2169");*

*};*

*Difference("Lapse","Start: 9LI2169","End: 9LI2169");*

*};*

***9LI2169R1M5***

*Plot()*

*{*

*Sequence(9LI2169)*

*{*

*Outlier_Model("General",T(5),U(0,4),"t");*

*Boundary("Start: 9LI2169");*

*Phase("9LI1648 Units")*

*{*

*Sequence("N2093 E4176")*

*{*

*R_Date("UGAMS 65147",3820,25)*

*{*

*Outlier("General",0.01);*

*};*

*R_Date("UGAMS 65148",3800,25)*

*{*

*Outlier("General",0.01);*

*};*

*R_Date("UGAMS 65150",3860,25)*

*{*

*Outlier("General",0.01);*

*};*

*};*

*Phase("N2131 E4186")*

*{*

*R_Date("UGAMS 65157",3840,25)*

*{*

*Outlier("General",0.01);*

*};*

*R_Date("UGAMS 65161",4060,25)*

*{*

*Outlier("General",0.01);*

*};*

*R_Date("UGAMS 65160",3860,25)*

*{*

*Outlier("General",0.01);*

*};*

*R_Date("UGAMS 65159",3860,25)*

*{*

*Outlier("General",0.01);*

*};*

*R_Date("UGAMS 65156",3820,25)*

*{*

*Outlier("Charcoal",1);*

*};*

*};*

*Sequence("N2221 E4209")*

*{*

*R_Date("UGAMS 65152",3910,25)*

*{*

*Outlier("General",0.01);*

*};*

*R_Date("UGAMS 65151",3900,25)*

*{*

*Outlier("General",0.01);*

*};*

*};*

*Phase("Pre-Shell Deposit Layers")*

*{*

*R_Date("UGAMS 65149", 3840, 25)*

*{*

*Outlier("General",0.01);*

*};*

*R_Date("UGAMS 65155",3910,25)*

*{*

*Outlier("General",0.01);*

*};*

*R_Date("UGAMS 65153",3810,25)*

*{*

*Outlier("General",0.01);*

*};*

*R_Date("UGAMS 65158",3950,25)*

*{*

*Outlier("General",0.01);*

*};*

*};*

*};*

*Boundary("End: 9LI2169");*

*};*

*Difference("Lapse","Start: 9LI2169","End: 9LI2169");*

*};*

***9LI2169R1M6***

*Plot()*

*{*

*Outlier_Model("Charcoal",Exp(1,-10,0),U(0,3),"t");*

*Outlier_Model("General",T(5),U(0,4),"t");*

*Sequence(9LI2169)*

*{*

*Boundary("Start: 9LI2169");*

*KDE_Plot("9LI2169 Units")*

*{*

*Sequence("N2093 E4176")*

*{*

*R_Date("UGAMS 65147",3820,25)*

*{*

*Outlier("Charcoal",1);*

*};*

*R_Date("UGAMS 65148",3800,25)*

*{*

*Outlier("General",0.01);*

*};*

*R_Date("UGAMS 65150",3860,25)*

*{*

*Outlier("Charcoal",1);*

*};*

*};*

*Phase("N2131 E4186")*

*{*

*R_Date("UGAMS 65157",3840,25)*

*{*

*Outlier("Charcoal",1);*

*};*

*R_Date("UGAMS 65161",4060,25)*

*{*

*Outlier("Charcoal",1);*

*};*

*R_Date("UGAMS 65160",3860,25)*

*{*

*Outlier("Charcoal",1);*

*};*

*R_Date("UGAMS 65159",3860,25)*

*{*

*Outlier("Charcoal",1);*

*};*

*R_Date("UGAMS 65156",3820,25)*

*{*

*Outlier("Charcoal",1);*

*};*

*};*

*Sequence("N2221 E4209")*

*{*

*R_Date("UGAMS 65152",3910,25)*

*{*

*Outlier("Charcoal",1);*

*};*

*R_Date("UGAMS 65151",3900,25)*

*{*

*Outlier("Charcoal",1);*

*};*

*};*

*Phase("Pre-Shell Deposit Layers")*

*{*

*R_Date("UGAMS 65149", 3840, 25)*

*{*

*Outlier("General",0.01);*

*};*

*R_Date("UGAMS 65155",3910,25)*

*{*

*Outlier("Charcoal",1);*

*};*

*R_Date("UGAMS 65153",3810,25)*

*{*

*Outlier("General",0.01);*

*};*

*R_Date("UGAMS 65158",3950,25)*

*{*

*Outlier("Charcoal",1);*

*};*

*};*

*Interval(LnN(ln(125),ln(2)));*

*};*

*Boundary("End: 9LI2169");*

*};*

*Difference("Lapse","Start: 9LI2169","End: 9LI2169");*

*};*

***9LI1648R1M1***

*Plot()*

*{*

*Sequence(9LI1648)*

*{*

*Boundary("Start: 9LI1648");*

*Phase("9LI1648 Units")*

*{*

*Sequence("N243 E233")*

*{*

*R_Date("Beta-251761", 3720, 40)*

*{*

*};*

*Phase("LVL 4.4 to 4.3")*

*{*

*R_Date("Beta-251767", 3680, 40)*

*{*

*};*

*R_Date("UCIAMS-87904", 3730, 20)*

*{*

*};*

*};*

*};*

*Sequence("N272 E200")*

*{*

*R_Date("Beta-251764", 3710, 40)*

*{*

*};*

*R_Date("Beta-251766", 3800, 40)*

*{*

*};*

*};*

*Phase("N243 E234")*

*{*

*R_Date("UCIAMS-87903", 3685, 20)*

*{*

*};*

*R_Date("UCIAMS-84269", 3620, 20)*

*{*

*};*

*R_Date("UCIAMS-84270", 3640, 20)*

*{*

*};*

*};*

*Phase("Single Dates")*

*{*

*R_Date("UCIAMS-87905", 3690, 20)*

*{*

*};*

*R_Date("Beta-244620", 3800, 40)*

*{*

*};*

*};*

*};*

*Boundary("End: 9LI1648");*

*};*

*Difference("Lapse","Start: 9LI1648","End: 9LI1648");*

*};*

***9LI1648R1M2***

*Plot()*

*{*

*Sequence(9LI1648)*

*{*

*Boundary("Start: 9LI1648");*

*Phase("9LI1648 Units")*

*{*

*Sequence("N243 E233")*

*{*

*R_Date("Beta-251761", 3720, 40)*

*{*

*};*

*Phase("LVL 4.4 to 4.3")*

*{*

*R_Date("Beta-251767", 3680, 40)*

*{*

*};*

*R_Date("UCIAMS-87904", 3730, 20)*

*{*

*};*

*};*

*};*

*Sequence("N272 E200")*

*{*

*R_Date("Beta-251764", 3710, 40)*

*{*

*};*

*R_Date("Beta-251766", 3800, 40)*

*{*

*};*

*};*

*Phase("N243 E234")*

*{*

*R_Date("UCIAMS-87903", 3685, 20)*

*{*

*};*

*R_Date("UCIAMS-84269", 3620, 20)*

*{*

*};*

*R_Date("UCIAMS-84270", 3640, 20)*

*{*

*};*

*};*

*Phase("Single Dates")*

*{*

*R_Date("UCIAMS-87905", 3690, 20)*

*{*

*};*

*R_Date("Beta-244620", 3800, 40)*

*{*

*Outlier();*

*};*

*};*

*};*

*Boundary("End: 9LI1648");*

*};*

*Difference("Lapse","Start: 9LI1648","End: 9LI1648");*

*};*

***9LI1648R1M3***

*Plot()*

*{*

*Outlier_Model("General",T(5),U(0,4),"t");*

*Sequence(9LI1648)*

*{*

*Boundary("Start: 9LI1648");*

*Phase("9LI1648 Units")*

*{*

*Sequence("N243 E233")*

*{*

*R_Date("Beta-251761", 3720, 40)*

*{*

*Outlier("General", 0.05);*

*};*

*Phase("LVL 4.4 to 4.3")*

*{*

*R_Date("Beta-251767", 3680, 40)*

*{*

*Outlier("General", 0.05);*

*};*

*R_Date("UCIAMS-87904", 3730, 20)*

*{*

*Outlier("General", 0.05);*

*};*

*};*

*};*

*Sequence("N272 E200")*

*{*

*R_Date("Beta-251764", 3710, 40)*

*{*

*Outlier("General", 0.05);*

*};*

*R_Date("Beta-251766", 3800, 40)*

*{*

*Outlier("General",0.05);*

*};*

*};*

*Phase("N243 E234")*

*{*

*R_Date("UCIAMS-87903", 3685, 20)*

*{*

*Outlier("General", 0.05);*

*};*

*R_Date("UCIAMS-84269", 3620, 20)*

*{*

*Outlier("General", 0.05);*

*};*

*R_Date("UCIAMS-84270", 3640, 20)*

*{*

*Outlier("General", 0.05);*

*};*

*};*

*Phase("Single Dates")*

*{*

*R_Date("UCIAMS-87905", 3690, 20)*

*{*

*Outlier("General", 0.05);*

*};*

*};*

*};*

*Boundary("End: 9LI1648");*

*};*

*Difference("Lapse","Start: 9LI1648","End: 9LI1648");*

*};*

***9LI1648R1M4***

*Plot()*

*{*

*Outlier_Model("Charcoal",Exp(1,-10,0),U(0,3),"t");*

*Sequence(9LI1648)*

*{*

*Boundary("Start: 9LI1648");*

*Phase("9LI1648 Units")*

*{*

*Sequence("N243 E233")*

*{*

*R_Date("Beta-251761", 3720, 40)*

*{*

*Outlier("Charcoal", 1);*

*};*

*Phase("LVL 4.4 to 4.3")*

*{*

*R_Date("Beta-251767", 3680, 40)*

*{*

*Outlier("Charcoal", 1);*

*};*

*R_Date("UCIAMS-87904", 3730, 20)*

*{*

*Outlier("Charcoal", 1);*

*};*

*};*

*};*

*Sequence("N272 E200")*

*{*

*R_Date("Beta-251764", 3710, 40)*

*{*

*Outlier("Charcoal", 1);*

*};*

*};*

*Phase("N243 E234")*

*{*

*R_Date("UCIAMS-87903", 3685, 20)*

*{*

*};*

*R_Date("UCIAMS-84269", 3620, 20)*

*{*

*Outlier("Charcoal", 1);*

*};*

*R_Date("UCIAMS-84270", 3640, 20)*

*{*

*Outlier("Charcoal", 1);*

*};*

*};*

*Phase("Single Dates")*

*{*

*R_Date("UCIAMS-87905", 3690, 20)*

*{*

*};*

*};*

*};*

*Boundary("End: 9LI1648");*

*};*

*Difference("Lapse","Start: 9LI1648","End: 9LI1648");*

*};*

***9LI1648R1M5***

*Plot()*

*{*

*Outlier_Model("Charcoal",Exp(1,-10,0),U(0,3),"t");*

*Outlier_Model("General",T(5),U(0,4),"t");*

*Sequence(9LI1648)*

*{*

*Boundary("Start: 9LI1648");*

*Phase("9LI1648 Units")*

*{*

*Sequence("N243 E233")*

*{*

*R_Date("Beta-251761", 3720, 40)*

*{*

*Outlier("Charcoal", 1);*

*};*

*Phase("LVL 4.4 to 4.3")*

*{*

*R_Date("Beta-251767", 3680, 40)*

*{*

*Outlier("Charcoal", 1);*

*};*

*R_Date("UCIAMS-87904", 3730, 20)*

*{*

*Outlier("General", 0.05);*

*};*

*};*

*};*

*Sequence("N272 E200")*

*{*

*R_Date("Beta-251764", 3710, 40)*

*{*

*Outlier("Charcoal", 1);*

*};*

*};*

*Phase("N243 E234")*

*{*

*R_Date("UCIAMS-87903", 3685, 20)*

*{*

*Outlier("General", 0.05);*

*};*

*R_Date("UCIAMS-84269", 3620, 20)*

*{*

*Outlier("Charcoal", 1);*

*};*

*R_Date("UCIAMS-84270", 3640, 20)*

*{*

*Outlier("Charcoal", 1);*

*};*

*};*

*Phase("Single Dates")*

*{*

*R_Date("UCIAMS-87905", 3690, 20)*

*{*

*Outlier("General", 0.05);*

*};*

*};*

*};*

*Boundary("End: 9LI1648");*

*};*

*Difference("Lapse","Start: 9LI1648","End: 9LI1648");*

*};*

***9LI1648R1M6***

*Plot()*

*{*

*Outlier_Model("General",T(5),U(0,4),"t");*

*Sequence(9LI1648)*

*{*

*Boundary("Start: 9LI1648");*

*KDE_Plot("9LI1648 Units")*

*{*

*Sequence("N243 E233")*

*{*

*R_Date("Beta-251761", 3720, 40)*

*{*

*Outlier("General", 0.05);*

*};*

*Phase("LVL 4.4 to 4.3")*

*{*

*R_Date("Beta-251767", 3680, 40)*

*{*

*Outlier("General", 0.05);*

*};*

*R_Date("UCIAMS-87904", 3730, 20)*

*{*

*Outlier("General", 0.05);*

*};*

*};*

*};*

*Sequence("N272 E200")*

*{*

*R_Date("Beta-251764", 3710, 40)*

*{*

*Outlier("General", 0.05);*

*};*

*};*

*Phase("N243 E234")*

*{*

*R_Date("UCIAMS-87903", 3685, 20)*

*{*

*Outlier("General", 0.05);*

*};*

*R_Date("UCIAMS-84269", 3620, 20)*

*{*

*Outlier("General", 0.05);*

*};*

*R_Date("UCIAMS-84270", 3640, 20)*

*{*

*Outlier("General", 0.05);*

*};*

*};*

*Phase("Single Dates")*

*{*

*R_Date("UCIAMS-87905", 3690, 20)*

*{*

*Outlier("General", 0.05);*

*};*

*};*

*Interval(LnN(ln(125),ln(2)));*

*};*

*Boundary("End: 9LI1648");*

*};*

*Difference("Lapse","Start: 9LI1648","End: 9LI1648");*

*};*

***9LI231R1M1***

*Plot()*

*{*

*Sequence()*

*{*

*Boundary("Start: 9LI231");*

*Phase("9LI231 Shell Ring Units")*

*{*

*R_Date("Beta-238327", 3820, 40);*

*R_Date("Beta-238322", 3870, 40);*

*R_Date("Beta-238328", 4120, 40)*

*{*

*};*

*R_Date("Beta-238331", 3820, 40);*

*R_Date("Beta-238332", 3880, 40);*

*R_Date("Beta-238337", 3860, 40);*

*Sequence("N789E801")*

*{*

*R_Date("UGAMS 60776", 3850, 20);*

*R_Date("UGAMS 60775", 3820, 20);*

*R_Date("UGAMS 60774", 3820, 20);*

*R_Date("UGAMS 60773", 3730, 20);*

*};*

*};*

*Boundary("End: 9LI231");*

*};*

*Difference("Lapse","Start: 9LI231","End: 9LI231");*

*};*

***9LI231R1M2***

*Plot()*

*{*

*Sequence()*

*{*

*Boundary("Start: 9LI231");*

*Phase("9LI231 Shell Ring Units")*

*{*

*R_Date("Beta-238327", 3820, 40);*

*R_Date("Beta-238322", 3870, 40);*

*R_Date("Beta-238328", 4120, 40)*

*{*

*Outlier();*

*};*

*R_Date("Beta-238331", 3820, 40);*

*R_Date("Beta-238332", 3880, 40);*

*R_Date("Beta-238337", 3860, 40);*

*Sequence("N789E801")*

*{*

*R_Date("UGAMS 60776", 3850, 20);*

*R_Date("UGAMS 60775", 3820, 20);*

*R_Date("UGAMS 60774", 3820, 20);*

*R_Date("UGAMS 60773", 3730, 20);*

*};*

*};*

*Boundary("End: 9LI231");*

*};*

*Difference("Lapse","Start: 9LI231","End: 9LI231");*

*};*

***9LI231R1M3***

*Plot()*

*{*

*Outlier_Model("General",T(5),U(0,4),"t");*

*Sequence()*

*{*

*Boundary("Start: 9LI231");*

*Phase("9LI231 Shell Ring Units")*

*{*

*R_Date("Beta-238327", 3820, 40)*

*{*

*Outlier("General", 0.01);*

*};*

*R_Date("Beta-238322", 3870, 40)*

*{*

*Outlier("General", 0.01);*

*};*

*R_Date("Beta-238331", 3820, 40)*

*{*

*Outlier("General", 0.01);*

*};*

*R_Date("Beta-238332", 3880, 40)*

*{*

*Outlier("General", 0.01);*

*};*

*R_Date("Beta-238337", 3860, 40)*

*{*

*Outlier("General", 0.01);*

*};*

*Sequence("N789E801")*

*{*

*R_Date("UGAMS 60776", 3850, 20)*

*{*

*Outlier("General", 0.01);*

*};*

*R_Date("UGAMS 60775", 3820, 20)*

*{*

*Outlier("General", 0.01);*

*};*

*R_Date("UGAMS 60774", 3820, 20)*

*{*

*Outlier("General", 0.01);*

*};*

*R_Date("UGAMS 60773", 3730, 20)*

*{*

*Outlier("General", 0.01);*

*};*

*};*

*};*

*Boundary("End: 9LI231");*

*};*

*Difference("Lapse","Start: 9LI231","End: 9LI231");*

*};*

***9LI231R1M4***

*Plot()*

*{*

*Outlier_Model("Charcoal",Exp(1,-10,0),U(0,3),"t");*

*Sequence()*

*{*

*Boundary("Start: 9LI231");*

*Phase("9LI231 Shell Ring Units")*

*{*

*R_Date("Beta-238327", 3820, 40)*

*{*

*};*

*R_Date("Beta-238322", 3870, 40)*

*{*

*};*

*R_Date("Beta-238331", 3820, 40)*

*{*

*};*

*R_Date("Beta-239276", 3930, 40)*

*{*

*Outlier("Charcoal", 1);*

*};*

*R_Date("Beta-238332", 3880, 40)*

*{*

*Outlier("Charcoal", 1);*

*};*

*R_Date("Beta-238337", 3860, 40)*

*{*

*Outlier("Charcoal", 1);*

*};*

*Sequence("N789E801")*

*{*

*R_Date("UGAMS 60776", 3850, 20)*

*{*

*};*

*R_Date("UGAMS 60775", 3820, 20)*

*{*

*};*

*R_Date("UGAMS 60774", 3820, 20)*

*{*

*};*

*R_Date("UGAMS 60773", 3730, 20)*

*{*

*};*

*};*

*};*

*Boundary("End: 9LI231");*

*};*

*Difference("Lapse","Start: 9LI231","End: 9LI231");*

*};*

***9LI231R1M5***

*Plot()*

*{*

*Outlier_Model("Charcoal",Exp(1,-10,0),U(0,3),"t");*

*Outlier_Model("General",T(5),U(0,4),"t");*

*Sequence()*

*{*

*Boundary("Start: 9LI231");*

*Phase("9LI231 Shell Ring Units")*

*{*

*R_Date("Beta-238327", 3820, 40)*

*{*

*Outlier("General", 0.01);*

*};*

*R_Date("Beta-238322", 3870, 40)*

*{*

*Outlier("General", 0.01);*

*};*

*R_Date("Beta-238331", 3820, 40)*

*{*

*Outlier("Charcoal", 1);*

*};*

*R_Date("Beta-239276", 3930, 40)*

*{*

*Outlier("Charcoal", 1);*

*};*

*R_Date("Beta-238332", 3880, 40)*

*{*

*Outlier("Charcoal", 1);*

*};*

*R_Date("Beta-238337", 3860, 40)*

*{*

*Outlier("Charcoal", 1);*

*};*

*Sequence("N789E801")*

*{*

*R_Date("UGAMS 60776", 3850, 20)*

*{*

*Outlier("General", 0.01);*

*};*

*R_Date("UGAMS 60775", 3820, 20)*

*{*

*Outlier("General", 0.01);*

*};*

*R_Date("UGAMS 60774", 3820, 20)*

*{*

*Outlier("General", 0.01);*

*};*

*R_Date("UGAMS 60773", 3730, 20)*

*{*

*Outlier("General", 0.01);*

*};*

*};*

*};*

*Boundary("End: 9LI231");*

*};*

*Difference("Lapse","Start: 9LI231","End: 9LI231");*

*};*

***9LI231R1M6***

*Plot()*

*{*

*Outlier_Model("General",T(5),U(0,4),"t");*

*Sequence()*

*{*

*Boundary("Start: 9LI231");*

*KDE_Plot("9LI231 Shell Ring Units")*

*{*

*R_Date("Beta-238327", 3820, 40)*

*{*

*Outlier("General", 0.01);*

*};*

*R_Date("Beta-238322", 3870, 40)*

*{*

*Outlier("General", 0.01);*

*};*

*R_Date("Beta-238331", 3820, 40)*

*{*

*Outlier("General", 0.01);*

*};*

*R_Date("Beta-238332", 3880, 40)*

*{*

*Outlier("General", 0.01);*

*};*

*R_Date("Beta-238337", 3860, 40)*

*{*

*Outlier("General", 0.01);*

*};*

*Sequence("N789E801")*

*{*

*R_Date("UGAMS 60776", 3850, 20)*

*{*

*Outlier("General", 0.01);*

*};*

*R_Date("UGAMS 60775", 3820, 20)*

*{*

*Outlier("General", 0.01);*

*};*

*R_Date("UGAMS 60774", 3820, 20)*

*{*

*Outlier("General", 0.01);*

*};*

*R_Date("UGAMS 60773", 3730, 20)*

*{*

*Outlier("General", 0.01);*

*};*

*};*

*Interval(LnN(ln(125),ln(2)));*

*};*

*Boundary("End: 9LI231");*

*};*

*Difference("Lapse","Start: 9LI231","End: 9LI231");*

*};*

***38BU1866R2M1***

*Plot()*

*{*

*Sequence("38BU1866 Ring 2")*

*{*

*Boundary("Start: 38BU1866 Ring 2");*

*Phase("38BU1866 Ring 2 Units")*

*{*

*R_Date("UGAMS 59260-N944E2064", 3530, 25);*

*R_Date("UGAMS 59259-N941.5E2063.5", 3370, 25);*

*};*

*Boundary("End: 38BU1866 Ring 2");*

*};*

*Difference("Lapse","Start: 38BU1866 Ring 2","End: 38BU1866 Ring 2");*

*};*

***38BU1866R2M2***

*Plot()*

*{*

*Outlier_Model("General",T(5),U(0,4),"t");*

*Sequence("38BU1866 Ring 2")*

*{*

*Boundary("Start: 38BU1866 Ring 2");*

*Phase("38BU1866 Ring 2 Units")*

*{*

*R_Date("UGAMS 59260-N944E2064", 3530, 25)*

*{*

*Outlier("General", 0.05);*

*};*

*R_Date("UGAMS 59259-N941.5E2063.5", 3370, 25)*

*{*

*Outlier("General", 0.05);*

*};*

*Interval(LnN(ln(100),ln(2)));*

*};*

*Boundary("End: 38BU1866 Ring 2");*

*};*

*Difference("Lapse","Start: 38BU1866 Ring 2","End: 38BU1866 Ring 2");*

*};*

***38BU1866R2M3***

*Plot()*

*{*

*Outlier_Model("General",T(5),U(0,4),"t");*

*Sequence("38BU1866 Ring 2")*

*{*

*Boundary("Start: 38BU1866 Ring 2");*

*KDE_Plot("38BU1866 Ring 2 Units")*

*{*

*R_Date("UGAMS 59260-N944E2064", 3530, 25)*

*{*

*Outlier("General", 0.05);*

*};*

*R_Date("UGAMS 59259-N941.5E2063.5", 3370, 25)*

*{*

*Outlier("General", 0.05);*

*};*

*Interval(LnN(ln(125),ln(2)));*

*};*

*Boundary("End: 38BU1866 Ring 2");*

*};*

*Difference("Lapse","Start: 38BU1866 Ring 2","End: 38BU1866 Ring 2");*

*};*

***38BU1866R3M1***

*Plot()*

*{*

*Sequence("38BU1866 Ring 3")*

*{*

*Boundary("Start: 38BU1866 Ring 3");*

*Phase("38BU1866 Ring 3 Units")*

*{*

*R_Date("UGAMS 63212", 3540, 20)*

*{*

*};*

*R_Date("UGAMS 63213", 3480, 20)*

*{*

*};*

*};*

*Boundary("End: 38BU1866 Ring 3");*

*};*

*Difference("Lapse","Start: 38BU1866 Ring 3","End: 38BU1866 Ring 3");*

*};*

***38BU1866R3M2***

*Plot()*

*{*

*Outlier_Model("General",T(5),U(0,4),"t");*

*Sequence("38BU1866 Ring 3")*

*{*

*Boundary("Start: 38BU1866 Ring 3");*

*Phase("38BU1866 Ring 3 Units")*

*{*

*R_Date("UGAMS 63212", 3540, 20)*

*{*

*Outlier("General", 0.05);*

*};*

*R_Date("UGAMS 63213", 3480, 20)*

*{*

*Outlier("General", 0.05);*

*};*

*};*

*Boundary("End: 38BU1866 Ring 3");*

*};*

*Difference("Lapse","Start: 38BU1866 Ring 3","End: 38BU1866 Ring 3");*

*};*

***38BU1866R3M3***

*Plot()*

*{*

*Outlier_Model("General",T(5),U(0,4),"t");*

*Sequence("38BU1866 Ring 3")*

*{*

*Boundary("Start: 38BU1866 Ring 3");*

*KDE_Plot("38BU1866 Ring 3 Units")*

*{*

*R_Date("UGAMS 63212", 3540, 20)*

*{*

*Outlier("General", 0.05);*

*};*

*R_Date("UGAMS 63213", 3480, 20)*

*{*

*Outlier("General", 0.05);*

*};*

*Interval(LnN(ln(125),ln(2)));*

*};*

*Boundary("End: 38BU1866 Ring 3");*

*};*

*Difference("Lapse","Start: 38BU1866 Ring 3","End: 38BU1866 Ring 3");*

*};*

***38BU7R1M1***

*Plot()*

*{*

*Sequence("38BU7 Ring")*

*{*

*Boundary("Start: 38BU7");*

*Phase("38BU7 Ring Units")*

*{*

*Sequence("N209E512")*

*{*

*R_Date("UGAMS 43768", 3640, 20);*

*R_Date("UGAMS 43767", 3660, 20);*

*R_Date("UGAMS 43766", 3650, 20);*

*};*

*Sequence("N212E485")*

*{*

*R_Date("UGAMS 43770", 3540, 20);*

*R_Date("UGAMS 43769", 3490, 20);*

*};*

*Sequence("N184E479")*

*{*

*R_Date("UGAMS 43772", 3620, 25);*

*R_Date("UGAMS 43771", 3650, 20);*

*};*

*Phase("N178E513")*

*{*

*R_Date("UGAMS 43776", 3570, 20);*

*R_Date("UGAMS 43775", 3650, 20);*

*};*

*};*

*Boundary("End: 38BU7");*

*};*

*Difference("Lapse","Start: 38BU7","End: 38BU7");*

*};*

***38BU7R1M2***

*Plot()*

*{*

*Outlier_Model("General",T(5),U(0,4),"t");*

*Sequence("38BU7 Ring")*

*{*

*Boundary("Start: 38BU7");*

*Phase("38BU7 Ring Units")*

*{*

*Sequence("N209E512")*

*{*

*R_Date("UGAMS 43768", 3640, 20)*

*{*

*Outlier("General", 0.05);*

*};*

*R_Date("UGAMS 43767", 3660, 20)*

*{*

*Outlier("General", 0.05);*

*};*

*R_Date("UGAMS 43766", 3650, 20)*

*{*

*Outlier("General", 0.05);*

*};*

*};*

*Sequence("N212E485")*

*{*

*R_Date("UGAMS 43770", 3540, 20)*

*{*

*Outlier("General", 0.05);*

*};*

*R_Date("UGAMS 43769", 3490, 20)*

*{*

*Outlier("General", 0.05);*

*};*

*};*

*Sequence("N184E479")*

*{*

*R_Date("UGAMS 43772", 3620, 25)*

*{*

*Outlier("General", 0.05);*

*};*

*R_Date("UGAMS 43771", 3650, 20)*

*{*

*Outlier("General", 0.05);*

*};*

*};*

*Phase("N178E513")*

*{*

*R_Date("UGAMS 43776", 3570, 20)*

*{*

*Outlier("General", 0.05);*

*};*

*R_Date("UGAMS 43775", 3650, 20)*

*{*

*Outlier("General", 0.05);*

*};*

*};*

*};*

*Boundary("End: 38BU7");*

*};*

*Difference("Lapse","Start: 38BU7","End: 38BU7");*

*};*

***38BU7R1M3***

*Plot()*

*{*

*Outlier_Model("General",T(5),U(0,4),"t");*

*Sequence("38BU7 Ring")*

*{*

*Boundary("Start: 38BU7");*

*Phase("38BU7 Ring Units")*

*{*

*Sequence("N209E512")*

*{*

*R_Date("UGAMS 43768", 3640, 20)*

*{*

*Outlier("General", 0.05);*

*};*

*R_Date("UGAMS 43767", 3660, 20)*

*{*

*Outlier("General", 0.05);*

*};*

*R_Date("UGAMS 43766", 3650, 20)*

*{*

*Outlier("General", 0.05);*

*};*

*};*

*Phase("N212E485 and N179E513")*

*{*

*R_Date("UGAMS 43770", 3540, 20)*

*{*

*Outlier("General", 0.05);*

*};*

*R_Date("UGAMS 43769", 3490, 20)*

*{*

*Outlier("General", 0.05);*

*};*

*R_Date("UGAMS 43776", 3570, 20)*

*{*

*Outlier("General", 0.05);*

*};*

*R_Date("UGAMS 43775", 3650, 20)*

*{*

*Outlier("General", 0.05);*

*};*

*};*

*Sequence("N184E479")*

*{*

*R_Date("UGAMS 43772", 3620, 25)*

*{*

*Outlier("General", 0.05);*

*};*

*R_Date("UGAMS 43771", 3650, 20)*

*{*

*Outlier("General", 0.05);*

*};*

*};*

*};*

*Boundary("End: 38BU7");*

*};*

*Difference("Lapse","Start: 38BU7","End: 38BU7");*

*};*

***38BU7R1M4***

*Plot()*

*{*

*Outlier_Model("General",T(5),U(0,4),"t");*

*Sequence("38BU7 Ring")*

*{*

*Boundary("Start: 38BU7");*

*KDE_Plot("38BU7 Ring Units")*

*{*

*Sequence("N209E512")*

*{*

*R_Date("UGAMS 43768", 3640, 20)*

*{*

*Outlier("General", 0.05);*

*};*

*R_Date("UGAMS 43767", 3660, 20)*

*{*

*Outlier("General", 0.05);*

*};*

*R_Date("UGAMS 43766", 3650, 20)*

*{*

*Outlier("General", 0.05);*

*};*

*};*

*Phase("N212E485 and N179E513")*

*{*

*R_Date("UGAMS 43770", 3540, 20)*

*{*

*Outlier("General", 0.05);*

*};*

*R_Date("UGAMS 43769", 3490, 20)*

*{*

*Outlier("General", 0.05);*

*};*

*R_Date("UGAMS 43776", 3570, 20)*

*{*

*Outlier("General", 0.05);*

*};*

*R_Date("UGAMS 43775", 3650, 20)*

*{*

*Outlier("General", 0.05);*

*};*

*};*

*Sequence("N184E479")*

*{*

*R_Date("UGAMS 43772", 3620, 25)*

*{*

*Outlier("General", 0.05);*

*};*

*R_Date("UGAMS 43771", 3650, 20)*

*{*

*Outlier("General", 0.05);*

*};*

*};*

*Interval(LnN(ln(125),ln(2)));*

*};*

*Boundary("End: 38BU7");*

*};*

*Difference("Lapse","Start: 38BU7","End: 38BU7");*

*};*

***9CH111R1M1***

*Plot()*

*{*

*Sequence(9CH111)*

*{*

*Boundary("Start: 9CH111");*

*Sequence("Unit 1")*

*{*

*R_Date("UGAMS 39525", 3860, 20);*

*R_Date("UGAMS 39524", 3890, 20);*

*R_Date("UGAMS 39523", 3510, 20)*

*{*

*};*

*};*

*Boundary("End: 9CH111");*

*};*

*Difference("Lapse","Start: 9CH111","End: 9CH111");*

*};*

*9CH111R1M2*

*Plot()*

*{*

*Outlier_Model("General",T(5),U(0,4),"t");*

*Sequence(9CH111)*

*{*

*Boundary("Start: 9CH111");*

*Sequence("Unit 1")*

*{*

*R_Date("UGAMS 39525", 3860, 20)*

*{*

*Outlier("General", 0.05);*

*};*

*R_Date("UGAMS 39524", 3890, 20)*

*{*

*Outlier("General", 0.05);*

*};*

*R_Date("UGAMS 39523", 3510, 20)*

*{*

*Outlier("General", 0.05);*

*};*

*};*

*Boundary("End: 9CH111");*

*};*

*Difference("Lapse","Start: 9CH111","End: 9CH111");*

*};*

*9CH111R1M3*

*Plot()*

*{*

*Outlier_Model("Charcoal",Exp(1,-10,0),U(0,3),"t");*

*Sequence(9CH111)*

*{*

*Boundary("Start: 9CH111");*

*Sequence("Unit 1")*

*{*

*R_Date("UGAMS 39525", 3860, 20)*

*{*

*Outlier("Charcoal", 1);*

*};*

*R_Date("UGAMS 39524", 3890, 20)*

*{*

*Outlier("Charcoal", 1);*

*};*

*R_Date("UGAMS 39523", 3510, 20)*

*{*

*};*

*};*

*Boundary("End: 9CH111");*

*};*

*Difference("Lapse","Start: 9CH111","End: 9CH111");*

*};*

*9CH111R1M4*

*Plot()*

*{*

*Outlier_Model("General",T(5),U(0,4),"t");*

*Outlier_Model("Charcoal",Exp(1,-10,0),U(0,3),"t");*

*Sequence(9CH111)*

*{*

*Boundary("Start: 9CH111");*

*Sequence("Unit 1")*

*{*

*R_Date("UGAMS 39525", 3860, 20)*

*{*

*Outlier("Charcoal", 1);*

*};*

*R_Date("UGAMS 39524", 3890, 20)*

*{*

*Outlier("Charcoal", 1);*

*};*

*R_Date("UGAMS 39523", 3510, 20)*

*{*

*Outlier("General", 0.05);*

*};*

*};*

*Boundary("End: 9CH111");*

*};*

*Difference("Lapse","Start: 9CH111","End: 9CH111");*

*};*

*9CH111R1M5*

*Plot()*

*{*

*Sequence(9CH111)*

*{*

*Boundary("Start: 9CH111");*

*KDE_Plot("Unit 1")*

*{*

*R_Date("UGAMS 39525", 3860, 20);*

*R_Date("UGAMS 39524", 3890, 20);*

*R_Date("UGAMS 39523", 3510, 20)*

*{*

*Outlier();*

*};*

*Interval(LnN(ln(125),ln(2)));*

*};*

*Boundary("End: 9CH111");*

*};*

*Difference("Lapse","Start: 9CH111","End: 9CH111");*

*};*

***9CH160R1M1***

*Plot()*

*{*

*Sequence(9CH160)*

*{*

*Boundary("Start: 9CH160");*

*Phase("9CH160 Shell Ring Units")*

*{*

*Sequence("F1")*

*{*

*R_Date("UGAMS 59908", 4410, 25);*

*R_Date("UGAMS 59907", 4430, 25);*

*R_Date("UGAMS 59906", 4300, 25);*

*R_Date("UGAMS 59905", 4290, 25);*

*};*

*Sequence("E1")*

*{*

*R_Date("UGAMS 59922", 4410, 25);*

*R_Date("UGAMS 59921", 4390, 25);*

*R_Date("UGAMS 59920", 4190, 25);*

*};*

*Sequence("C1")*

*{*

*R_Date("UGAMS 59911", 4420, 25);*

*R_Date("UGAMS 59910", 4390, 25);*

*R_Date("UGAMS 59909", 4370, 25);*

*};*

*Sequence("D1")*

*{*

*R_Date("UGAMS 59917", 4390, 25);*

*R_Date("UGAMS 59916", 4330, 25);*

*};*

*};*

*Boundary("End: 9CH160");*

*};*

*Difference("Lapse","Start: 9CH160","End: 9CH160");*

*};*

***9CH160R1M2***

*Plot()*

*{*

*Outlier_Model("General",T(5),U(0,4),"t");*

*Sequence(9CH160)*

*{*

*Boundary("Start: 9CH160");*

*Phase("9CH160 Shell Ring Units")*

*{*

*Sequence("F1")*

*{*

*R_Date("UGAMS 59908", 4410, 25)*

*{*

*Outlier("General", 0.05);*

*};*

*R_Date("UGAMS 59907", 4430, 25)*

*{*

*Outlier("General", 0.05);*

*};*

*R_Date("UGAMS 59906", 4300, 25)*

*{*

*Outlier("General", 0.05);*

*};*

*R_Date("UGAMS 59905", 4290, 25)*

*{*

*Outlier("General", 0.05);*

*};*

*};*

*Sequence("E1")*

*{*

*R_Date("UGAMS 59922", 4410, 25)*

*{*

*Outlier("General", 0.05);*

*};*

*R_Date("UGAMS 59921", 4390, 25)*

*{*

*Outlier("General", 0.05);*

*};*

*R_Date("UGAMS 59920", 4190, 25)*

*{*

*Outlier("General", 0.05);*

*};*

*};*

*Sequence("C1")*

*{*

*R_Date("UGAMS 59911", 4420, 25)*

*{*

*Outlier("General", 0.05);*

*};*

*R_Date("UGAMS 59910", 4390, 25)*

*{*

*Outlier("General", 0.05);*

*};*

*R_Date("UGAMS 59909", 4370, 25)*

*{*

*Outlier("General", 0.05);*

*};*

*};*

*Sequence("D1")*

*{*

*R_Date("UGAMS 59917", 4390, 25)*

*{*

*Outlier("General", 0.05);*

*};*

*R_Date("UGAMS 59916", 4330, 25)*

*{*

*Outlier("General", 0.05);*

*};*

*};*

*};*

*Boundary("End: 9CH160");*

*};*

*Difference("Lapse","Start: 9CH160","End: 9CH160");*

*};*

***9CH160R1M3***

*Plot()*

*{*

*Outlier_Model("General",T(5),U(0,4),"t");*

*Sequence(9CH160)*

*{*

*Boundary("Start: 9CH160");*

*KDE_Plot("9CH160 Shell Ring Units")*

*{*

*Sequence("F1")*

*{*

*R_Date("UGAMS 59908", 4410, 25)*

*{*

*Outlier("General", 0.05);*

*};*

*R_Date("UGAMS 59907", 4430, 25)*

*{*

*Outlier("General", 0.05);*

*};*

*R_Date("UGAMS 59906", 4300, 25)*

*{*

*Outlier("General", 0.05);*

*};*

*R_Date("UGAMS 59905", 4290, 25)*

*{*

*Outlier("General", 0.05);*

*};*

*};*

*Sequence("E1")*

*{*

*R_Date("UGAMS 59922", 4410, 25)*

*{*

*Outlier("General", 0.05);*

*};*

*R_Date("UGAMS 59921", 4390, 25)*

*{*

*Outlier("General", 0.05);*

*};*

*R_Date("UGAMS 59920", 4190, 25)*

*{*

*Outlier("General", 0.05);*

*};*

*};*

*Sequence("C1")*

*{*

*R_Date("UGAMS 59911", 4420, 25)*

*{*

*Outlier("General", 0.05);*

*};*

*R_Date("UGAMS 59910", 4390, 25)*

*{*

*Outlier("General", 0.05);*

*};*

*R_Date("UGAMS 59909", 4370, 25)*

*{*

*Outlier("General", 0.05);*

*};*

*};*

*Sequence("D1")*

*{*

*R_Date("UGAMS 59917", 4390, 25)*

*{*

*Outlier("General", 0.05);*

*};*

*R_Date("UGAMS 59916", 4330, 25)*

*{*

*Outlier("General", 0.05);*

*};*

*};*

*Interval(LnN(ln(125),ln(2)));*

*};*

*Boundary("End: 9CH160");*

*};*

*Difference("Lapse","Start: 9CH160","End: 9CH160");*

*};*

***9CH203R1M1***

*Plot()*

*{*

*Sequence()*

*{*

*Boundary("Start: 9CH203");*

*Sequence("A1")*

*{*

*R_Date("UGAMS 26045", 3830, 30);*

*R_Date("UGAMS 26044", 3720, 30);*

*R_Date("UGAMS 26043", 3660, 30);*

*R_Date("UGAMS 26042", 3730, 30);*

*};*

*Boundary("End: 9CH203");*

*};*

*Difference("Lapse","Start: 9CH203","End: 9CH203");*

*};*

***9CH203R1M2***

*Plot()*

*{*

*Outlier_Model("General",T(5),U(0,4),"t");*

*Sequence()*

*{*

*Boundary("Start: 9CH203");*

*Sequence("A1")*

*{*

*R_Date("UGAMS 26045", 3830, 30)*

*{*

*Outlier("General", 0.05);*

*};*

*R_Date("UGAMS 26044", 3720, 30)*

*{*

*Outlier("General", 0.05);*

*};*

*R_Date("UGAMS 26043", 3660, 30)*

*{*

*Outlier("General", 0.05);*

*};*

*R_Date("UGAMS 26042", 3730, 30)*

*{*

*Outlier("General", 0.05);*

*};*

*};*

*Boundary("End: 9CH203");*

*};*

*Difference("Lapse","Start: 9CH203","End: 9CH203");*

*};*

***9CH203R1M3***

*Plot()*

*{*

*Outlier_Model("General",T(5),U(0,4),"t");*

*Sequence(9CH203)*

*{*

*Boundary("Start: 9CH203");*

*KDE_Plot(9CH203 Units)*

*{*

*Sequence("A1")*

*{*

*R_Date("UGAMS 26045", 3830, 30)*

*{*

*Outlier("General", 0.05);*

*};*

*R_Date("UGAMS 26044", 3720, 30)*

*{*

*Outlier("General", 0.05);*

*};*

*R_Date("UGAMS 26043", 3660, 30)*

*{*

*Outlier("General", 0.05);*

*};*

*R_Date("UGAMS 26042", 3730, 30)*

*{*

*Outlier("General", 0.05);*

*};*

*Interval(LnN(ln(125),ln(2)));*

*};*

*};*

*Boundary("End: 9CH203");*

*};*

*Difference("Lapse","Start: 9CH203","End: 9CH203");*

*};*

***9CH35R1M1***

*Sequence(9CH35)*

*{*

*Boundary("Start: 9CH35");*

*Phase("9CH35")*

*{*

*Sequence("Unit 5")*

*{*

*R_Date("UGAMS-23621", 3800, 25)*

*{*

*};*

*Phase("Sub-phase")*

*{*

*R_Date("UGAMS-23619", 3760, 25)*

*{*

*};*

*R_Date("UGAMS-23620", 3660, 25)*

*{*

*};*

*};*

*};*

*Sequence("Unit 4")*

*{*

*R_Date("UGAMS-23618", 3670, 25)*

*{*

*};*

*R_Date("UGAMS-23617", 3560, 25)*

*{*

*};*

*};*

*Sequence("Unit 6")*

*{*

*R_Date("RC6B", 3650, 25);*

*R_Date("RC6A", 3570, 25);*

*};*

*Sequence("Unit 2")*

*{*

*R_Date("RC2A", 3710, 25)*

*{*

*};*

*R_Date("RC2C", 3720, 25)*

*{*

*};*

*};*

*};*

*Boundary("End: 9CH35");*

*};*

*Difference("Lapse","Start: 9CH35","End: 9CH35");*

*};*

***9CH35R1M2***

*Sequence(9CH35)*

*{*

*Outlier_Model("General",T(5),U(0,4),"t");*

*Boundary("Start: 9CH35");*

*Phase("9CH35")*

*{*

*Sequence("Unit 5")*

*{*

*R_Date("UGAMS-23621", 3800, 25)*

*{*

*Outlier("General", 0.05);*

*};*

*Phase("Sub-phase")*

*{*

*R_Date("UGAMS-23619", 3760, 25)*

*{*

*Outlier("General", 0.05);*

*};*

*R_Date("UGAMS-23620", 3660, 25)*

*{*

*Outlier("General", 0.05);*

*};*

*};*

*};*

*Sequence("Unit 4")*

*{*

*R_Date("UGAMS-23618", 3670, 25)*

*{*

*Outlier("General", 0.05);*

*};*

*R_Date("UGAMS-23617", 3560, 25)*

*{*

*Outlier("General", 0.05);*

*};*

*};*

*Sequence("Unit 6")*

*{*

*R_Date("RC6B", 3650, 25)*

*{*

*Outlier("General", 0.05);*

*};*

*R_Date("RC6A", 3570, 25)*

*{*

*Outlier("General", 0.05);*

*};*

*};*

*Sequence("Unit 2")*

*{*

*R_Date("RC2A", 3710, 25)*

*{*

*Outlier("General", 0.05);*

*};*

*R_Date("RC2C", 3720, 25)*

*{*

*Outlier("General", 0.05);*

*};*

*};*

*};*

*Boundary("End: 9CH35");*

*};*

*Difference("Lapse","Start: 9CH35","End: 9CH35");*

*};*

***9CH35R1M3***

*Sequence(9CH35)*

*{*

*Outlier_Model("Charcoal",Exp(1,-10,0),U(0,3),"t");*

*Boundary("Start: 9CH35");*

*Phase("9CH35")*

*{*

*Sequence("Unit 5")*

*{*

*R_Date("UGAMS-23621", 3800, 25)*

*{*

*Outlier("Charcoal", 1);*

*};*

*Phase("Sub-phase")*

*{*

*R_Date("UGAMS-23619", 3760, 25)*

*{*

*Outlier("Charcoal", 1);*

*};*

*R_Date("UGAMS-23620", 3660, 25)*

*{*

*Outlier("Charcoal", 1);*

*};*

*};*

*};*

*Sequence("Unit 4")*

*{*

*R_Date("UGAMS-23618", 3670, 25)*

*{*

*Outlier("Charcoal", 1);*

*};*

*R_Date("UGAMS-23617", 3560, 25)*

*{*

*Outlier("Charcoal", 1);*

*};*

*};*

*Sequence("Unit 6")*

*{*

*R_Date("RC6B", 3650, 25)*

*{*

*Outlier("Charcoal", 1);*

*};*

*R_Date("RC6A", 3570, 25)*

*{*

*Outlier("Charcoal", 1);*

*};*

*};*

*Sequence("Unit 2")*

*{*

*R_Date("RC2A", 3710, 25)*

*{*

*Outlier("Charcoal", 1);*

*};*

*R_Date("RC2C", 3720, 25)*

*{*

*Outlier("Charcoal", 1);*

*};*

*};*

*};*

*Boundary("End: 9CH35");*

*};*

*Difference("Lapse","Start: 9CH35","End: 9CH35");*

*};*

***9CH35R1M4***

*Sequence(9CH35)*

*{*

*Outlier_Model("General",T(5),U(0,4),"t");*

*Boundary("Start: 9CH35");*

*KDE_Plot("9CH35")*

*{*

*Sequence("Unit 5")*

*{*

*R_Date("UGAMS-23621", 3800, 25)*

*{*

*Outlier("General", 0.05);*

*};*

*Phase("Sub-phase")*

*{*

*R_Date("UGAMS-23619", 3760, 25)*

*{*

*Outlier("General", 0.05);*

*};*

*R_Date("UGAMS-23620", 3660, 25)*

*{*

*Outlier("General", 0.05);*

*};*

*};*

*};*

*Sequence("Unit 4")*

*{*

*R_Date("UGAMS-23618", 3670, 25)*

*{*

*Outlier("General", 0.05);*

*};*

*R_Date("UGAMS-23617", 3560, 25)*

*{*

*Outlier("General", 0.05);*

*};*

*};*

*Sequence("Unit 6")*

*{*

*R_Date("RC6B", 3650, 25)*

*{*

*Outlier("General", 0.05);*

*};*

*R_Date("RC6A", 3570, 25)*

*{*

*Outlier("General", 0.05);*

*};*

*};*

*Sequence("Unit 2")*

*{*

*R_Date("RC2A", 3710, 25)*

*{*

*Outlier("General", 0.05);*

*};*

*R_Date("RC2C", 3720, 25)*

*{*

*Outlier("General", 0.05);*

*};*

*};*

*Interval(LnN(ln(200),ln(2)));*

*};*

*Boundary("End: 9CH35");*

*};*

*Difference("Lapse","Start: 9CH35","End: 9CH35");*

*};*

***9CH4R1M1***

*Plot()*

*{*

*Sequence(Bilbo)*

*{*

*Boundary("Start: 9CH4");*

*Phase("9CH4 Units")*

*{*

*Phase ("Haag's Unit Zone 4, 7-8; Waring Units")*

*{*

*R_Date("M-1109", 3700, 129);*

*R_Date("M-1111", 3820, 129);*

*R_Date("O-1047", 4125, 119);*

*R_Date("O-1046", 5550, 119)*

*{*

*Outlier();*

*};*

*};*

*Sequence("Crook Unit 14")*

*{*

*R_Date("UGA-10678", 3720, 180);*

*R_Date("UGA 10677", 3730, 280);*

*R_Date("UGAMS 10676", 3630, 100);*

*};*

*};*

*Boundary("End: 9CH4");*

*};*

*Difference("Lapse","Start: 9CH4","End: 9CH4");*

*};*

***9CH4R1M2***

*Plot()*

*{*

*Outlier_Model("General",T(5),U(0,4),"t");*

*Sequence(9CH61)*

*{*

*Boundary("Start: 9CH4");*

*Sequence("P1")*

*{*

*R_Date("UGAMS 39514", 3870, 20)*

*{*

*Outlier("General", 0.05);*

*};*

*R_Date("UGAMS 39513", 3880, 20)*

*{*

*Outlier("General", 0.05);*

*};*

*R_Date("UGAMS 39512", 3870, 20)*

*{*

*Outlier("General", 0.05);*

*};*

*R_Date("UGAMS 39511", 3790, 20)*

*{*

*Outlier("General", 0.05);*

*};*

*R_Date("UGAMS 39510", 3400, 20)*

*{*

*Outlier("General", 0.05);*

*};*

*};*

*Boundary("End: 9CH4");*

*};*

*Difference("Lapse","Start: 9CH4","End: 9CH4");*

*};*

***9CH4R1M3***

*Plot()*

*{*

*Outlier_Model("Charcoal",Exp(1,-10,0),U(0,3),"t");*

*Sequence(Bilbo)*

*{*

*Boundary("Start: 9CH4");*

*Phase("9CH4 Units")*

*{*

*Phase ("Haag's Unit Zone 4, 7-8; Waring Units")*

*{*

*R_Date("M-1109", 3700, 129)*

*{*

*Outlier("Charcoal", 1);*

*};*

*R_Date("M-1111", 3820, 129)*

*{*

*Outlier("Charcoal", 1);*

*};*

*R_Date("O-1047", 4125, 119)*

*{*

*Outlier("Charcoal", 1);*

*};*

*R_Date("O-1046", 5550, 119)*

*{*

*Outlier();*

*};*

*};*

*Sequence("Crook Unit 14")*

*{*

*R_Date("UGA-10678", 3720, 180)*

*{*

*Outlier("Charcoal", 1);*

*};*

*R_Date("UGA 10677", 3730, 280)*

*{*

*Outlier("Charcoal", 1);*

*};*

*R_Date("UGAMS 10676", 3630, 100)*

*{*

*Outlier("Charcoal", 1);*

*};*

*};*

*};*

*Boundary("End: 9CH4");*

*};*

*Difference("Lapse","Start: 9CH4","End: 9CH4");*

*};*

***9CH4R1M4***

*Plot()*

*{*

*Outlier_Model("General",T(5),U(0,4),"t");*

*Sequence(Bilbo)*

*{*

*Boundary("Start: 9CH4");*

*KDE_Plot("9CH4 Units")*

*{*

*Phase ("Haag's Unit Zone 4, 7-8; Waring Units")*

*{*

*R_Date("M-1109", 3700, 129)*

*{*

*Outlier("General", 0.05);*

*};*

*R_Date("M-1111", 3820, 129)*

*{*

*Outlier("General", 0.05);*

*};*

*R_Date("O-1047", 4125, 119)*

*{*

*Outlier();*

*};*

*R_Date("O-1046", 5550, 119)*

*{*

*Outlier();*

*};*

*};*

*Sequence("Crook Unit 14")*

*{*

*R_Date("UGA-10678", 3720, 180)*

*{*

*Outlier("General", 0.05);*

*};*

*R_Date("UGA 10677", 3730, 280)*

*{*

*Outlier("General", 0.05);*

*};*

*R_Date("UGAMS 10676", 3630, 100)*

*{*

*Outlier("General", 0.05);*

*};*

*};*

*Interval(LnN(ln(125),ln(2)));*

*};*

*Boundary("End: 9CH4");*

*};*

*Difference("Lapse","Start: 9CH4","End: 9CH4");*

*};*

***9CH61R1M1***

*Plot()*

*{*

*Sequence(9CH61)*

*{*

*Boundary("Start: 9CH61");*

*Sequence("P1")*

*{*

*R_Date("UGAMS 39514", 3870, 20);*

*R_Date("UGAMS 39513", 3880, 20);*

*R_Date("UGAMS 39512", 3870, 20);*

*R_Date("UGAMS 39511", 3790, 20);*

*R_Date("UGAMS 39510", 3400, 20);*

*};*

*Boundary("End: 9CH61");*

*};*

*Difference("Lapse","Start: 9CH61","End: 9CH61");*

*};*

***9CH61R1M2***

*Plot()*

*{*

*Outlier_Model("General",T(5),U(0,4),"t");*

*Sequence(9CH61)*

*{*

*Boundary("Start: 9CH61");*

*Sequence("P1")*

*{*

*R_Date("UGAMS 39514", 3870, 20)*

*{*

*Outlier("General", 0.05);*

*};*

*R_Date("UGAMS 39513", 3880, 20)*

*{*

*Outlier("General", 0.05);*

*};*

*R_Date("UGAMS 39512", 3870, 20)*

*{*

*Outlier("General", 0.05);*

*};*

*R_Date("UGAMS 39511", 3790, 20)*

*{*

*Outlier("General", 0.05);*

*};*

*R_Date("UGAMS 39510", 3400, 20)*

*{*

*Outlier("General", 0.05);*

*};*

*};*

*Boundary("End: 9CH61");*

*};*

*Difference("Lapse","Start: 9CH61","End: 9CH61");*

*};*

***9CH61R1M3***

*Plot()*

*{*

*Outlier_Model("Charcoal",Exp(1,-10,0),U(0,3),"t");*

*Sequence(9CH61)*

*{*

*Boundary("Start: 9CH61");*

*Sequence("P1")*

*{*

*R_Date("UGAMS 39514", 3870, 20)*

*{*

*};*

*R_Date("UGAMS 39513", 3880, 20)*

*{*

*Outlier("Charcoal", 1);*

*};*

*R_Date("UGAMS 39512", 3870, 20)*

*{*

*};*

*R_Date("UGAMS 39511", 3790, 20)*

*{*

*Outlier("Charcoal", 1);*

*};*

*R_Date("UGAMS 39510", 3400, 20)*

*{*

*};*

*};*

*Boundary("End: 9CH61");*

*};*

*Difference("Lapse","Start: 9CH61","End: 9CH61");*

*};*

***9CH61R1M4***

*Plot()*

*{*

*Outlier_Model("Charcoal",Exp(1,-10,0),U(0,3),"t");*

*Outlier_Model("General",T(5),U(0,4),"t");*

*Sequence(9CH61)*

*{*

*Boundary("Start: 9CH61");*

*Sequence("P1")*

*{*

*R_Date("UGAMS 39514", 3870, 20)*

*{*

*Outlier("General", 0.05);*

*};*

*R_Date("UGAMS 39513", 3880, 20)*

*{*

*Outlier("Charcoal", 1);*

*};*

*R_Date("UGAMS 39512", 3870, 20)*

*{*

*Outlier("General", 0.05);*

*};*

*R_Date("UGAMS 39511", 3790, 20)*

*{*

*Outlier("Charcoal", 1);*

*};*

*R_Date("UGAMS 39510", 3400, 20)*

*{*

*Outlier("General", 0.05);*

*};*

*};*

*Boundary("End: 9CH61");*

*};*

*Difference("Lapse","Start: 9CH61","End: 9CH61");*

*};*

***9CH61R1M5***

*Plot()*

*{*

*Outlier_Model("General",T(5),U(0,4),"t");*

*Sequence(9CH61)*

*{*

*Boundary("Start: 9CH61");*

*KDE_Plot()*

*{*

*Sequence("P1")*

*{*

*R_Date("UGAMS 39514", 3870, 20)*

*{*

*Outlier("General", 0.05);*

*};*

*R_Date("UGAMS 39513", 3880, 20)*

*{*

*Outlier("General", 0.05);*

*};*

*R_Date("UGAMS 39512", 3870, 20)*

*{*

*Outlier("General", 0.05);*

*};*

*R_Date("UGAMS 39511", 3790, 20)*

*{*

*Outlier("General", 0.05);*

*};*

*R_Date("UGAMS 39510", 3400, 20)*

*{*

*Outlier();*

*};*

*Interval(LnN(ln(125),ln(2)));*

*};*

*};*

*Boundary("End: 9CH61");*

*};*

*Difference("Lapse","Start: 9CH61","End: 9CH61");*

*};*

***9MC23R1M1***

*Plot()*

*{*

*Sequence(9MC23 Ring I)*

*{*

*Boundary("Start: 9MC23 Ring I");*

*Phase()*

*{*

*Sequence("Waring Unit 4")*

*{*

*R_Date("UGAMS 52191", 3790, 20);*

*R_Date("UGAMS 52190", 3810, 20);*

*Phase("1")*

*{*

*R_Date("UGAMS 52189", 3820, 20);*

*R_Date("52188", 3810, 20);*

*};*

*R_Date("UGAMS 52187", 3810, 20);*

*R_Date("UGAMS 52186", 3840, 20);*

*Phase("2")*

*{*

*R_Date("UGAMS 52185", 3830, 20);*

*R_Date("UGAMS 52184", 3670, 20)*

*{*

*Outlier();*

*};*

*};*

*R_Date("UGAMS 52183", 3780, 20);*

*R_Date("UGAMS 52182", 3750, 20);*

*};*

*Phase(Thompson Unit 1-2003)*

*{*

*R_Date("UGAMS 15085", 3730, 60);*

*R_Date("UGAMS 15084", 3610, 60);*

*};*

*};*

*Boundary("End: 9MC23 Ring I");*

*};*

*Difference("Lapse","Start: 9MC23 Ring I ","End: 9MC23 Ring I ");*

*};*

***9MC23R1M2***

*Plot()*

*{*

*Sequence(9MC23 Ring I)*

*{*

*Boundary("Start: 9MC23 Ring I");*

*Phase()*

*{*

*Sequence("Waring Unit 4")*

*{*

*R_Date("UGAMS 52191", 3790, 20);*

*R_Date("UGAMS 52190", 3810, 20);*

*Phase("1")*

*{*

*R_Date("UGAMS 52189", 3820, 20);*

*R_Date("52188", 3810, 20);*

*};*

*R_Date("UGAMS 52187", 3810, 20);*

*R_Date("UGAMS 52186", 3840, 20);*

*Phase("2")*

*{*

*R_Date("UGAMS 52185", 3830, 20);*

*R_Date("UGAMS 52184", 3670, 20)*

*{*

*Outlier();*

*};*

*};*

*R_Date("UGAMS 52183", 3780, 20);*

*R_Date("UGAMS 52182", 3750, 20);*

*};*

*Phase(Thompson Unit 1-2003)*

*{*

*R_Date("UGAMS 15085", 3730, 60);*

*R_Date("UGAMS 15084", 3610, 60)*

*{*

*Outlier();*

*};*

*};*

*};*

*Boundary("End: 9MC23 Ring I");*

*};*

*Difference("Lapse","Start: 9MC23 Ring I","End: 9MC23 Ring I");*

*};*

***9MC23R1M3***

*Plot()*

*{*

*Outlier_Model("Charcoal",Exp(1,-10,0),U(0,3),"t");*

*Outlier_Model("General",T(5),U(0,4),"t");*

*Sequence(9MC23 Ring I)*

*{*

*Boundary("Start: 9MC23 Ring I");*

*Phase()*

*{*

*Sequence("Waring Unit 4")*

*{*

*R_Date("UGAMS 52191", 3790, 20)*

*{*

*Outlier("General", 0.05);*

*};*

*R_Date("UGAMS 52190", 3810, 20)*

*{*

*Outlier("General", 0.05);*

*};*

*Phase("1")*

*{*

*R_Date("UGAMS 52189", 3820, 20)*

*{*

*Outlier("General", 0.05);*

*};*

*R_Date("52188", 3810, 20)*

*{*

*Outlier("General", 0.05);*

*};*

*};*

*R_Date("UGAMS 52187", 3810, 20)*

*{*

*Outlier("General", 0.05);*

*};*

*R_Date("UGAMS 52186", 3840, 20)*

*{*

*Outlier("General", 0.05);*

*};*

*Phase("2")*

*{*

*R_Date("UGAMS 52185", 3830, 20)*

*{*

*Outlier("General", 0.05);*

*};*

*R_Date("UGAMS 52184", 3670, 20)*

*{*

*Outlier();*

*};*

*};*

*R_Date("UGAMS 52183", 3780, 20)*

*{*

*Outlier("General", 0.05);*

*};*

*R_Date("UGAMS 52182", 3750, 20)*

*{*

*Outlier("General", 0.05);*

*};*

*};*

*Phase(Thompson Unit 1-2003)*

*{*

*R_Date("UGAMS 15085", 3730, 60)*

*{*

*Outlier("Charcoal", 1);*

*};*

*R_Date("UGAMS 15084", 3610, 60)*

*{*

*Outlier();*

*};*

*};*

*};*

*Boundary("End: 9MC23 Ring I");*

*};*

*Difference("Lapse","Start: 9MC23 Ring I","End: 9MC23 Ring I");*

*};*

*9MC23R1M4*

*Plot()*

*{*

*Outlier_Model("Charcoal",Exp(1,-10,0),U(0,3),"t");*

*Outlier_Model("General",T(5),U(0,4),"t");*

*Sequence(9MC23 Ring I)*

*{*

*Boundary("Start: 9MC23 Ring I");*

*Phase()*

*{*

*Sequence("Waring Unit 4")*

*{*

*R_Date("UGAMS 52191", 3790, 20)*

*{*

*Outlier("General", 0.05);*

*};*

*R_Date("UGAMS 52190", 3810, 20)*

*{*

*Outlier("General", 0.05);*

*};*

*Phase("1")*

*{*

*R_Date("UGAMS 52189", 3820, 20)*

*{*

*Outlier("General", 0.05);*

*};*

*R_Date("52188", 3810, 20)*

*{*

*Outlier("General", 0.05);*

*};*

*};*

*R_Date("UGAMS 52187", 3810, 20)*

*{*

*Outlier("General", 0.05);*

*};*

*R_Date("UGAMS 52186", 3840, 20)*

*{*

*Outlier("General", 0.05);*

*};*

*Phase("2")*

*{*

*R_Date("UGAMS 52185", 3830, 20)*

*{*

*Outlier("General", 0.05);*

*};*

*R_Date("UGAMS 52184", 3670, 20)*

*{*

*Outlier();*

*};*

*};*

*R_Date("UGAMS 52183", 3780, 20)*

*{*

*Outlier("General", 0.05);*

*};*

*R_Date("UGAMS 52182", 3750, 20)*

*{*

*Outlier("General", 0.05);*

*};*

*};*

*Phase(Thompson Unit 1-2003)*

*{*

*R_Date("UGAMS 15085", 3730, 60)*

*{*

*Outlier("Charcoal", 1);*

*};*

*R_Date("UGAMS 15084", 3610, 60)*

*{*

*Outlier();*

*};*

*};*

*};*

*Boundary("End: 9MC23 Ring I");*

*};*

*Difference("Lapse","Start: 9MC23 Ring I ","End: 9MC23 Ring I ");*

*};*

***9MC23R1M5***

*Plot()*

*{*

*Outlier_Model("Charcoal",Exp(1,-10,0),U(0,3),"t");*

*Outlier_Model("General",T(5),U(0,4),"t");*

*Sequence(9MC23 Ring I)*

*{*

*Boundary("Start: 9MC23 Ring I");*

*KDE_Plot(Sapelo_RI_Units)*

*{*

*Sequence("Waring Unit 4")*

*{*

*R_Date("UGAMS 52191", 3790, 20)*

*{*

*Outlier("General", 0.05);*

*};*

*R_Date("UGAMS 52190", 3810, 20)*

*{*

*Outlier("General", 0.05);*

*};*

*Phase("1")*

*{*

*R_Date("UGAMS 52189", 3820, 20)*

*{*

*Outlier("General", 0.05);*

*};*

*R_Date("52188", 3810, 20)*

*{*

*Outlier("General", 0.05);*

*};*

*};*

*R_Date("UGAMS 52187", 3810, 20)*

*{*

*Outlier("General", 0.05);*

*};*

*R_Date("UGAMS 52186", 3840, 20)*

*{*

*Outlier("General", 0.05);*

*};*

*Phase("2")*

*{*

*R_Date("UGAMS 52185", 3830, 20)*

*{*

*Outlier("General", 0.05);*

*};*

*R_Date("UGAMS 52184", 3670, 20)*

*{*

*Outlier();*

*};*

*};*

*R_Date("UGAMS 52183", 3780, 20)*

*{*

*Outlier("General", 0.05);*

*};*

*R_Date("UGAMS 52182", 3750, 20)*

*{*

*Outlier("General", 0.05);*

*};*

*};*

*Phase(Thompson Unit 1-2003)*

*{*

*R_Date("UGAMS 15085", 3730, 60)*

*{*

*Outlier("Charcoal", 1);*

*};*

*R_Date("UGAMS 15084", 3610, 60)*

*{*

*Outlier();*

*};*

*};*

*Interval(LnN(ln(125),ln(2)));*

*};*

*Boundary("End: 9MC23 Ring I");*

*};*

*Difference("Lapse","Start: 9MC23 Ring I","End: 9MC23 Ring I");*

*};*

***9MC23R2M1***

*Plot()*

*{*

*Sequence(9MC23 Ring II)*

*{*

*Boundary("Start: 9MC23 Ring II");*

*Phase(9MC23 Ring II)*

*{*

*Sequence("Unit 1-2006")*

*{*

*R_Date("UGAMS 42752", 3810, 20)*

*{*

*};*

*R_Date("UGAMS 42751", 3770, 20)*

*{*

*};*

*Phase("Level 5 Samples")*

*{*

*R_Date("UGAMS 1425", 3890, 60)*

*{*

*Outlier();*

*};*

*R_Date("UGAMS 1424", 3750, 70)*

*{*

*};*

*};*

*R_Date("UGAMS 42750", 3800, 20)*

*{*

*};*

*};*

*Phase(A-1)*

*{*

*R_Date("UGAMS 52175", 3680, 20)*

*{*

*};*

*};*

*};*

*Boundary("End: 9MC23 Ring II");*

*};*

*Difference("Lapse","Start: 9MC23 Ring II ","End: 9MC23 Ring II ");*

*};*

***9MC23R2M2***

*Plot()*

*{*

*Outlier_Model("General",T(5),U(0,4),"t");*

*Sequence(9MC23 Ring II)*

*{*

*Boundary("Start: 9MC23 Ring II");*

*Phase(9MC23 Ring II)*

*{*

*Sequence("Unit 1-2006")*

*{*

*R_Date("UGAMS 42752", 3810, 20)*

*{*

*Outlier("General", 0.05);*

*};*

*R_Date("UGAMS 42751", 3770, 20)*

*{*

*Outlier("General", 0.05);*

*};*

*Phase("Level 5 Samples")*

*{*

*R_Date("UGAMS 1425", 3890, 60)*

*{*

*Outlier();*

*};*

*R_Date("UGAMS 1424", 3750, 70)*

*{*

*Outlier("General", 0.05);*

*};*

*};*

*R_Date("UGAMS 42750", 3800, 20)*

*{*

*Outlier("General", 0.05);*

*};*

*};*

*Phase(A-1)*

*{*

*R_Date("UGAMS 52175", 3680, 20)*

*{*

*Outlier("General", 0.05);*

*};*

*};*

*};*

*Boundary("End: 9MC23 Ring II");*

*};*

*Difference("Lapse","Start: 9MC23 Ring II","End: 9MC23 Ring II");*

*};*

***9MC23R2M3***

*Plot()*

*{*

*Outlier_Model("Charcoal",Exp(1,-10,0),U(0,3),"t");*

*Sequence(9MC23 Ring II)*

*{*

*Boundary("Start: 9MC23 Ring II");*

*Phase(9MC23 Ring II)*

*{*

*Sequence("Unit 1-2006")*

*{*

*R_Date("UGAMS 42752", 3810, 20)*

*{*

*Outlier("Charcoal", 1);*

*};*

*R_Date("UGAMS 42751", 3770, 20)*

*{*

*Outlier("Charcoal", 1);*

*};*

*Phase("Level 5 Samples")*

*{*

*R_Date("UGAMS 1425", 3890, 60)*

*{*

*Outlier();*

*};*

*R_Date("UGAMS 1424", 3750, 70)*

*{*

*Outlier("Charcoal", 1);*

*};*

*};*

*R_Date("UGAMS 42750", 3800, 20)*

*{*

*Outlier("Charcoal", 1);*

*};*

*};*

*Phase(A-1)*

*{*

*R_Date("UGAMS 52175", 3680, 20)*

*{*

*};*

*};*

*Boundary("End: 9MC23 Ring II");*

*};*

*Difference("Lapse","Start: 9MC23 Ring II","End: 9MC23 Ring II");*

*};*

***9MC23R2M4***

*Plot()*

*{*

*Outlier_Model("Charcoal",Exp(1,-10,0),U(0,3),"t");*

*Outlier_Model("General",T(5),U(0,4),"t");*

*Sequence(9MC23 Ring II)*

*{*

*Boundary("Start: 9MC23 Ring II");*

*KDE_Plot(9MC23 Ring II)*

*{*

*Sequence("Unit 1-2006")*

*{*

*R_Date("UGAMS 42752", 3810, 20)*

*{*

*Outlier("Charcoal", 1);*

*};*

*R_Date("UGAMS 42751", 3770, 20)*

*{*

*Outlier("Charcoal", 1);*

*};*

*Phase("Level 5 Samples")*

*{*

*R_Date("UGAMS 1425", 3890, 60)*

*{*

*Outlier();*

*};*

*R_Date("UGAMS 1424", 3750, 70)*

*{*

*Outlier("Charcoal", 1);*

*};*

*};*

*R_Date("UGAMS 42750", 3800, 20)*

*{*

*Outlier("Charcoal", 1);*

*};*

*};*

*Phase(A-1)*

*{*

*R_Date("UGAMS 52175", 3680, 20)*

*{*

*Outlier("General",0.05);*

*};*

*};*

*Interval(LnN(ln(125),ln(2)));*

*};*

*Boundary("End: 9MC23 Ring II");*

*};*

*Difference("Lapse","Start: 9MC23 Ring II ","End: 9MC23 Ring II ");*

*};*

***9MC23R3M1***

*Plot()*

*{*

*Sequence(9MC23 Ring III)*

*{*

*Boundary("Start: 9MC23 Ring III");*

*Phase(Ring III)*

*{*

*Sequence("1")*

*{*

*R_Date("52181", 3570, 20)*

*{*

*Outlier();*

*};*

*R_Date("52180", 3690, 20);*

*R_Date("52179", 3660, 20);*

*R_Date("15083", 3730, 60);*

*R_Date("52174", 3770, 20)*

*{*

*Outlier();*

*};*

*R_Date("15082", 3560, 50)*

*{*

*Outlier();*

*};*

*R_Date("52178", 3620, 20);*

*R_Date("52177", 3590, 20);*

*};*

*Phase()*

*{*

*R_Date("15086", 3730, 50);*

*};*

*};*

*Boundary("End: 9MC23 Ring III");*

*};*

*Difference("Lapse","Start: 9MC23 Ring III","End: 9MC23 Ring III");*

*};*

***9MC23R3M2***

*Plot()*

*{*

*Sequence(9MC23 Ring III)*

*{*

*Boundary("Start: 9MC23 Ring III");*

*Phase(Ring III)*

*{*

*Phase("1")*

*{*

*R_Date("52181", 3570, 20)*

*{*

*};*

*R_Date("52180", 3690, 20);*

*R_Date("52179", 3660, 20);*

*R_Date("15083", 3730, 60);*

*R_Date("52174", 3770, 20)*

*{*

*};*

*R_Date("15082", 3560, 50)*

*{*

*};*

*R_Date("52178", 3620, 20);*

*R_Date("52177", 3590, 20);*

*};*

*Phase()*

*{*

*R_Date("15086", 3730, 50);*

*};*

*Interval("Duration of Sapelo Ring III");*

*};*

*Boundary("End: 9MC23 Ring III");*

*};*

*Difference("Lapse","Start: 9MC23 Ring III","End: 9MC23 Ring III");*

*};*

***9MC23R3M3***

*Plot()*

*{*

*Sequence(9MC23 Ring III)*

*{*

*Boundary("Start: 9MC23 Ring III");*

*Phase(Ring III)*

*{*

*Sequence("1")*

*{*

*R_Date("UGAMS 52181", 3570, 20)*

*{*

*Outlier();*

*};*

*R_Date("UGAMS 52180", 3690, 20)*

*{*

*};*

*R_Date("UGAMS 52179", 3660, 20)*

*{*

*};*

*R_Date("UGAMS 15083", 3730, 60)*

*{*

*};*

*Phase("Lvl 2-5 with Feat. 4")*

*{*

*R_Date("UGAMS 52174", 3770, 20)*

*{*

*Outlier();*

*};*

*R_Date("UGAMS 15082", 3560, 50)*

*{*

*};*

*R_Date("UGAMS 52178", 3620, 20)*

*{*

*};*

*R_Date("UGAMS 52177", 3590, 20)*

*{*

*};*

*};*

*};*

*Phase()*

*{*

*R_Date("UGAMS 15086", 3730, 50)*

*{*

*};*

*};*

*};*

*Boundary("End: 9MC23 Ring III");*

*};*

*Difference("Lapse","Start: 9MC23 Ring III","End: 9MC23 Ring III");*

*};*

***9MC23R3M4***

*Plot()*

*{*

*Outlier_Model("General",T(5),U(0,4),"t");*

*Sequence(9MC23 Ring III)*

*{*

*Boundary("Start: 9MC23 Ring III");*

*Phase(Ring III)*

*{*

*Sequence("1")*

*{*

*R_Date("52181", 3570, 20)*

*{*

*Outlier();*

*};*

*R_Date("52180", 3690, 20)*

*{*

*Outlier("General", 0.05);*

*};*

*R_Date("52179", 3660, 20)*

*{*

*Outlier("General", 0.05);*

*};*

*R_Date("15083", 3730, 60)*

*{*

*Outlier("General", 0.05);*

*};*

*};*

*Phase()*

*{*

*R_Date("15086", 3730, 50)*

*{*

*Outlier("General", 0.05);*

*};*

*};*

*Interval("Duration of Sapelo Ring III");*

*Phase("Lvl 2-5 with Feat. 4")*

*{*

*R_Date("52174", 3770, 20)*

*{*

*Outlier();*

*};*

*R_Date("15082", 3560, 50)*

*{*

*Outlier("General", 0.05);*

*};*

*R_Date("52178", 3620, 20)*

*{*

*Outlier("General", 0.05);*

*};*

*R_Date("52177", 3590, 20)*

*{*

*Outlier("General", 0.05);*

*};*

*};*

*};*

*Boundary("End: 9MC23 Ring III");*

*};*

*Difference("Lapse","Start: 9MC23 Ring III","End: 9MC23 Ring III");*

*};*

***9MC23R3M5***

*Plot()*

*{*

*Outlier_Model("Charcoal",Exp(1,-10,0),U(0,3),"t");*

*Outlier_Model("General",T(5),U(0,4),"t");*

*Sequence(9MC23 Ring III)*

*{*

*Boundary("Start: 9MC23 Ring III");*

*Phase(Ring III)*

*{*

*Sequence("1")*

*{*

*R_Date("52181", 3570, 20)*

*{*

*Outlier();*

*};*

*R_Date("52180", 3690, 20)*

*{*

*Outlier("General", 0.05);*

*};*

*R_Date("52179", 3660, 20)*

*{*

*Outlier("Charcoal", 1);*

*};*

*R_Date("15083", 3730, 60)*

*{*

*Outlier("Charcoal", 1);*

*};*

*};*

*Phase("Lvl 2-5 with Feat. 4")*

*{*

*R_Date("52174", 3770, 20)*

*{*

*Outlier();*

*};*

*R_Date("15082", 3560, 50)*

*{*

*Outlier("Charcoal", 1);*

*};*

*R_Date("52178", 3620, 20)*

*{*

*Outlier("General", 0.05);*

*};*

*R_Date("52177", 3590, 20)*

*{*

*Outlier("Charcoal", 1);*

*};*

*};*

*Phase()*

*{*

*R_Date("15086", 3730, 50)*

*{*

*Outlier("Charcoal", 1);*

*};*

*};*

*Interval("Duration of Sapelo Ring III");*

*};*

*Boundary("End: 9MC23 Ring III");*

*};*

*Difference("Lapse","Start: 9MC23 Ring III","End: 9MC23 Ring III");*

*};*

***9MC23R3M6***

*Plot()*

*{*

*Outlier_Model("Charcoal",Exp(1,-10,0),U(0,3),"t");*

*Outlier_Model("General",T(5),U(0,4),"t");*

*Sequence(9MC23 Ring III)*

*{*

*Boundary("Start: 9MC23 Ring III");*

*KDE_Plot(9MC23 Ring III)*

*{*

*Sequence("1")*

*{*

*R_Date("52181", 3570, 20)*

*{*

*Outlier();*

*};*

*R_Date("52180", 3690, 20)*

*{*

*Outlier("General", 0.05);*

*};*

*R_Date("52179", 3660, 20)*

*{*

*Outlier("Charcoal", 1);*

*};*

*R_Date("15083", 3730, 60)*

*{*

*Outlier("Charcoal", 1);*

*};*

*};*

*Phase("Lvl 2-5 with Feat. 4")*

*{*

*R_Date("52174", 3770, 20)*

*{*

*Outlier();*

*};*

*R_Date("15082", 3560, 50)*

*{*

*Outlier("Charcoal", 1);*

*};*

*R_Date("52178", 3620, 20)*

*{*

*Outlier("General", 0.05);*

*};*

*R_Date("52177", 3590, 20)*

*{*

*Outlier("Charcoal", 1);*

*};*

*};*

*Phase()*

*{*

*R_Date("15086", 3730, 50)*

*{*

*Outlier("Charcoal", 1);*

*};*

*};*

*Interval(LnN(ln(125),ln(2)));*

*};*

*Boundary("End: 9MC23 Ring III");*

*};*

*Difference("Lapse","Start: 9MC23 Ring III","End: 9MC23 Ring III");*

*};*

***9MC87R1M1***

*Plot()*

*{*

*Sequence(Bush Krick)*

*{*

*Boundary("Start: 9MC87");*

*Sequence("Pit 1")*

*{*

*R_Date("UGAMS 39522", 3900, 20);*

*R_Date("UGAMS 39521", 3800, 20);*

*R_Date("UGAMS 39520", 3750, 20);*

*R_Date("UGAMS 39518", 3770, 20);*

*R_Date("UGAMS_39517", 3810, 20)*

*{*

*Outlier();*

*};*

*R_Date("UGAMS 39516", 3710, 20);*

*R_Date("UGAMS 39515", 3650, 20);*

*};*

*Boundary("End: 9MC87");*

*};*

*Difference("Lapse","Start: 9MC87","End: 9MC87");*

*};*

***9MC87R1M2***

*Plot()*

*{*

*Outlier_Model("General",T(5),U(0,4),"t");*

*Sequence(Bush Krick)*

*{*

*Boundary("Start: 9MC87");*

*Sequence("Pit 1")*

*{*

*R_Date("UGAMS 39522", 3900, 20)*

*{*

*Outlier("General", 0.05);*

*};*

*R_Date("UGAMS 39521", 3800, 20)*

*{*

*Outlier("General", 0.05);*

*};*

*R_Date("UGAMS 39520", 3750, 20)*

*{*

*Outlier("General", 0.05);*

*};*

*R_Date("UGAMS 39518", 3770, 20)*

*{*

*Outlier("General", 0.05);*

*};*

*R_Date("UGAMS_39517", 3810, 20)*

*{*

*Outlier();*

*};*

*R_Date("UGAMS 39516", 3710, 20)*

*{*

*Outlier("General", 0.05);*

*};*

*R_Date("UGAMS 39515", 3650, 20)*

*{*

*Outlier("General", 0.05);*

*};*

*};*

*Boundary("End: 9MC87");*

*};*

*Difference("Lapse","Start: 9MC87","End: 9MC87");*

*};*

***9MC87R1M3***

*Plot()*

*{*

*Outlier_Model("Charcoal",Exp(1,-10,0),U(0,3),"t");*

*Sequence(Bush Krick)*

*{*

*Boundary("Start: 9MC87");*

*Sequence("Pit 1")*

*{*

*R_Date("UGAMS 39522", 3900, 20)*

*{*

*Outlier("Charcoal", 1);*

*};*

*R_Date("UGAMS 39521", 3800, 20)*

*{*

*};*

*R_Date("UGAMS 39520", 3750, 20)*

*{*

*Outlier("Charcoal", 1);*

*};*

*R_Date("UGAMS 39518", 3770, 20)*

*{*

*};*

*R_Date("UGAMS_39517", 3810, 20)*

*{*

*Outlier();*

*};*

*R_Date("UGAMS 39516", 3710, 20)*

*{*

*Outlier("Charcoal", 1);*

*};*

*R_Date("UGAMS 39515", 3650, 20)*

*{*

*};*

*};*

*Boundary("End: 9MC87");*

*};*

*Difference("Lapse","Start: 9MC87","End: 9MC87");*

*};*

***9MC87R1M4***

*Plot()*

*{*

*Outlier_Model("Charcoal",Exp(1,-10,0),U(0,3),"t");*

*Outlier_Model("General",T(5),U(0,4),"t");*

*Sequence(Bush Krick)*

*{*

*Boundary("Start: 9MC87");*

*Sequence("Pit 1")*

*{*

*R_Date("UGAMS 39522", 3900, 20)*

*{*

*Outlier("Charcoal", 1);*

*};*

*R_Date("UGAMS 39521", 3800, 20)*

*{*

*Outlier("General", 0.05);*

*};*

*R_Date("UGAMS 39520", 3750, 20)*

*{*

*Outlier("Charcoal", 1);*

*};*

*R_Date("UGAMS 39518", 3770, 20)*

*{*

*Outlier("General", 0.05);*

*};*

*R_Date("UGAMS_39517", 3810, 20)*

*{*

*Outlier();*

*};*

*R_Date("UGAMS 39516", 3710, 20)*

*{*

*Outlier("Charcoal", 1);*

*};*

*R_Date("UGAMS 39515", 3650, 20)*

*{*

*Outlier("General", 0.05);*

*};*

*};*

*Boundary("End: 9MC87");*

*};*

*Difference("Lapse","Start: 9MC87","End: 9MC87");*

*};*

***9MC87R1M5***

*Plot()*

*{*

*Outlier_Model("General",T(5),U(0,4),"t");*

*Sequence(9MC87)*

*{*

*Boundary("Start: 9MC87");*

*KDE_Plot(9MC87 Units)*

*{*

*Sequence("Pit 1")*

*{*

*R_Date("UGAMS 39522", 3900, 20)*

*{*

*Outlier("General", 0.05);*

*};*

*R_Date("UGAMS 39521", 3800, 20)*

*{*

*Outlier("General", 0.05);*

*};*

*R_Date("UGAMS 39520", 3750, 20)*

*{*

*Outlier("General", 0.05);*

*};*

*R_Date("UGAMS 39518", 3770, 20)*

*{*

*Outlier("General", 0.05);*

*};*

*R_Date("UGAMS_39517", 3810, 20)*

*{*

*Outlier();*

*};*

*R_Date("UGAMS 39516", 3710, 20)*

*{*

*Outlier("General", 0.05);*

*};*

*R_Date("UGAMS 39515", 3650, 20)*

*{*

*Outlier("General", 0.05);*

*};*

*Interval(LnN(ln(125),ln(2)));*

*};*

*};*

*Boundary("End: 9MC87");*

*};*

*Difference("Lapse","Start: 9MC87","End: 9MC87");*

*};*

***38CH12R1M1***

*Plot()*

*{*

*Sequence("38CH12 Ring")*

*{*

*Boundary("Start: 38CH12 Ring");*

*Phase("38CH12 Ring Units")*

*{*

*R_Date("UGA2905", 3345, 70);*

*R_Date("UGA2903", 3180, 65);*

*R_Date("UGA2904", 2885, 175);*

*R_Date("UGA2902", 3275, 55);*

*R_Date("UGA2901", 3190, 70);*

*Interval("Duration of 38CH12 Ring");*

*};*

*Boundary("End: 38CH12 Ring");*

*};*

*Difference("Lapse","Start: 38CH12 Ring ","End: 38CH12 Ring ");*

*};*

***38CH12R1M2***

*Plot()*

*{*

*Outlier_Model("General",T(5),U(0,4),"t");*

*Sequence("38CH12 Ring")*

*{*

*Boundary("Start: 38CH12 Ring");*

*Phase("38CH12 Ring Units")*

*{*

*R_Date("UGA2905", 3345, 70)*

*{*

*Outlier("General", 0.05);*

*};*

*R_Date("UGA2903", 3180, 65)*

*{*

*Outlier("General", 0.05);*

*};*

*R_Date("UGA2904", 2885, 175)*

*{*

*Outlier("General", 0.05);*

*};*

*R_Date("UGA2902", 3275, 55)*

*{*

*Outlier("General", 0.05);*

*};*

*R_Date("UGA2901", 3190, 70)*

*{*

*Outlier("General", 0.05);*

*};*

*Interval("Duration of 38CH12 Ring");*

*};*

*Boundary("End: 38CH12 Ring");*

*};*

*Difference("Lapse","Start: 38CH12 Ring ","End: 38CH12 Ring ");*

*};*

***38CH12R1M3***

*Plot()*

*{*

*Outlier_Model("Charcoal",Exp(1,-10,0),U(0,3),"t");*

*Sequence("38CH12 Ring")*

*{*

*Boundary("Start: 38CH12 Ring");*

*Phase("38CH12 Ring Units")*

*{*

*R_Date("UGA2905", 3345, 70)*

*{*

*Outlier("Charcoal", 1);*

*};*

*R_Date("UGA2903", 3180, 65)*

*{*

*Outlier("Charcoal", 1);*

*};*

*R_Date("UGA2904", 2885, 175)*

*{*

*Outlier("Charcoal", 1);*

*};*

*R_Date("UGA2902", 3275, 55)*

*{*

*Outlier("Charcoal", 1);*

*};*

*R_Date("UGA2901", 3190, 70)*

*{*

*Outlier("Charcoal", 1);*

*};*

*Interval("Duration of 38CH12 Ring");*

*};*

*Boundary("End: 38CH12 Ring");*

*};*

*Difference("Lapse","Start: 38CH12 Ring ","End: 38CH12 Ring ");*

*};*

***38CH12R1M4***

*Plot()*

*{*

*Outlier_Model("General",T(5),U(0,4),"t");*

*Sequence("38CH12 Ring")*

*{*

*Boundary("Start: 38CH12 Ring");*

*KDE_Plot("38CH12 Ring Units")*

*{*

*R_Date("UGA2905", 3345, 70)*

*{*

*Outlier("General", 0.05);*

*};*

*R_Date("UGA2903", 3180, 65)*

*{*

*Outlier("General", 0.05);*

*};*

*R_Date("UGA2904", 2885, 175)*

*{*

*Outlier("General", 0.05);*

*};*

*R_Date("UGA2902", 3275, 55)*

*{*

*Outlier("General", 0.05);*

*};*

*R_Date("UGA2901", 3190, 70)*

*{*

*Outlier("General", 0.05);*

*};*

*Interval(LnN(ln(125),ln(2)));*

*};*

*Boundary("End: 38CH12 Ring");*

*};*

*Difference("Lapse","Start: 38CH12 Ring","End: 38CH12 Ring");*

*};*

***38CH42R1M1***

*Plot()*

*{*

*Sequence("38CH42 Ring 1")*

*{*

*Boundary("Start: 38CH42 Ring 1");*

*Phase("38CH42 Ring 1 Units")*

*{*

*Sequence(“Fig 1C - Unit 2”)*

*{*

*R_Date("UGAMS 52717", 3590, 17);*

*R_Date("UGAMS 52716", 3589, 17);*

*R_Date("UGAMS 52715", 3575, 17);*

*R_Date("UGAMS 52714", 3606, 17);*

*};*

*R_Combine(“UGAMS 53619-Fig 1A-Unit 1”)*

*{*

*R_Date("UGAMS 53619a", 3510, 25);*

*R_Date("UGAMS 53619b", 3520, 25);*

*};*

*Interval("Duration of 38CH42 Ring 1");*

*};*

*Boundary("End: 38CH42 Ring 1");*

*};*

*Difference("Lapse","Start: 38CH42 Ring 1","End: 38CH42 Ring 1");*

*};*

***38CH42R1M2***

*Plot()*

*{*

*Outlier_Model("General",T(5),U(0,4),"t");*

*Sequence("38CH42 Ring 1")*

*{*

*Boundary("Start: 38CH42 Ring 1");*

*Phase("38CH42 Ring 1 Units")*

*{*

*Sequence(“Fig 1C - Unit 2”)*

*{*

*R_Date("UGAMS 52717", 3590, 17)*

*{*

*Outlier("General", 0.05);*

*};*

*R_Date("UGAMS 52716", 3589, 17)*

*{*

*Outlier("General", 0.05);*

*};*

*R_Date("UGAMS 52715", 3575, 17)*

*{*

*Outlier("General", 0.05);*

*};*

*R_Date("UGAMS 52714", 3606, 17)*

*{*

*Outlier("General", 0.05);*

*};*

*};*

*R_Combine(“UGAMS 53619-Fig 1A-Unit 1”)*

*{*

*R_Date("UGAMS 53619a", 3510, 25);*

*R_Date("UGAMS 53619b", 3520, 25);*

*};*

*};*

*Boundary("End: 38CH42 Ring 1");*

*};*

*Difference("Lapse","Start: 38CH42 Ring 1","End: 38CH42 Ring 1");*

*};*

***38CH42R1M3***

*Plot()*

*{*

*Sequence("38CH42 Ring 1")*

*{*

*Boundary("Start: 38CH42 Ring 1");*

*KDE_Plot("38CH42 Ring 1 Units")*

*{*

*Sequence("Fig 1C - Unit 2")*

*{*

*R_Date("UGAMS 52717", 3590, 17);*

*R_Date("UGAMS 52716", 3589, 17);*

*R_Date("UGAMS 52715", 3575, 17);*

*R_Date("UGAMS 52714", 3606, 17);*

*};*

*R_Combine("UGAMS 53619-Fig 1A-Unit 1")*

*{*

*R_Date("UGAMS 53619a", 3510, 25);*

*R_Date("UGAMS 53619b", 3520, 25);*

*};*

*Interval(LnN(ln(125),ln(2)));*

*};*

*Boundary("End: 38CH42 Ring 1");*

*};*

*Difference("Lapse","Start: 38CH42 Ring 1","End: 38CH42 Ring 1");*

*};*

***38CH42R2M1***

*Plot()*

*{*

*Sequence("38CH42 Ring 2")*

*{*

*Boundary("Start: 38CH42 Ring 2");*

*Phase("38CH42 Ring 2 Units")*

*{*

*R_Date("UGAMS 52719-ST 4", 3750, 23);*

*Sequence(“Quadrat 201”)*

*{*

*R_Combine(“UGAMS 53618”)*

*{*

*R_Date("UGAMS 53618a", 3660, 25);*

*R_Date("UGAMS 53618b", 3690, 25);*

*};*

*R_Combine(“UGAMS 53617”)*

*{*

*R_Date("UGAMS 53617a", 3660, 25);*

*R_Date("UGAMS 53617b", 3690, 30);*

*};*

*};*

*};*

*Boundary("End: 38CH42 Ring 2");*

*};*

*Difference("Lapse","Start: 38CH42 Ring 2","End: 38CH42 Ring 2");*

*};*

***38CH42R2M2***

*Plot()*

*{*

*Outlier_Model("General",T(5),U(0,4),"t");*

*Sequence("38CH42 Ring 2")*

*{*

*Boundary("Start: 38CH42 Ring 2");*

*Phase("38CH42 Ring 2 Units")*

*{*

*R_Date("UGAMS 52719-ST 4", 3750, 23)*

*{*

*Outlier("General", 0.05);*

*};*

*Sequence(“Quadrat 201”)*

*{*

*R_Combine(“UGAMS 53618”)*

*{*

*R_Date("UGAMS 53618a", 3660, 25);*

*R_Date("UGAMS 53618b", 3690, 25);*

*};*

*R_Combine(“UGAMS 53617”)*

*{*

*R_Date("UGAMS 53617a", 3660, 25);*

*R_Date("UGAMS 53617b", 3690, 30);*

*};*

*};*

*};*

*Boundary("End: 38CH42 Ring 2");*

*};*

*Difference("Lapse","Start: 38CH42 Ring 2","End: 38CH42 Ring 2");*

*};*

***38CH42R2M3***

*Plot()*

*{*

*Sequence("38CH42 Ring 2")*

*{*

*Boundary("Start: 38CH42 Ring 2");*

*KDE_Plot("38CH42 Ring 2 Units")*

*{*

*R_Date("UGAMS 52719-ST 4", 3750, 23);*

*Sequence("Quadrat 201")*

*{*

*R_Combine("UGAMS 53618")*

*{*

*R_Date("UGAMS 53618a", 3660, 25);*

*R_Date("UGAMS 53618b", 3690, 25);*

*};*

*R_Combine("UGAMS 53617")*

*{*

*R_Date("UGAMS 53617a", 3660, 25);*

*R_Date("UGAMS 53617b", 3690, 30);*

*};*

*};*

*Interval(LnN(ln(125),ln(2)));*

*};*

*Boundary("End: 38CH42 Ring 2");*

*};*

*Difference("Lapse","Start: 38CH42 Ring 2","End: 38CH42 Ring 2");*

*};*

***38CH42R3M1***

*Plot()*

*{*

*Sequence("38CH42 Ring 3")*

*{*

*Boundary("Start: 38CH42 Ring 3");*

*Phase("38CH42 Ring 3 Units")*

*{*

*Sequence(“Unit 2”)*

*{*

*R_Date("UGAMS 52712", 3750, 17);*

*R_Date("UGAMS 52711", 3740, 23);*

*};*

*R_Date("UGAMS 52713-Unit 5", 3780, 17);*

*Interval("Duration of 38CH42 Ring 3");*

*};*

*Boundary("End: 38CH42 Ring 3");*

*};*

*Difference("Lapse","Start: 38CH42 Ring 3","End: 38CH42 Ring 3");*

*};*

***38CH42R3M2***

*Plot()*

*{*

*Outlier_Model("General",T(5),U(0,4),"t");*

*Sequence("38CH42 Ring 3")*

*{*

*Boundary("Start: 38CH42 Ring 3");*

*Phase("38CH42 Ring 3 Units")*

*{*

*Sequence(“Unit 2”)*

*{*

*R_Date("UGAMS 52712", 3750, 17)*

*{*

*Outlier("General", 0.05);*

*};*

*R_Date("UGAMS 52711", 3740, 23)*

*{*

*Outlier("General", 0.05);*

*};*

*};*

*R_Date("UGAMS 52713-Unit 5", 3780, 17)*

*{*

*Outlier("General", 0.05);*

*};*

*};*

*Boundary("End: 38CH42 Ring 3");*

*};*

*Difference("Lapse","Start: 38CH42 Ring 3","End: 38CH42 Ring 3");*

*};*

***38CH42R3M3***

*Plot()*

*{*

*Sequence("38CH42 Ring 3")*

*{*

*Boundary("Start: 38CH42 Ring 3");*

*KDE_Plot("38CH42 Ring 3 Units")*

*{*

*Sequence("Unit 2")*

*{*

*R_Date("UGAMS 52712", 3750, 17);*

*R_Date("UGAMS 52711", 3740, 23);*

*};*

*R_Date("UGAMS 52713-Unit 5", 3780, 17);*

*Interval(LnN(ln(125),ln(2)));*

*};*

*Boundary("End: 38CH42 Ring 3");*

*};*

*Difference("Lapse","Start: 38CH42 Ring 3","End: 38CH42 Ring 3");*

*};*

***38CH62R1M1***

*Plot()*

*{*

*Sequence("38CH62 Mound")*

*{*

*After("Charcoal TPQ Below Shell")*

*{*

*R_Date("UGAMS 59261", 5040, 25);*

*};*

*Boundary("Start: 38CH62 Mound");*

*Phase(“38CH62 Mound Units")*

*{*

*Sequence(“Unit 6”)*

*{*

*R_Date("Beta - 459730", 3790, 30);*

*R_Date("Beta - 489680", 3570, 30);*

*R_Date("Beta - 459728", 3500, 30);*

*};*

*Sequence(“Unit 7”)*

*{*

*R_Date("UGAMS 59263", 3670, 25);*

*R_Date("UGAMS 59262", 3620, 25);*

*R_Date("Beta - 489681", 3580, 30);*

*R_Date("Beta - 465413", 3600, 30);*

*R_Date("Beta - 465411", 3590, 30);*

*};*

*R_Date("Beta - 517654-12H", 3640, 30);*

*R_Combine(“16I”)*

*{*

*R_Date("Beta - 517655", 3620, 30);*

*R_Date("OxA - 41058", 3679, 18);*

*R_Date("OxA - 41059", 3657, 18);*

*};*

*R_Date("Beta - 465417-8J", 3640, 30);*

*R_Combine(“9G”)*

*{*

*R_Date("OxA - 41060", 3645, 18);*

*R_Date("Beta - 517656", 3620, 30);*

*};*

*R_Date("Beta - 517653-10H", 3580, 30);*

*};*

*Boundary("End: 38CH62 Mound");*

*};*

*Difference("Lapse","Start: 38CH62 Mound","End: 38CH62 Mound");*

*};*

***38CH62R1M2***

*Plot()*

*{*

*Outlier_Model("General",T(5),U(0,4),"t");*

*Sequence("38CH62 Mound")*

*{*

*After("Charcoal TPQ Below Shell")*

*{*

*R_Date("UGAMS 59261", 5040, 25)*

*{*

*Outlier("General", 0.05);*

*};*

*};*

*Boundary("Start: 38CH62 Mound");*

*Phase(“38CH62 Mound Units")*

*{*

*Sequence(“Unit 6”)*

*{*

*R_Date("Beta - 459730", 3790, 30)*

*{*

*Outlier("General", 0.05);*

*};*

*R_Date("Beta - 489680", 3570, 30)*

*{*

*Outlier("General", 0.05);*

*};*

*R_Date("Beta - 459728", 3500, 30)*

*{*

*Outlier("General", 0.05);*

*};*

*};*

*Sequence(“Unit 7”)*

*{*

*R_Date("UGAMS 59263", 3670, 25)*

*{*

*Outlier("General", 0.05);*

*};*

*R_Date("UGAMS 59262", 3620, 25)*

*{*

*Outlier("General", 0.05);*

*};*

*R_Date("Beta - 489681", 3580, 30)*

*{*

*Outlier("General", 0.05);*

*};*

*R_Date("Beta - 465413", 3600, 30)*

*{*

*Outlier("General", 0.05);*

*};*

*R_Date("Beta - 465411", 3590, 30)*

*{*

*Outlier("General", 0.05);*

*};*

*};*

*R_Date("Beta - 517654-12H", 3640, 30)*

*{*

*Outlier("General", 0.05);*

*};*

*R_Combine(“16I”)*

*{*

*R_Date("Beta - 517655", 3620, 30);*

*R_Date("OxA - 41058", 3679, 18);*

*R_Date("OxA - 41059", 3657, 18);*

*};*

*R_Date("Beta - 465417-8J", 3640, 30)*

*{*

*Outlier("General", 0.05);*

*};*

*R_Combine(“9G”)*

*{*

*R_Date("OxA - 41060", 3645, 18);*

*R_Date("Beta - 517656", 3620, 30);*

*};*

*R_Date("Beta - 517653-10H", 3580, 30)*

*{*

*Outlier("General", 0.05);*

*};*

*};*

*Boundary("End: 38CH62 Mound");*

*};*

*Difference("Lapse","Start: 38CH62 Mound","End: 38CH62 Mound");*

*};*

***38CH62R1M3***

*Plot()*

*{*

*Outlier_Model("Charcoal",Exp(1,-10,0),U(0,3),"t");*

*Sequence("38CH62 Mound")*

*{*

*After("Charcoal TPQ Below Shell")*

*{*

*R_Date("UGAMS 59261", 5040, 25)*

*{*

*Outlier("Charcoal", 1);*

*}*

*};*

*Boundary("Start: 38CH62 Mound");*

*Phase(“38CH62 Mound Units")*

*{*

*Sequence(“Unit 6”)*

*{*

*R_Date("Beta - 459730", 3790, 30)*

*{*

*Outlier("Charcoal", 1);*

*};*

*R_Date("Beta - 489680", 3570, 30);*

*R_Date("Beta - 459728", 3500, 30)*

*{*

*Outlier("Charcoal", 1);*

*};*

*};*

*Sequence(“Unit 7”)*

*{*

*R_Date("UGAMS 59263", 3670, 25);*

*R_Date("UGAMS 59262", 3620, 25);*

*R_Date("Beta - 489681", 3580, 30)*

*{*

*Outlier("Charcoal", 1);*

*};*

*R_Date("Beta - 465413", 3600, 30)*

*{*

*Outlier("Charcoal", 1);*

*};*

*R_Date("Beta - 465411", 3590, 30)*

*{*

*Outlier("Charcoal", 1);*

*};*

*};*

*R_Date("Beta - 517654-12H", 3640, 30);*

*R_Combine(“16I”)*

*{*

*R_Date("Beta - 517655", 3620, 30);*

*R_Date("OxA - 41058", 3679, 18);*

*R_Date("OxA - 41059", 3657, 18);*

*};*

*R_Date("Beta - 465417-8J", 3640, 30)*

*{*

*Outlier("Charcoal", 1);*

*};*

*R_Combine(“9G”)*

*{*

*R_Date("OxA - 41060", 3645, 18);*

*R_Date("Beta - 517656", 3620, 30);*

*};*

*R_Date("Beta - 517653-10H", 3580, 30);*

*};*

*Boundary("End: 38CH62 Mound");*

*};*

*Difference("Lapse","Start: 38CH62 Mound","End: 38CH62 Mound");*

*};*

***38CH62R1M4***

*Plot()*

*{*

*Outlier_Model("General",T(5),U(0,4),"t");*

*Sequence("38CH62 Mound")*

*{*

*After("Charcoal TPQ Below Shell")*

*{*

*R_Date("UGAMS 59261", 5040, 25)*

*{*

*Outlier("Charcoal", 1);*

*};*

*};*

*Boundary("Start: 38CH62 Mound");*

*Phase(“38CH62 Mound Units")*

*{*

*Sequence(“Unit 6”)*

*{*

*R_Date("Beta - 459730", 3790, 30)*

*{*

*Outlier("Charcoal", 1);*

*};*

*R_Date("Beta - 489680", 3570, 30)*

*{*

*Outlier("General", 0.05);*

*};*

*R_Date("Beta - 459728", 3500, 30)*

*{*

*Outlier("Charcoal", 1);*

*};*

*};*

*Sequence(“Unit 7”)*

*{*

*R_Date("UGAMS 59263", 3670, 25)*

*{*

*Outlier("General", 0.05);*

*};*

*R_Date("UGAMS 59262", 3620, 25)*

*{*

*Outlier("General", 0.05);*

*};*

*R_Date("Beta - 489681", 3580, 30)*

*{*

*Outlier("Charcoal", 1);*

*};*

*R_Date("Beta - 465413", 3600, 30)*

*{*

*Outlier("Charcoal", 1);*

*};*

*R_Date("Beta - 465411", 3590, 30)*

*{*

*Outlier("Charcoal", 1);*

*};*

*};*

*R_Date("Beta - 517654-12H", 3640, 30)*

*{*

*Outlier("General", 0.05);*

*};*

*R_Combine(“16I”)*

*{*

*R_Date("Beta - 517655", 3620, 30);*

*R_Date("OxA - 41058", 3679, 18);*

*R_Date("OxA - 41059", 3657, 18);*

*};*

*R_Date("Beta - 465417-8J", 3640, 30)*

*{*

*Outlier("Charcoal", 1);*

*};*

*R_Combine(“9G”)*

*{*

*R_Date("OxA - 41060", 3645, 18);*

*R_Date("Beta - 517656", 3620, 30);*

*};*

*R_Date("Beta - 517653-10H", 3580, 30)*

*{*

*Outlier("General", 0.05);*

*};*

*};*

*Boundary("End: 38CH62 Mound");*

*};*

*Difference("Lapse","Start: 38CH62 Mound","End: 38CH62 Mound");*

*};*

***38CH62R1M5***

*Plot()*

*{*

*Outlier_Model("Charcoal",Exp(1,-10,0),U(0,3),"t");*

*Sequence("38CH62 Mound")*

*{*

*After("Charcoal TPQ Below Shell")*

*{*

*R_Date("UGAMS 59261", 5040, 25)*

*{*

*Outlier("Charcoal", 1);*

*};*

*};*

*Boundary("Start: 38CH62 Mound");*

*KDE_Plot(38CH62 Units)*

*{*

*Phase("38CH62 Mound Units")*

*{*

*Sequence("Unit 6")*

*{*

*R_Date("Beta - 459730", 3790, 30)*

*{*

*Outlier("Charcoal", 1);*

*};*

*R_Date("Beta - 489680", 3570, 30);*

*R_Date("Beta - 459728", 3500, 30)*

*{*

*Outlier("Charcoal", 1);*

*};*

*};*

*Sequence("Unit 7")*

*{*

*R_Date("UGAMS 59263", 3670, 25);*

*R_Date("UGAMS 59262", 3620, 25);*

*R_Date("Beta - 489681", 3580, 30)*

*{*

*Outlier("Charcoal", 1);*

*};*

*R_Date("Beta - 465413", 3600, 30)*

*{*

*Outlier("Charcoal", 1);*

*};*

*R_Date("Beta - 465411", 3590, 30)*

*{*

*Outlier("Charcoal", 1);*

*};*

*};*

*R_Date("Beta - 517654-12H", 3640, 30);*

*R_Combine("16I")*

*{*

*R_Date("Beta - 517655", 3620, 30);*

*R_Date("OxA - 41058", 3679, 18);*

*R_Date("OxA - 41059", 3657, 18);*

*};*

*R_Date("Beta - 465417-8J", 3640, 30)*

*{*

*Outlier("Charcoal", 1);*

*};*

*R_Combine("9G")*

*{*

*R_Date("OxA - 41060", 3645, 18);*

*R_Date("Beta - 517656", 3620, 30);*

*};*

*R_Date("Beta - 517653-10H", 3580, 30);*

*Interval(LnN(ln(125),ln(2)));*

*};*

*};*

*Boundary("End: 38CH62 Mound");*

*};*

*Difference("Lapse","Start: 38CH62 Mound","End: 38CH62 Mound");*

*};*

***38CH1781AAM1***

*Plot()*

*{*

*Sequence("38CH1781 Area A")*

*{*

*Boundary("Start: 38CH1781 Area A");*

*Phase ("38CH1781 Area A Block A1 Units")*

*{*

*R_Date("Beta - 178546-Unit 407", 4090, 40);*

*R_Date("UGAMS 61229-Unit 411", 3950, 25);*

*Sequence("Unit 412")*

*{*

*R_Date("UGAMS 61232", 4040, 25);*

*R_Date("UGAMS 61231", 3920, 25);*

*R_Date("UGAMS 61230", 4000, 25);*

*};*

*};*

*Boundary("End: 38CH1781 Area A");*

*};*

*Difference("Lapse","Start: 38CH1781 Area A","End: 38CH1781 Area A");*

*};*

***38CH1781AAM2***

*Plot()*

*{*

*Outlier_Model("General",T(5),U(0,4),"t");*

*Sequence("38CH1781 Area A")*

*{*

*Boundary("Start: 38CH1781 Area A");*

*Phase ("38CH1781 Area A Block A1 Units")*

*{*

*R_Date("Beta - 178546-Unit 407", 4090, 40)*

*{*

*Outlier("General", 0.05);*

*};*

*R_Date("UGAMS 61229-Unit 411", 3950, 25)*

*{*

*Outlier("General", 0.05);*

*};*

*Sequence("Unit 412")*

*{*

*R_Date("UGAMS 61232", 4040, 25)*

*{*

*Outlier("General", 0.05);*

*};*

*R_Date("UGAMS 61231", 3920, 25)*

*{*

*Outlier("General", 0.05);*

*};*

*R_Date("UGAMS 61230", 4000, 25)*

*{*

*Outlier("General", 0.05);*

*};*

*};*

*};*

*Boundary("End: 38CH1781 Area A");*

*};*

*Difference("Lapse","Start: 38CH1781 Area A","End: 38CH1781 Area A");*

*};*

***38CH1781AAM3***

*Plot()*

*{*

*Outlier_Model("Charcoal",Exp(1,-10,0),U(0,3),"t");*

*Sequence("38CH1781 Area A")*

*{*

*Boundary("Start: 38CH1781 Area A");*

*Phase ("38CH1781 Area A Block A1 Units")*

*{*

*R_Date("Beta - 178546-Unit 407", 4090, 40)*

*{*

*Outlier("Charcoal", 1);*

*};*

*R_Date("UGAMS 61229-Unit 411", 3950, 25);*

*Sequence("Unit 412")*

*{*

*R_Date("UGAMS 61232", 4040, 25);*

*R_Date("UGAMS 61231", 3920, 25);*

*R_Date("UGAMS 61230", 4000, 25);*

*};*

*};*

*Boundary("End: 38CH1781 Area A");*

*};*

*Difference("Lapse","Start: 38CH1781 Area A","End: 38CH1781 Area A");*

*};*

***38CH1781AAM4***

*Plot()*

*{*

*Outlier_Model("General",T(5),U(0,4),"t");*

*Outlier_Model("Charcoal",Exp(1,-10,0),U(0,3),"t");*

*Sequence("38CH1781 Area A")*

*{*

*Boundary("Start: 38CH1781 Area A");*

*Phase ("38CH1781 Area A Block A1 Units")*

*{*

*R_Date("Beta - 178546-Unit 407", 4090, 40)*

*{*

*Outlier("Charcoal", 1);*

*};*

*R_Date("UGAMS 61229-Unit 411", 3950, 25)*

*{*

*Outlier("General", 0.05);*

*};*

*Sequence("Unit 412")*

*{*

*R_Date("UGAMS 61232", 4040, 25)*

*{*

*Outlier("General", 0.05);*

*};*

*R_Date("UGAMS 61231", 3920, 25)*

*{*

*Outlier();*

*};*

*R_Date("UGAMS 61230", 4000, 25)*

*{*

*Outlier("General", 0.05);*

*};*

*};*

*};*

*Boundary("End:38CH1781 Area A");*

*};*

*Difference("Lapse","Start: 38CH1781 Area A","End: 38CH1781 Area A");*

*};*

***38CH1781AAM5***

*Plot()*

*{*

*Outlier_Model("General",T(5),U(0,4),"t");*

*Outlier_Model("Charcoal",Exp(1,-10,0),U(0,3),"t");*

*Sequence("38CH1781 Area A")*

*{*

*Boundary("Start: 38CH1781 Area A");*

*KDE_PLot("38CH1781 Area A Block A1 Units")*

*{*

*R_Date("Beta - 178546-Unit 407", 4090, 40)*

*{*

*Outlier("Charcoal", 1);*

*};*

*R_Date("UGAMS 61229-Unit 411", 3950, 25)*

*{*

*Outlier("General", 0.05);*

*};*

*Sequence("Unit 412")*

*{*

*R_Date("UGAMS 61232", 4040, 25)*

*{*

*Outlier("General", 0.05);*

*};*

*R_Date("UGAMS 61231", 3920, 25)*

*{*

*Outlier();*

*};*

*R_Date("UGAMS 61230", 4000, 25)*

*{*

*Outlier("General", 0.05);*

*};*

*};*

*Interval(LnN(ln(125),ln(2)));*

*};*

*Boundary("End:38CH1781 Area A");*

*};*

*Difference("Lapse","Start: 38CH1781 Area A","End: 38CH1781 Area A");*

*};*

***38CH1781ADM1***

*Plot()*

*{*

*Sequence("38CH1781 Area D")*

*{*

*Boundary("Start: 38CH1781 Area D");*

*Phase ("38CH1781 Area D Units")*

*{*

*Sequence("Unit 452, Midden 2")*

*{*

*R_Date("UGAMS 61235", 3940, 25);*

*R_Date("Beta - 291351", 4060, 40);*

*R_Date("UGAMS 61234", 3970, 25);*

*R_Date("UGAMS 61233", 4020, 25);*

*};*

*Sequence("Unit 462, Midden 3")*

*{*

*R_Date("UGAMS 61237", 3950, 25);*

*R_Date("UGAMS 61236", 3950, 25);*

*};*

*};*

*Boundary("End: 38CH1781 Area D");*

*};*

*Difference("Lapse","Start: 38CH1781 Area D","End: 38CH1781 Area D");*

*};*

***38CH1781ADM2***

*Plot()*

*{*

*Outlier_Model("General",T(5),U(0,4),"t");*

*Sequence("38CH1781 Area D")*

*{*

*Boundary("Start: 38CH1781 Area D");*

*Phase ("38CH1781 Area D Units")*

*{*

*Sequence("Unit 452, Midden 2")*

*{*

*R_Date("UGAMS 61235", 3940, 25)*

*{*

*Outlier("General", 0.05);*

*};*

*R_Date("Beta - 291351", 4060, 40)*

*{*

*Outlier("General", 0.05);*

*};*

*R_Date("UGAMS 61234", 3970, 25)*

*{*

*Outlier("General", 0.05);*

*};*

*R_Date("UGAMS 61233", 4020, 25)*

*{*

*Outlier("General", 0.05);*

*};*

*};*

*Sequence("Unit 462, Midden 3")*

*{*

*R_Date("UGAMS 61237", 3950, 25)*

*{*

*Outlier("General", 0.05);*

*};*

*R_Date("UGAMS 61236", 3950, 25)*

*{*

*Outlier("General", 0.05);*

*};*

*};*

*};*

*Boundary("End: 38CH1781 Area D");*

*};*

*Difference("Lapse","Start: 38CH1781 Area D","End: 38CH1781 Area D");*

*};*

***38CH1781ADM3***

*Plot()*

*{*

*Outlier_Model("General",T(5),U(0,4),"t");*

*Sequence("38CH1781 Area D")*

*{*

*Boundary("Start: 38CH1781 Area D");*

*KDE_Plot("38CH1781 Area D Units")*

*{*

*Sequence("Unit 452, Midden 2")*

*{*

*R_Date("UGAMS 61235", 3940, 25)*

*{*

*Outlier("General", 0.05);*

*};*

*R_Date("Beta - 291351", 4060, 40)*

*{*

*Outlier("General", 0.05);*

*};*

*R_Date("UGAMS 61234", 3970, 25)*

*{*

*Outlier("General", 0.05);*

*};*

*R_Date("UGAMS 61233", 4020, 25)*

*{*

*Outlier("General", 0.05);*

*};*

*};*

*Sequence("Unit 462, Midden 3")*

*{*

*R_Date("UGAMS 61237", 3950, 25)*

*{*

*Outlier("General", 0.05);*

*};*

*R_Date("UGAMS 61236", 3950, 25)*

*{*

*Outlier("General", 0.05);*

*};*

*};*

*Interval(LnN(ln(125),ln(2)));*

*};*

*Boundary("End: 38CH1781 Area D");*

*};*

*Difference("Lapse","Start: 38CH1781 Area D","End: 38CH1781 Area D");*

*};*

***38CH2533R1M1***

*Plot()*

*{*

*Sequence("38CH2533 Ring 1")*

*{*

*After("OSL TPQ Below Shell")*

*{*

*Date("OSL-BG4838",N(2010-4970,240));*

*};*

*Boundary("Start: 38CH2533 Ring 1");*

*Phase("38CH2533 Ring 1 Units")*

*{*

*Sequence("Quadrat 1")*

*{*

*R_Date("AA 110474", 3912, 33);*

*R_Date("AA 110475", 3863, 33);*

*};*

*Sequence("Quadrat 25")*

*{*

*R_Date("UGAMS 53611-25I", 3820, 25);*

*R_Date("UGAMS 53610-25H", 3790, 25);*

*};*

*R_Date("UGAMS 41245r-9H", 3880, 25);*

*R_Combine("38CH2533-24H")*

*{*

*R_Date("AA 113826", 3858, 45);*

*R_Date("UGAMS 51563a", 3880, 23);*

*R_Date("UGAMS 51563b", 3880, 23);*

*};*

*Sequence("Quadrat 23")*

*{*

*R_Combine("38CH2533-23F")*

*{*

*R_Date("AA 113824", 3880, 45);*

*R_Date("UGAMS 51561a", 3830, 23);*

*R_Date("UGAMS 51561b", 3890, 23);*

*};*

*R_Combine("38CH2533-23E")*

*{*

*R_Date("AA 113825", 3812, 45);*

*R_Date("OxA-40301", 3872, 20);*

*R_Date("UGAMS 51562a", 3850, 23);*

*R_Date("UGAMS 51562b", 3830, 23);*

*};*

*};*

*R_Date("UGAMS 41244r-8E", 3850, 25);*

*R_Date("UGAMS 41243r-3D", 3810, 25);*

*R_Combine("38CH2533-4D")*

*{*

*R_Date("AA 113823", 3813, 45);*

*R_Date("OxA - 40302", 3851, 20);*

*R_Date("UGAMS 51560a", 3840, 24);*

*R_Date("UGAMS 51560b", 3810, 23);*

*};*

*R_Date("UGAMS 52720-6D", 3850, 23);*

*R_Date("UGAMS 59253-29G", 3800, 25);*

*R_Date("AA 110472-N2040E1150", 3936, 33);*

*R_Date("AA 110476-N2050E1170", 3896, 33);*

*R_Date("AA 110471-N2060E1140", 3881, 30);*

*R_Date("AA 110473-N2060E1170", 3892, 33);*

*Interval("Duration of 38CH2533 Ring 1");*

*};*

*Boundary("End: 38CH2533 Ring 1");*

*};*

*Difference("Lapse","Start: 38CH2533 Ring 1","End: 38CH2533 Ring 1");*

*};*

***38CH2533R1M2***

*Plot()*

*{*

*Outlier_Model("General",T(5),U(0,4),"t");*

*Sequence("38CH2533 Ring 1")*

*{*

*After("OSL TPQ Below Shell")*

*{*

*Date("OSL-BG4838",N(2010-4970,240));*

*};*

*Boundary("Start: 38CH2533 Ring 1");*

*Phase("38CH2533 Ring 1 Units")*

*{*

*Sequence("Quadrat 1")*

*{*

*R_Date("AA 110474", 3912, 33)*

*{*

*Outlier("General", 0.05);*

*};*

*R_Date("AA 110475", 3863, 33)*

*{*

*Outlier("General", 0.05);*

*};*

*};*

*Sequence("Quadrat 25")*

*{*

*R_Date("UGAMS 53611-25I", 3820, 25)*

*{*

*Outlier("General", 0.05);*

*};*

*R_Date("UGAMS 53610-25H", 3790, 25)*

*{*

*Outlier("General", 0.05);*

*};*

*};*

*R_Date("UGAMS 41245r-9H", 3880, 25)*

*{*

*Outlier("General", 0.05);*

*};*

*R_Combine("38CH2533-24H")*

*{*

*R_Date("AA 113826", 3858, 45);*

*R_Date("UGAMS 51563a", 3880, 23);*

*R_Date("UGAMS 51563b", 3880, 23);*

*};*

*Sequence("Quadrat 23")*

*{*

*R_Combine("38CH2533-23F")*

*{*

*R_Date("AA 113824", 3880, 45);*

*R_Date("UGAMS 51561a", 3830, 23);*

*R_Date("UGAMS 51561b", 3890, 23);*

*};*

*R_Combine("38CH2533-23E")*

*{*

*R_Date("AA 113825", 3812, 45);*

*R_Date("OxA-40301", 3872, 20);*

*R_Date("UGAMS 51562a", 3850, 23);*

*R_Date("UGAMS 51562b", 3830, 23);*

*};*

*};*

*R_Date("UGAMS 41244r-8E", 3850, 25)*

*{*

*Outlier("General", 0.05);*

*};*

*R_Date("UGAMS 41243r-3D", 3810, 25)*

*{*

*Outlier("General", 0.05);*

*};*

*R_Combine("38CH2533-4D")*

*{*

*R_Date("AA 113823", 3813, 45);*

*R_Date("OxA - 40302", 3851, 20);*

*R_Date("UGAMS 51560a", 3840, 24);*

*R_Date("UGAMS 51560b", 3810, 23);*

*};*

*R_Date("UGAMS 52720-6D", 3850, 23)*

*{*

*Outlier("General", 0.05);*

*};*

*R_Date("UGAMS 59253-29G", 3800, 25)*

*{*

*Outlier("General", 0.05);*

*};*

*R_Date("AA 110472-N2040E1150", 3936, 33)*

*{*

*Outlier("General", 0.05);*

*};*

*R_Date("AA 110476-N2050E1170", 3896, 33)*

*{*

*Outlier("General", 0.05);*

*};*

*R_Date("AA 110471-N2060E1140", 3881, 30)*

*{*

*Outlier("General", 0.05);*

*};*

*R_Date("AA 110473-N2060E1170", 3892, 33)*

*{*

*Outlier("General", 0.05);*

*};*

*};*

*Boundary("End: 38CH2533 Ring 1");*

*};*

*Difference("Lapse","Start: 38CH2533 Ring 1","End: 38CH2533 Ring 1");*

*};*

***38CH2533R1M3***

*Plot()*

*{*

*Outlier_Model("General",T(5),U(0,4),"t");*

*Sequence("38CH2533 Ring 1")*

*{*

*After("OSL TPQ Below Shell")*

*{*

*Date("OSL-BG4838",N(2010-4970,240));*

*};*

*Boundary("Start: 38CH2533 Ring 1");*

*KDE_Plot("38CH2533 Ring 1 Units")*

*{*

*Sequence("Quadrat 1")*

*{*

*R_Date("AA 110474", 3912, 33)*

*{*

*Outlier("General", 0.05);*

*};*

*R_Date("AA 110475", 3863, 33)*

*{*

*Outlier("General", 0.05);*

*};*

*};*

*Sequence("Quadrat 25")*

*{*

*R_Date("UGAMS 53611-25I", 3820, 25)*

*{*

*Outlier("General", 0.05);*

*};*

*R_Date("UGAMS 53610-25H", 3790, 25)*

*{*

*Outlier("General", 0.05);*

*};*

*};*

*R_Date("UGAMS 41245r-9H", 3880, 25)*

*{*

*Outlier("General", 0.05);*

*};*

*R_Combine("38CH2533-24H")*

*{*

*R_Date("AA 113826", 3858, 45);*

*R_Date("UGAMS 51563a", 3880, 23);*

*R_Date("UGAMS 51563b", 3880, 23);*

*};*

*Sequence("Quadrat 23")*

*{*

*R_Combine("38CH2533-23F")*

*{*

*R_Date("AA 113824", 3880, 45);*

*R_Date("UGAMS 51561a", 3830, 23);*

*R_Date("UGAMS 51561b", 3890, 23);*

*};*

*R_Combine("38CH2533-23E")*

*{*

*R_Date("AA 113825", 3812, 45);*

*R_Date("OxA-40301", 3872, 20);*

*R_Date("UGAMS 51562a", 3850, 23);*

*R_Date("UGAMS 51562b", 3830, 23);*

*};*

*};*

*R_Date("UGAMS 41244r-8E", 3850, 25)*

*{*

*Outlier("General", 0.05);*

*};*

*R_Date("UGAMS 41243r-3D", 3810, 25)*

*{*

*Outlier("General", 0.05);*

*};*

*R_Combine("38CH2533-4D")*

*{*

*R_Date("AA 113823", 3813, 45);*

*R_Date("OxA - 40302", 3851, 20);*

*R_Date("UGAMS 51560a", 3840, 24);*

*R_Date("UGAMS 51560b", 3810, 23);*

*};*

*R_Date("UGAMS 52720-6D", 3850, 23)*

*{*

*Outlier("General", 0.05);*

*};*

*R_Date("UGAMS 59253-29G", 3800, 25)*

*{*

*Outlier("General", 0.05);*

*};*

*R_Date("AA 110472-N2040E1150", 3936, 33)*

*{*

*Outlier("General", 0.05);*

*};*

*R_Date("AA 110476-N2050E1170", 3896, 33)*

*{*

*Outlier("General", 0.05);*

*};*

*R_Date("AA 110471-N2060E1140", 3881, 30)*

*{*

*Outlier("General", 0.05);*

*};*

*R_Date("AA 110473-N2060E1170", 3892, 33)*

*{*

*Outlier("General", 0.05);*

*};*

*Interval(LnN(ln(125),ln(2)));*

*};*

*Boundary("End: 38CH2533 Ring 1");*

*};*

*Difference("Lapse","Start: 38CH2533 Ring 1","End: 38CH2533 Ring 1");*

*};*

***38CH2533R2M1***

*Plot()*

*{*

*Sequence(“38CH2533 Ring 2”)*

*{*

*Boundary("Start: 38CH2533 Ring 2");*

*Phase("38CH2533 Ring 2 Units")*

*{*

*Sequence(“Quadrat 110”)*

*{*

*R_Date("UGAMS 53615", 3830, 25);*

*R_Date("UGAMS 53616", 3780, 25);*

*R_Date("OxA - 41061", 3854, 19);*

*R_Date("UGAMS 53614", 3840, 25);*

*R_Date("UGAMS 53613", 3730, 25);*

*R_Combine("UGAMS 53612")*

*{*

*R_Date("UGAMS 53612a", 3760, 25);*

*R_Date("UGAMS 53612b", 3700, 25);*

*};*

*};*

*R_Date("OxA - 41062-N2140E1020", 3871, 19);*

*Interval("Duration of 38CH2533 Ring 2");*

*};*

*Boundary("End: 38CH2533 Ring 2");*

*};*

*Difference("Lapse","Start: 38CH2533 Ring 2","End: 38CH2533 Ring 2");*

*};*

***38CH2533R2M2***

*Plot()*

*{*

*Outlier_Model("General",T(5),U(0,4),"t");*

*Sequence(“38CH2533 Ring 2”)*

*{*

*Boundary("Start: 38CH2533 Ring 2");*

*Phase("38CH2533 Ring 2 Units")*

*{*

*Sequence(“Quadrat 110”)*

*{*

*R_Date("UGAMS 53615", 3830, 25)*

*{*

*Outlier("General", 0.05);*

*};*

*R_Date("UGAMS 53616", 3780, 25)*

*{*

*Outlier("General", 0.05);*

*};*

*R_Date("OxA - 41061", 3854, 19)*

*{*

*Outlier("General", 0.05);*

*};*

*R_Date("UGAMS 53614", 3840, 25)*

*{*

*Outlier("General", 0.05);*

*};*

*R_Date("UGAMS 53613", 3730, 25)*

*{*

*Outlier("General", 0.05);*

*};*

*R_Combine("UGAMS 53612")*

*{*

*R_Date("UGAMS 53612a", 3760, 25);*

*R_Date("UGAMS 53612b", 3700, 25);*

*};*

*};*

*R_Date("OxA - 41062-N2140E1020", 3871, 19)*

*{*

*Outlier("General", 0.05);*

*};*

*};*

*Boundary("End: 38CH2533 Ring 2");*

*};*

*Difference("Lapse","Start: 38CH2533 Ring 2","End: 38CH2533 Ring 2");*

*};*

***38CH2533R2M3***

*Plot()*

*{*

*Outlier_Model("General",T(5),U(0,4),"t");*

*Sequence("38CH2533 Ring 2")*

*{*

*Boundary("Start: 38CH2533 Ring 2");*

*KDE_Plot("38CH2533 Ring 2 Units")*

*{*

*Sequence("Quadrat 110")*

*{*

*R_Date("UGAMS 53615", 3830, 25)*

*{*

*Outlier("General", 0.05);*

*};*

*R_Date("UGAMS 53616", 3780, 25)*

*{*

*Outlier("General", 0.05);*

*};*

*R_Date("OxA - 41061", 3854, 19)*

*{*

*Outlier("General", 0.05);*

*};*

*R_Date("UGAMS 53614", 3840, 25)*

*{*

*Outlier("General", 0.05);*

*};*

*R_Date("UGAMS 53613", 3730, 25)*

*{*

*Outlier("General", 0.05);*

*};*

*R_Combine("UGAMS 53612")*

*{*

*R_Date("UGAMS 53612a", 3760, 25);*

*R_Date("UGAMS 53612b", 3700, 25);*

*};*

*};*

*R_Date("OxA - 41062-N2140E1020", 3871, 19)*

*{*

*Outlier("General", 0.05);*

*};*

*Interval(LnN(ln(125),ln(2)));*

*};*

*Boundary("End: 38CH2533 Ring 2");*

*};*

*Difference("Lapse","Start: 38CH2533 Ring 2","End: 38CH2533 Ring 2");*

*};*
